# Kinetic gait analysis in healthy dogs and dogs with osteoarthritis: An evaluation of precision and overlap performance of a pressure-sensitive walkway and the use of symmetry indices

**DOI:** 10.1371/journal.pone.0243819

**Published:** 2020-12-15

**Authors:** Michelle Brønniche Møller Nielsen, Tenna Pedersen, Amalie Mouritzen, Anne Desiré Vitger, Lise Nikolic Nielsen, Helle Harding Poulsen, James Edward Miles

**Affiliations:** Department of Veterinary Clinical Sciences, University of Copenhagen, Frederiksberg, Denmark; University of Lincoln, UNITED KINGDOM

## Abstract

In veterinary practice, a thorough gait examination is essential in the clinical workup of any orthopedic patient, including the large population of dogs with chronic pain as a result of osteoarthritis. The traditional visual gait examination is, however, a subjective discipline, and systems for kinetic gait analysis may potentially offer an objective alternative for gait assessment by the measurement of ground reaction forces. In order to avoid unnecessary testing of patients, a thorough, stepwise evaluation of the diagnostic performance of each system is recommended before clinical use for diagnostic purposes. The aim of the study was to evaluate the Tekscan pressure-sensitive walkway system by assessing precision (agreement between repetitive measurements in individual dogs) and overlap performance (the ability to distinguish dogs with lameness due to osteoarthritis from clinically healthy dogs). Direction of travel over the walkway was investigated as a possible bias. Symmetry indices are commonly used to assess lameness by comparing ground reaction forces across different combinations of limbs in each dog. However, SIs can be calculated in several different ways and specific recommendations for optimal use of individual indices are currently lacking. Therefore the present study also compared indices in order to recommend a specific index preferable for future studies of canine osteoarthritis. Forty-one clinically healthy dogs and 21 dogs with osteoarthritis were included in the study. High precision was demonstrated. The direction of travel over the walkway was excluded as a possible bias. A significant overlap was observed when comparing ground reaction forces measured in dogs with osteoarthritis compared to clinically healthy dogs. In some affected dogs, symmetry indices comparing contralateral limbs differed from clinically healthy dogs, but in general, the overlap performance was insufficient and, consequently, general use of this method for diagnostic purposes in dogs with osteoarthritis cannot be recommended.

## Introduction

Canine patients are commonly presented in small animal veterinary practice with lameness of various degrees and causes. A thorough gait examination plays a key role in the investigation of these patients, and various lameness scoring scales have been recommended for a standardized grading of lameness in clinical practice [1, 2]. However, grading of lameness is a subjective discipline, and individual observers seem to develop unique scoring scales [3, 4].

To support the visual gait examination, ground reaction forces (GRFs) conducted by individual steps in canine locomotion can be measured using systems for kinetic gait analysis [5]. Force plate analysis is considered the gold standard for GRF measurements [5]. Single or multiple force plates placed in walkways or under treadmill belts are valuable tools for diagnostic kinetic gait analysis in both clinical and research settings [6]. Pressure measurement systems are another group of equipment for kinetic gait analysis. These systems consist of multiple pressure sensors and enable visualization of pressure distribution for individual paws as well as quantification of pressure, forces and temporal characteristics during gait [6]. A more in-depth review of kinetic gait analysis instruments has been published by Gillette et al. [6]. One group of commercially available pressure measurement systems is the pressure-sensitive walkway system (PSW), which permits sensitive and objective gait analysis by conversion of pressure measurements to GRFs [7]. Such systems are considered to be reliable and simple methods for evaluating, quantifying and monitoring lameness in dogs [8] with high diagnostic sensitivity and specificity [9]. The diagnostic potential of PSW has been shown to be comparable to force plate gait analysis [10], which itself has been shown to be superior to traditional visual gait examinations in regard to precision and reliability [11]. The diagnostic performance of PSW and force plate gait analysis has been extensively studied in clinically healthy dogs [12–17] and dogs with various causes of moderate or severe grades of lameness, e.g. dogs with hip dysplasia [7], cranial cruciate ligament rupture [8, 18, 19], experimentally-induced stifle arthritis [3], external fixation of tibial osteotomy [4], myelopathies [20], and various other orthopedic disorders [9].

Osteoarthritis (OA) is a condition affecting up to 20% of the adult dog population [21] and is a common cause of low-grade clinical lameness in dogs [2]. To the authors’ knowledge, clinical OA patients have previously been included in canine PSW studies to a very limited extent, thus motivating for further investigation in the field.

An important advantage of the PSW is that GRFs of all limbs are directly measured during each passage over the walkway. Thus, the PSW is minimally time-consuming for routine use in a busy clinical setting, and comfortable for patients, as all measurements can be obtained from relatively few gait cycles [22]. Because measurements of all limbs are performed simultaneously, data for calculation of symmetry indices (SIs) will also be directly available. SIs represent standardized comparisons of GRFs obtained from different individual limbs and results in a specific, sensitive, suitable and reliable assessment of unilateral limb dysfunction [19]. Thus, in patients with unilateral limb dysfunction, use of SIs eliminates the need to normalize data between subjects because the affected limb is compared to the clinically normal contralateral or ipsilateral limb [23]. OA is a chronic, progressive condition often affecting multiple joints and even though one specific limb is often more severely affected, contralateral or ipsilateral limbs might also be affected in clinical patients. Consequently, studies of SIs obtained by the PSW in canine OA patients are needed before the PSW can be recommended for routine clinical use in the diagnostic work-up of canine OA patients. Otherwise, the diagnostic performance of the PSW may be overestimated [24] with the risk of OA patients undergoing unnecessary testing [25]. A stepwise evaluation of diagnostic tests is generally recommended and often includes investigation of precision (i.e. the closeness of the measurements to each other) and overlap performance (i.e. the ability to differentiate diseased dogs from clinically healthy dogs) [26].

The aim of the present study was to assess the diagnostic performance of the Tekscan PSW (Tekscan I-Scan model 5101E VH4, Evolution) by evaluating the precision in clinically healthy dogs and in dogs with lameness due to OA, and the overlap performance differentiating dogs with low and moderate grades of lameness caused by previously diagnosed OA from clinically healthy dogs. In previous studies, factors such as different handlers, leash side [27] and cover type [28] have been shown to influence GRF results obtained by PSW. In the present study, direction of travel over the PSW was investigated as another potential source of bias.

Even though calculation of SIs is generally recommended [27] and extensively used for data presentation in canine PSW studies, SIs can be calculated in several different ways [8, 12, 15, 27, 29] and it is still not clear how gait symmetry is most optimally evaluated [12]. In the present study, several different symmetry indices were therefore calculated and compared in order to suggest recommendations of specific SIs for use in future PSW studies of OA in dogs.

The study was based on the following hypotheses: *The Tekscan PSW will be useful for diagnostic purposes in dogs with OA*, *showing high precision and indisputable overlap performance*. *Direction of passage over the walkway will not influence the results and calculation of specific SIs can be recommended in future studies of OA in dogs*.

## Materials and methods

Client-owned dogs with and without previously diagnosed OA were recruited by email contact to the staff of the department and by social media contact to the public. The study was performed at the University Hospital for Companion Animals, University of Copenhagen, Denmark during 2018 and 2019. The study protocol was approved by the institutional ethical committee at Department of Veterinary Clinical Sciences, University of Copenhagen, Denmark (approval number 2017–1). In the study dogs were walked over the pressure sensitive walk-way in loose leash by an experienced handler in a stress free environment. All dogs were allowed to acclimatize to the room and were subsequently walked across the walkway to become comfortable with the surroundings, the PSW, handler, and leash. No dogs were forced to walk on the walkway and the study did not result in any kind of animal sacrifice. Written consent was obtained from the owners.

### Clinically healthy dogs

Dogs were included as clinically healthy if the owner reported absence of lameness or other diseases, if there were no signs of local or systemic illness on a thorough clinical examination, if they presented with no visually detectable lameness, and if there was absence of abnormal findings on a thorough orthopedic examination. Other inclusion criteria were age between 2–7 years old, body mass of 15–40 kg, and body condition scores of 4–6. Dogs were not included as healthy if they received any medication or if they had a history of orthopedic disorders e.g. fractures or cruciate ligament rupture.

### Dogs with osteoarthritis

Dogs with OA were included in the study if they had a history of previously diagnosed OA and continuing clinical signs despite treatment with non-steroid anti-inflammatory therapy. OA was diagnosed by a veterinarian prior to inclusion in the study by clinical and radiological examinations at the authors’ clinic or at referring veterinary clinics. Thorough orthopedic examinations performed by one of the authors (JM) localized pain to one or more joints, with one limb identified as more severely affected compared to the others. Other inclusion criteria were body mass >25 kg and age>12 months (>18 months for giant breeds). Dogs were excluded from the study if there was any clinical suspicion of other local or systemic disease besides OA on thorough clinical examinations, owner histories and routine biochemistry, haematology and urine analyses. Additional invasive testing such as arthroscopy or synovial biopsy, or immunological studies, were not performed.

All dogs with OA were filmed while standing and walking for later visual gait analysis by the same author (JM). Based on clinical examinations and visual gait analysis, dogs were divided into 4 groups depending on the localization of the most severely affected joint; left thoracic (fore) limb (LF), left pelvic (hind) limb (LH), right thoracic (fore) limb (RF), and right pelvic (hind) limb (RH), respectively. Lameness was scored on an ordinal visual analogue scale from 1–5 defined as; no visual lameness (grade 0), mild lameness with minimal head/pelvic movements (grade 1), moderate lameness with normal stride length and partial weight bearing (grade 2), moderate lameness with reduced stride length and partial weight bearing (grade 3), severe lameness with minimal use of limb (grade 4), and non-weight bearing lameness (grade 5) [1, 2].

### Walkway measurements

The measurement system consisted of 4 Tekscan Medical #3140 sensors with a resolution of 1 sensel/cm^2^ incorporated in to a 1.95 m long by 0.45 m wide PSW, which was protected by a 3 m by 0.6 m cover throughout the study. Pressure data from activated sensels were transmitted to a computer running Tekscan software (Walkway 7.66) for conversion to vertical forces, enabling calculation of kinetic and temporal characteristics for each limb. All forces were normalized to body mass. The system was equilibrated to 75 Hz on a daily basis and the 4 sensors were calibrated using a phantom mass weighing 21.6 kg on a weekly basis. Data obtained from the PSW was subsequently exported to Microsoft Excel.

Before data collection, dogs were weighed on an electronic scale. All dogs were allowed to acclimate to the room and were subsequently walked across the walkway to become comfortable with the surroundings, the PSW, handler, and leash. All dogs were led at the right side in a loose leash by one of four experienced handlers, and leash side was kept constant during the study. Following acclimatization, 6 successive valid recordings were obtained for each dog while they walked the PSW, with 3 recordings in each direction of travel. A trial was considered valid when all 4 limbs fully contacted the PSW and the dog walked straight forward without stopping, hesitating, or having overt head movements. Velocity was controlled at 0.9–1.1 m/s. If velocity differed from these limits, the trial was repeated in order to ensure the same velocity in all patients allowing comparison of parameters across different individual dogs.

The following parameters were obtained from the system’s gait analysis software: Stance time, swing time, stride time, stride length, stride velocity, stride acceleration, peak vertical force, vertical impulse, and maximum peak pressure. To evaluate precision, 10 dogs were chosen randomly among those of the clinically healthy dogs, whose owners had no trouble bringing their dog back for an additional visit.

For all dogs several different symmetry indices were calculated, in order to investigate whether one specific index should be preferred in future PSW studies of dogs with OA. Diagonal (LF versus RH and RF versus LH), fore:hind (LF versus LH and RF versus RH), and left:right (LF versus RF and LH versus RH) SIs for each parameter were calculated, using 2 different approaches. Symmetry index (1) was calculated as simple ratios of measurements obtained by each limb, whereas SI (2) was calculated by the following equation modified from Schnabl-Feichter et al., 2018 [30]:

$$\text{SI}(2)=\text{abs}(\frac{\left(\text{Parameter}(\text{limb}1)-\text{Parameter}(\text{limb}2\right)}{\left(\text{Parameter}(\text{limb}1)+\text{Parameter}(\text{limb}2\right)})*100$$

From these calculations a SI (1) of 1 and a SI (2) of 0% would represent perfect paired limb symmetry. Symmetry indices were calculated for two parameters: maximum peak pressure and vertical impulse. Calculated SIs for dogs with OA were plotted graphically and compared with references for clinically healthy dogs. Using data from the clinically healthy dogs (S3 File), parametric reference intervals were calculated for SI (1) as mean±2SD, whereas non-parametric reference intervals were calculated for SI (2) using either the 2.5% and 97.5% percentiles or the 95% percentile, as appropriate.

### Statistical analysis

Microsoft Excel and GraphPad Prism were used for all analyses and a significance level <0.05 was considered significant.

Means with 95% confidence intervals (CI) and standard deviations (SD) were calculated for each parameter. D’Agostino & Pearson normality tests were used to confirm that kinetic data from clinically healthy dogs followed a normal distribution. Differences between ipsilateral and contralateral limbs in clinically healthy dogs were assessed by paired Student’s t-tests.

Data from 10 clinically healthy dogs were used to assess precision using coefficients of variation (CVs) calculated from 6 successive runs on the same day (intra-analytical CV) and averages of 6 successive runs on 2 separate days (inter-analytical CV), respectively.

Precision was assessed in dogs with OA by calculating intra-analytical CV based on data from all dogs included in the study.

Averaged data of all clinically healthy dogs from the 3 recordings obtained in each direction of travel were plotted together with the averaged data from all 6 recordings (both directions) for visual assessment of any possible difference. Differences between directions of travel were assessed by paired t-tests.

## Results

### Clinically healthy dogs

Sixteen male and 25 female clinically healthy dogs were included in the study. All dogs were medium or large breed dogs; 12 Labrador Retrievers, 6 Golden Retrievers, 2 Flat-coated Retrievers, 2 Border Collies, 2 German Shepherd dogs, 5 Crossbreed dogs, and 1 dog of each of 12 various breeds. The mean age was 4.0 years (range 2.0–6.9, SD 1.4) and the mean weight was 27.2 kg (range 15.2–38.8 kg, SD 6.8). Table 1 shows means, SDs and ranges of CVs of each kinetic parameter measured in clinically healthy dogs.

**Table 1:** Temporal characteristics and vertical ground reaction forces measured in clinically healthy dogs.

		Left thoracic limb	Right thoracic limb	Left pelvic limb	Right pelvic limb
**Stance Time (sec)**	**μ**	0.49	0.49	0.46	0.47
	**CI**	[0.48; 0.51]	[0.49; 0.51]	[0.44; 0.48]	[0.45; 0.49]
	**SD**	0.060	0.060	0.063	0.063
	**CV1**	1.9–5.5%	2.7–5.2%	2.3–8.6%	2.3–7.4%
	**CV2**	0.3–4.6%	0–4.2%	0.5–4.4%	0.2–5.6%
**Swing Time (sec)**	**μ**	0.29	0.29	0,33	0,33
	**CI**	[0.28; 0.30]	[0.29; 0.30]	[0.32; 0.34]	[0.32; 0.33]
	**SD**	0.031	0.029	0,02963	0,02802
	**CV1**	1.4–6.5%	1.9–5.4%	2.4–7.0%	1.2–8.6%
	**CV2**	0–5.1%	0–5.7%	0.4–3.3%	0–4.7%
**Stride Time (sec)**	**μ**	0.78	0.79	0.79	0.79
	**CI**	[0.76; 0.81]	[0,76; 0.81]	[0,76; 0.82]	[0,76; 0.81]
	**SD**	0.084	0.083	0.086	0.084
	**CV1**	1.2–5.5%	2.0–4.2%	1.8–7.0%	1.7–5.8%
	**CV2**	0.4–4.4%	0.2–4.9%	0.6–3.9%	0–4.0%
**Stride Length (cm)**	**μ**	78.3	78.3	77.4	77.7
	**CI**	[75.6; 80.9]	[75.6; 80.9]	[74.9; 70.9]	[75.1; 80.3]
	**SD**	8.32	8.43	7.98	8.15
	**CV1**	0.9–4.2%	1.7–3.1%	1.8–5.8%	1.1–4.7%
	**CV2**	0.3–3.4%	0–2.0%	0.01–2.6%	0.3–4.1%
**Stride Velocity (cm/sec)**	**μ**	100.1	99.7	98.5	99.0
	**CI**	[98.8; 101.4]	[98.5; 101.0]	[97.1; 99.9]	[97.5; 100.5]
	**SD**	4.17	3.95	4.43	4.80
	**CV1**	3.6–7.6%	2.9–9.6%	3.4–6.7%	2.9–7.7%
	**CV2**	0.5–7.0%	0.6–5.8%	0.05–6.0%	0.7–5.4%
**Peak Vertical Force (%BW)**	**μ**	54.4	53.7	32.4	31.9
	**CI**	[53.1; 55.8]	[52.4; 55.1]	[31.4; 33.3]	[30.8; 33.0]
	**SD**	4.21	4.36	3.17	3.42
	**CV1**	2.7–7.5%	2.3–6.0%	3.7–15.1%	2.1–11.9%
	**CV2**	1.5–6.7%	0.3–5.8%	1.0–9.1%	0.6–7.0%
**Peak Vertical Force (N)**	**μ**	141.1	139.2	83.9	82.8
	**CI**	[127.3; 154.8]	[125.5; 153.0]	[75.4; 92.4]	[74.4; 91.2]
	**SD**	43.6	43.5	27.0	26.7
	**CV1**	2.7–7.4%	2.3–6.0%	3.3–15.1%	2.1–11.9%
	**CV2**	1.4–6.7%	0.2–5.8%	1.0–8.6%	0.6–7.0%
Vertical Impulse (%BW*sec)	**μ**	19.9	19.8	10.6	10.6
	**CI**	[19.0; 20.9]	[18.8; 20.7]	[10.0; 11.2]	[10.0; 11.1]
	**SD**	3.01	2.98	1.77	1.63
	**CV1**	1.9–9.1%	2.8–8.3%	3.2-12-2%	2.9–11.1%
	**CV2**	0.5–7.9%	0.1–5.1%	1.0–14.3%	0.4–8.2%
Vertical Impulse (N*sec)	**μ**	52.7	52.3	28.2	28.1
	**CI**	[46.3; 59.1]	[45.9; 58.6]	[24.6; 31.9]	[24.6; 31.6]
	**SD**	20.3	20.0	11.7	11.2
	**CV1**	1.9–9.0%	2.7–8.3%	3.0–12.2%	2.9–11.2%
	**CV2**	0.5–7.9%	0.06–5.1%	1.1–14.3%	0.4–10.8%
**Maximum peak pressure (kPa)**	**μ**	115.0	113.7	82.1	82.2
	**CI**	[108.4; 121.6]	[107.4; 120.0]	[77.5; 86.7]	[78.0; 86.3]
	**SD**	20.9	20.0	14.6	13.1
	**CV1**	4.4–10.2%	3.9–8.5%	4.9–11.7%	3.1–13.0%
	**CV2**	0.8–4.2%	0.5–5.9%	0.2–7.5%	0.02–7.7%

Means (μ) with 95% confidence intervals [CI] and standard deviations (SD) for 8 different parameters measured in 41 clinically healthy dogs by the Tekscan pressure-sensitive walkway system. The range of coefficients of variation (CVs) based on 6 passages of 10 dogs on the same (intra-analytical CV, CV1) and 2 different days (inter-analytical CV, CV2), respectively are also included in the table.

**Fig 1:**
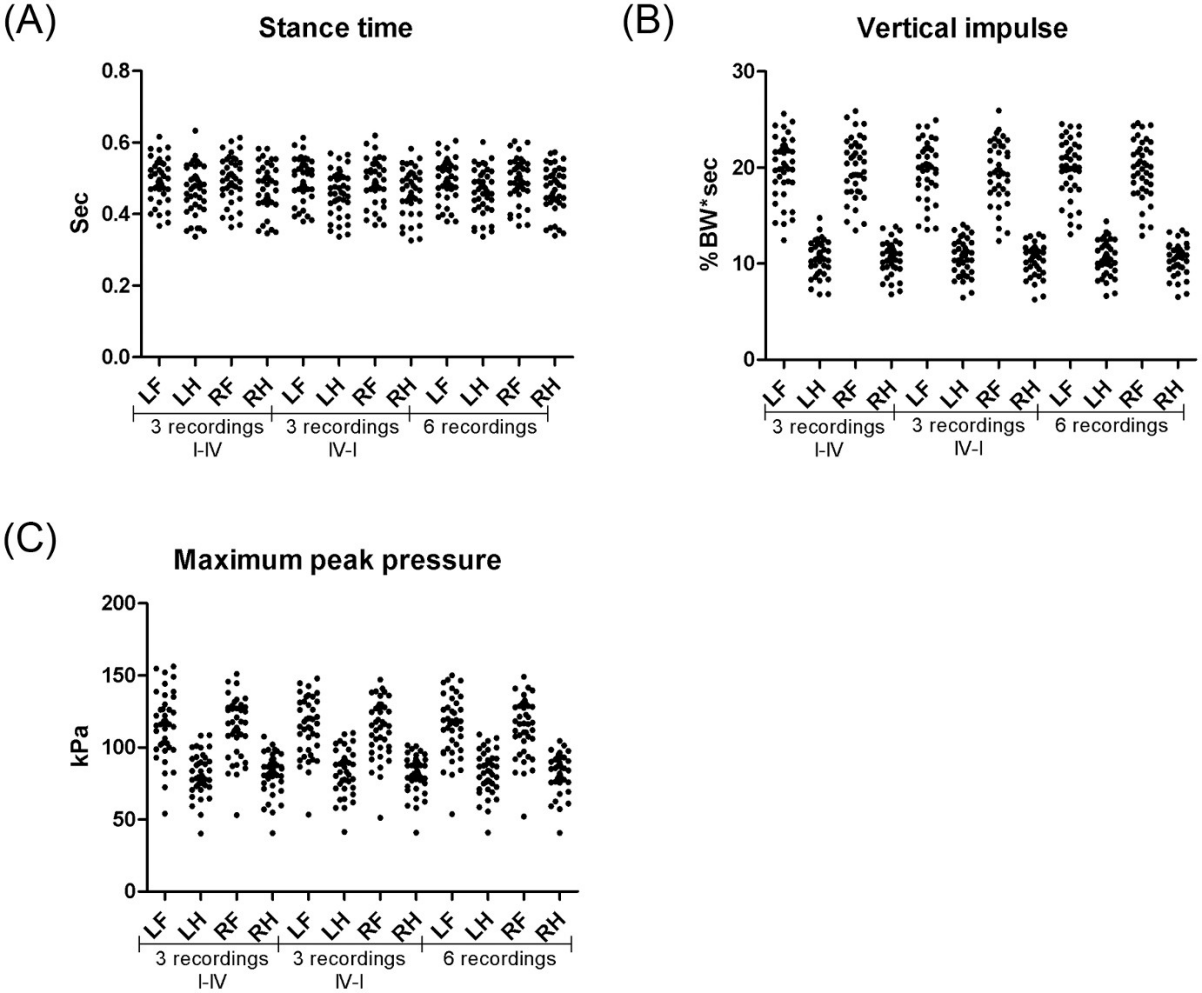
Ground reaction forces in clinically healthy dogs. Stance time (A), vertical impulse (B) and maximum peak pressure (C) measured in 41 clinically healthy dogs using the Tekscan pressure-sensitive walkway system. Following acclimatization, 6 successive valid recordings were obtained for each dog, with 3 recordings in each direction from sensor I to IV. Fore:hind symmetry of the stance time measurements was visually observed (A), whereas the vertical impulse (B) and maximum peak pressure (C) were asymmetric because of the increased weight load on the thoracic limbs compared to pelvic limbs in clinically healthy dogs. Left: right symmetry was visually observed for all parameters (A-C).

Comparable values of all parameters were observed between measurements obtained from contralateral thoracic and pelvic limbs, respectively (Fig 1, p = 0.42–1.0), whereas significantly higher peak vertical force, vertical impulse and maximum peak pressure were observed in thoracic limbs compared to the ipsilateral pelvic limbs (Fig 1, p<0.0001). Significantly higher swing times were observed for pelvic limbs compared to thoracic limbs (p<0.0001).

No significant differences were observed when comparing recordings in each direction (Fig 1, p = 0.52–0.99). High precision was observed, with intra-analytical CVs of 1.0–6.5% and inter-analytical CVs of 2.4–7.7%, respectively (Table 1).

### Dogs with lameness and previously diagnosed osteoarthritis

Twelve male and 9 female dogs with previously diagnosed OA were included in the study. All dogs were large breed dogs (10 Labrador Retrievers, 6 Golden retrievers, 2 German Shepherd dogs, 1 American Bulldog, 1 American Staffordshire Bull Terrier, and 1 Rottweiler) with a mean age of 9.3 years (range 3.6–13.6, SD 2,4) and a mean weight of 36.3 kg (range 27.2–48.5, SD 5,9). The distribution of the most severely affected limbs was: RF (n = 7), RH (n = 5), LF (n = 5), and LH (n = 4). Primary joints involved were the hip (n = 9), elbow (n = 10) and interdigital (n = 2) joints, with additional involvement of either contralateral or ipsilateral hip/ elbow and metacarpo- or metatarsophalangeal joints, based on clinical and radiographic examinations. The visual grading scores of lameness varied from low (Grade 1, n = 10 and grade 2, n = 7) to moderate (grade 3, n = 4). Table 2 shows means, SDs and ranges of CVs of each kinetic parameter measured in dogs presented with lameness and previously diagnosed OA.

**Table 2:** Temporal characteristics and vertical ground reaction forces measured in dogs previously diagnosed with osteoarthritis.

		Left thoracic limb	Right thoracic limb	Left pelvic limb	Right pelvic limb
**Stance Time (sec)**	**μ**	0.52	0.52	0.52	0.51
	**CI**	[0.50; 0.54]	[0.50; 0.55]	[0.48; 0.55]	[00.48; 0.55]
	**SD**	0.05	0.06	0.07	0.07
	**CV**	2.1–11.3%	2.1–12.2%	2.3–19.1%	1.5–21.6%
**Swing Time (sec)**	**μ**	0.28	0.28	0.31	0.31
	**CI**	[0.27; 0.3]	[0.27; 0.3]	[0.29; 0.33]	[0.29; 0.33]
	**SD**	0.03	0.03	0.04	0.04
	**CV**	3.04–7.5%	1.82–9.5%	2.45–19.7%	2.4–20.7%
**Stride Time (sec)**	**μ**	0.8	0.8	0.82	0.82
	**CI**	[0.76; 0.84]	[0.76; 0.84]	[0.78; 0.87]	[0.78; 0.86]
	**SD**	0.08	0.08	0.09	0.09
	**CV**	1.84–9.4%	2.3–10.0%	1.45–23.6%	1.9–25.4%
**Stride Length (cm)**	**μ**	76.5	77.0	77.0	76.8
	**CI**	[71.7; 81.3]	[72.1; 81.8]	[72.2; 81.2]	[72.2; 81.5]
	**SD**	10.5	10.6	9.8	10.2
	**CV**	1.46–8.2%	1.0–9.3%	1.0–17.4%	0.7–23.6%
**Stride Velocity (cm/sec)**	**μ**	95.6	96.2	93.6	94.2
	**CI**	[92.3; 99.0]	[92.9; 99.4]	[90.3; 97.0]	[90.8; 97.6]
	**SD**	7.3	7.1	7.4	7.5
	**CV**	2.0–16.5%	2.7–15.9%	1.6–15.5%	2.1–18.8%
**Peak Vertical Force (%BW)**	**μ**	77.8	72.8	43.9	45.5
	**CI**	[27.6; 128]	[32.7; 113]	[18.1; 69.7]	[18.0; 73.1]
	**SD**	110.3	88.3	56.6	60.6
	**CV**	3.4–14.8%	3.2–26.6%	3.8–27.8%	2.9–24.3%
**Peak Vertical Force (N)**	**μ**	191.7	189.1	111.0	113.8
	**CI**	[172.9; 210.6]	[171.6; 206.7]	[102.5; 119.6]	[105.2; 122.4]
	**SD**	41.5	38.6	18.8	18.8
	**CV**	3.1–11.8%	3.3–32.3%	2.35–34.7	4.2–27.7%
Vertical Impulse (%BW*sec)	**μ**	29.1	26.2	16.7	16.9
	**CI**	[9.7; 48.4]	[12.4; 40.0]	[6.4; 27.0]	[6.7; 27.1]
	**SD**	42.5	30.3	22.6	22.4
	**CV**	3.41–14.9%	3.2–26.6%	3.94–27.8%	3.1–24.3%
Vertical Impulse (N*sec)	**μ**	71.3	71.0	42.6	43.2
	**CI**	[62.4; 80.2]	[62.0; 79.9]	[36.7; 48.4]	[37.3; 49.2]
	**SD**	19.5	19.6	12.8	13.1
	**CV**	3.4–14.8%	3.15–26.6%	3.8–27.8%	2.9–24.3
**Maximum peak pressure (kPa)**	**μ**	129.4	125.4	95.2	95.8
	**CI**	[117.5; 141.3]	[115.3; 135.4]	[86.7; 103.7]	[86.9; 104.7]
	**SD**	26.2	22.1	18.7	19.6
	**CV**	2.2–12.2%	3.1–26.1%	3.8–24.3%	2.7–26.8%

Means (μ) with 95% confidence intervals [CI], standard deviations (SD) and ranges of intra-analytical coefficient of variations (CV) for 8 different parameters measured in 21 dogs with osteoarthritis using the Tekscan pressure-sensitive walkway system.

A wider range of intra-analytical CVs were observed in dogs previously diagnosed with OA (Table 2) compared to the clinically healthy dogs (Table 1). This increased variability was not restricted to the most severely affected limb but was observed for all limbs of dogs with OA (Table 3).

**Table 3:** Precision of ground reaction forces measured in individual limbs of dogs previously diagnosed with OA.

	Clinically affected limb	Ipsilateral limb	Contralateral limb	Diagonal limb
**Vertical impulse**	3.1–26.6%	4.8–24.3%	3.2–22.2%	3.4–27.8%
**Maximum peak pressure**	2.7–26.1%	2.2–26.8%	2.4–14.8%	3.4–24.3%

Vertical impulse and maximum peak pressure were measured using the Tekscan pressure sensitive walkway in 21 lame dogs comparing intra-analytical coefficient of variations (CV) based on measurements of the most severely clinically affected, ipsilateral, contralateral, and diagonal limbs, respectively.

### Overlap performance differentiating dogs with osteoarthritis from clinically healthy dogs

**Fig 2:**
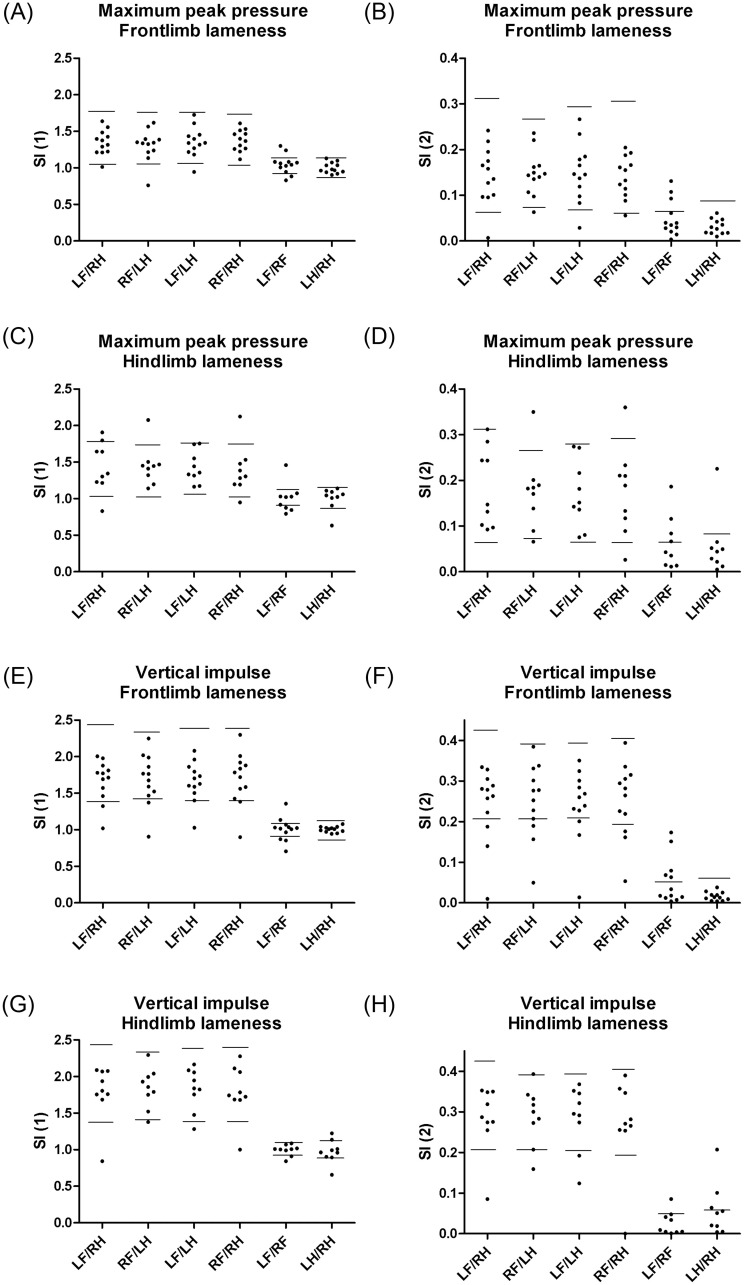
Comparison of different symmetry indices in dogs with osteoarthritis. Plots of Maximum peak pressure (A-D) and Vertical impulse (E-H) in 12 dogs with osteoarthritis (OA) with thoracic limb lameness (A-B and E-F) and 9 dogs with pelvic limb lameness (C-D and G-H), respectively. Measurements obtained from the Tekscan pressure-sensitive walkway system are presented as diagonal, fore:hind, and left:right symmetry indices (SI), respectively. SIs were calculated across right thoracic limb (RF), left thoracic limb (LF), right pelvic limb (RH), and left pelvic limb (LH) as simple ratios (SI (1), A,C,E,G) and as indices modified from Schnabl-Feichter et al., 2018 (SI (2), B,D,F,H), respectively.

Several of the SIs calculated in dogs with low- to moderate-grades of lameness and a previous diagnosis of OA were contained within the SI reference intervals based on measurements in clinically healthy dogs (Fig 2), and differing SIs were only observed in low proportions of dogs with OA (Table 4). However, more deviation from the healthy dogs were observed, when comparing left:right SIs to diagonal and fore:hind indices (Fig 2 and Table 4), respectively, indicating higher diagnostic sensitivity for the left:right SIs.

**Table 4:** Proportions of dogs with osteoarthritis and symmetry indices differing from clinically healthy dogs.

		Diagonal SIs		Fore:hind SIs		Left:right SIs	
		LF/RH	RF/LH	LF/LH	RF/RH	LF/RF	LH/RH
**Vertical impulse**	**SI (1)**	0.1 (3/21)	0.1 (3/21)	0.1 (2/21)	0.1 (3/21)	0.3 (7/21)	0.05 (1/21)
	**SI (2)**	0.2 (3/21)	0.2 (4/21)	0.2 (5/21)	0.1 (3/21)	0.3 (5/21)	0.1 (3/21)
**Maximum peak pressure**	**SI (1)**	0.2 (4/21)	0.1 (2/21)	0.05 (1/21)	0.1 (2/21)	0.4 (8 /21)	0.05 (1/21)
	**SI (2)**	0 (0/21)	0.1 (3/21)	0.05 (1/21)	0.1 (3/21)	0.3 (7/21)	0.05 (1/21)

Diagonal, fore:hind, and left:right symmetry indices (SI) were obtained from the Tekscan pressure-sensitive walkway system. SIs were calculated across right thoracic limb (RF), left thoracic limb (LF), right pelvic limb (RH), and left pelvic limb (LH). SI (1) was calculated as simple ratios, whereas SI (2) was obtained by calculations modified from Schnabl-Feichter et al., 2018 [30], respectively.

Reference intervals calculated from measurements of 41 healthy dogs are presented as horizontal lines. Parametric intervals for SI (1) were calculated as the mean±2sd, whereas nonparametric intervals for SI (2) were calculated using the 2.5% and 97.5% percentiles, or the 95% percentile, as appropriate.

Whereas most diagonal and fore:hind SIs obtained from OA dogs were contained within the reference SIs, the left:right SIs subjectively tended to show more variation.

**Fig 3:**
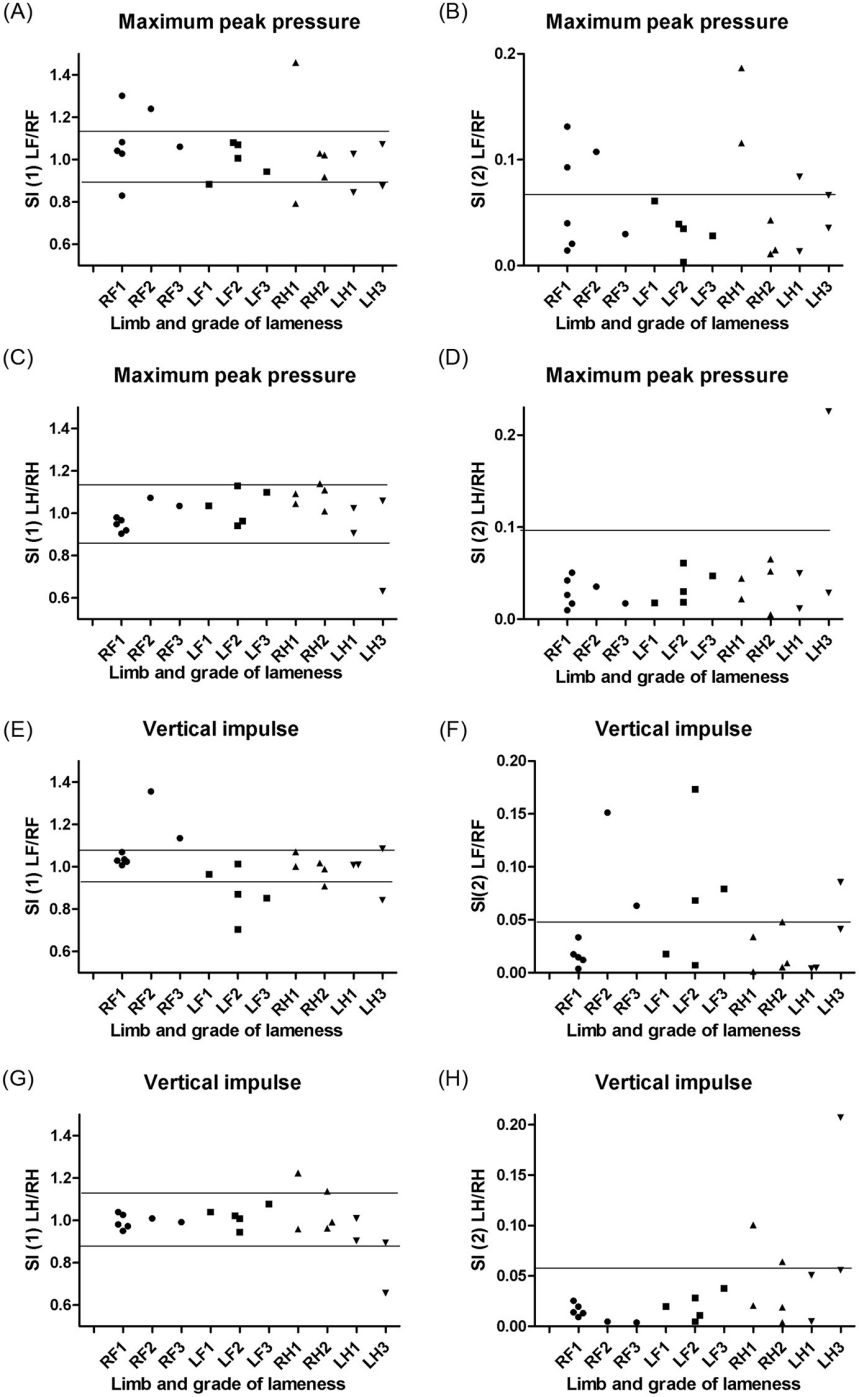
Symmetry indices in dogs with osteoarthritis comparing different grades and limbs of lameness. Plots of left:right symmetry indices (SIs) of maximum peak pressure (A-D) and vertical impulse (E-H) in 21 dogs with osteoarthosis (OA) and 41 clinically healthy dogs obtained from the Tekscan pressure-sensitive walkway system (PSW). Dogs were divided in groups by the visual grade of lameness (0–5) of left (L) or right (R), thoracic (fore—F) and pelvic (hind—H) limb, respectively. SIs were calculated as simple ratios (SI (1), A,C,E,G) and as indices modified from Schnabl-Feichter et al., 2018 (SI (2), B,D,F,H), respectively. Using data from the clinically healthy dogs, parametric reference intervals were calculated for SI (1) as mean±2SD, whereas non-parametric reference intervals were calculated for SI (2) using either the 2.5% and 97.5% percentiles or the 95% percentile, as appropriate. Calculated reference intervals are presented as horizontal lines.

Fig 3 illustrates the calculated left:right SIs of maximum peak pressure (Fig 3A–3E) and vertical impulse (Fig 3F–3H) in dogs with different grades of lameness (1–3) on various limbs. Reference intervals for SI (1) and SI (2) are shown as horizontal lines, and was obtained from clinically healthy dogs as described above. A larger proportion of the higher grades of lameness was detected using SI (1) LF/RF, but none of the left:right SIs were found to be useful in the detection of all grades of lameness in dogs with OA (Table 5).

**Table 5:** Proportion of dogs with lameness having symmetry indices (SI) deferring from the references of clinically healthy dogs.

		SI (1)		SI (2)	
		LF/RF	LH/RH	LF/RF	LH/RH
**Maximum peak pressure**	Grade 1	0.6 (6/10)	0 (0/10)	0.5 (5/10)	0 (0/10)
	Grade 2	0.1 (1/7)	0 (0/7)	0.1 (1/7)	0 (0/7)
	Grade 3	0.25 (1/4)	0.25 (1/4)	0 (0/4)	0.25 (1/4)
**Vertical impulse**	Grade 1	0 (0/10)	0.1 (1/10)	0 (0/10)	0.1 (1/10)
	Grade 2	0.6 (4/7)	0.1 (1/7)	0.4 (3/7)	0.1 (1/7)
	Grade 3	1 (4/4)	0.25 (1/4)	0.75 (3/4)	0.25 (1/4)

Left:right SIs are based on measurements of maximum peak pressure and vertical impulse obtained from the Tekscan pressure-sensitive walkway system. SIs were calculated across right thoracic limb (RF), left thoracic limb (LF), right pelvic limb (RH), and left pelvic limb (LH). SI (1) was calculated as simple ratios, whereas SI (2) was obtained by calculations modified from Schnabl-Feichter et al., 2018 [30].

## Discussion

Data obtained from clinically healthy dogs in the present study were comparable to previous studies showing symmetric data when comparing contralateral thoracic or pelvic limbs and significantly more weight bearing on the thoracic limbs compared to pelvic limbs [29].

The study showed an acceptable analytical performance of the Tekscan PSW regarding precision. Lower CVs were found in the present study, compared to previous PSW and force plate studies, showing CVs up to 14–30% [27, 31–33], even though higher CVs were observed in dogs with OA compared to clinically healthy dogs. Handlers, leash side, cover material, and velocity were kept constant in the present study to avoid unnecessary influence, as previously recommended by other authors [13, 27, 28]. In contrast to a smaller pilot study [34], the results were not influenced by the direction of travel (Fig 2).

Narrow 95% CIs and low SDs were calculated for each PSW variable (Table 1) indicating that it should be possible to establish narrow reference intervals for each parameter in clinically healthy dogs. However, local validation of such intervals should be considered on an institutional level.

A large overlap between SIs calculated for clinically healthy and OA dogs was observed (Figs 2 and 3) and SIs only differed from clinically normal dogs for a limited number of dogs with OA (Tables 3 and 4). Possible influencing factors could be breed, age, and weight differences between included dogs with and without OA, as well as lameness severity and multiple joint involvement. Each group contained a mixture of breeds and was considered broadly representative of the general population of large breed dogs presented to our practice. However, breed variation has been described [15, 17, 35] and even though Labrador and Golden Retrievers were the dominant breeds in both groups of dogs, several other breeds of dogs were included, not necessarily with comparable GRFs. Differences in age and weight distribution between groups could be other influencing factors. The dogs with OA were older than the clinically healthy dogs. Age variation of GRF variables has been proposed to explain variation between dogs [35], but studies specifically addressing this question are lacking. Unfortunately, the high incidence of OA in the older dog population [21] could make it challenging to find reference dogs with a comparable age distribution but without clinical or subclinical OA. The included dogs with OA had higher body mass compared to the group of clinically healthy dogs. Differences in body mass could result in morphological variation and variation in preferred velocity, and these are other factors previously shown to affect GRFs [23]. In humans, obesity is a well-known risk factor for developing OA [36] and obesity and overweight are common in dogs with OA [37]; finding reference dogs with a comparable weight distribution, but without clinical or subclinical OA might also be challenging. Subclinical osteoarthritis in the clinically healthy dogs included in our study was not excluded radiographically, and could represent a possible bias. However, clinical lameness and pain were systematically ruled out by thorough clinical examinations and owner histories, thus minimizing the practical importance of this potential source of bias. Further studies are needed in order to investigate whether reduced heterogeneity of the included dogs could result in less overlap of SIs between groups, e.g. by defining breed-specific reference intervals.

As previously mentioned, the nature of OA could be another influencing factor. Whereas gait SIs are specific, sensitive, suitable and reliable to assess unilateral limb dysfunction (8), clinical OA commonly affects more than one joint. In the present study, OA dogs were divided in groups based on the most severely affected limb, but OA affecting more than one joint could potentially result in dysfunction of more than one limb, thus influencing calculated SIs.

In the present study several SIs were compared in order to make recommendations for calculations of specific SIs in future studies of osteoarthritis. Because of the large overlap between SIs calculated in the two groups of dogs, the system and analysis used here cannot be recommended for identifying clinical OA patients with low to moderate grade lameness. However, studies in other groups of orthopedic patients using SIs obtained by the Tekscan PSW will still be relevant and the potential use in longitudinal studies of OA patients may also be an interesting field for further investigation. As larger proportions of OA dogs were detected using SI (1) LF/RF for vertical impulse (Table 5), this specific index might be useful in such studies.

We used clinical examination and visual gait analysis by an experienced orthopedic surgeon as the golden standard for assessment of lameness and pain in dogs. However, the visual analogue lameness scale may not be the most optimal comparison variable for future PSW studies [3]. In the present study lameness was graded by the same experienced orthopedic surgeon, taking into account that an individual observer seems to have an individual unique lameness scoring scale [4]. However, grading of lameness is a subjective discipline and low agreement between visual assessment of lameness and GRFs has previously been demonstrated in force plate studies, unless investigated dogs were severely lame [3]. Higher agreement might be expected in PSW studies compared to measurements obtained by force plate gait analysis because higher sensitivity and specificity of measurements of GRF could be expected using the PSW [10, 22, 38]. However, these assumptions remain to be confirmed.

Accurately designed and reported studies of diagnostic performance are necessary for safe implementation of diagnostic tests for general clinical use [39] and further evaluation of the diagnostic performance of the PSW in dogs is still needed, before the PSW can be recommended for routine diagnostic workup in canine orthopedic patients. The present study represents some of the important initial steps recommended for thorough evaluations of a diagnostic test [26]. Based on the large overlap between mildly to moderately lame dogs previously diagnosed with OA and clinically healthy dogs, further validation of the diagnostic performance in this group of patients is, however, not recommended.

## Conclusion

The Tekscan PSW measures ground reaction forces in clinically healthy dogs with high precision and the results are not influenced by the direction of passage over the walkway. However, the study showed a large overlap of measured ground reaction forces in mildly to moderately lame dogs with OA compared to clinically healthy dogs. Even though one specific symmetry index was more useful in the detection of lameness compared to other calculated indices, the SIs obtained from most dogs with OA were contained within the reference intervals obtained from clinically healthy dogs. Based on the present study, the system and analysis used here cannot be recommended as a diagnostic test for detection of abnormal gait in dogs with OA.

## Supporting information

S1 File Ground reaction forces in clinically healthy dogs. Fig 1 is based on walkway measurements obtained from clinically healthy dogs. Measurements of stance time (1A), vertical impulse (1B), and maximum peak pressure (1B) are presented in this supplementary file comparing data obtained from different legs of 41clinically healthy dogs walking in different directions.
(PDF)

S2 File Comparison of different symmetry indices in dogs with osteoarthritis. Fig 2 is based on symmetry indices calculated from measurements of maximum peak presure (A-D) and vertical impulse (E-H) of different legs in dogs with osteoarthritis. 2 different symmetry indices (SI1 (A, C, E, G) and SI2 (B, D, F, H)) are compared in 12 dogs with thoracic limb (A, B, E, F) and 9 dogs with pelvic limb lameness (C, D, G, H), respectively.
(PDF)

S3 File Symmetry indices in dogs with osteoarthritis comparing different grades and limbs of lameness. Fig 3 is based on left:right symmetry indices (SIs) of Maximum peak pressure (A-D) and Vertical impulse (E-H) measured in 21 dogs with osteoarthritis (OA) and 41 clinically healthy dogs, respectively. Dogs were divided in groups by the visual grade of lameness (0–5) of left (L) or right (R), thoracic (fore—F) and pelvic (hind—H) limb, respectively. SIs were calculated as simple ratios (SI (1), A,C,E,G) and as indices modified from Schnabl-Feichter et al., 2018 (SI (2), B,D,F,H). Using data from the clinically healthy dogs, parametric reference intervals were calculated for SI (1) as mean±2SD, whereas non-parametric reference intervals were calculated for SI (2) using either the 2.5% and 97.5% percentiles or the 95% percentile, as appropriate.
(PDF)

S4 File Temporal characteristics and vertical ground reaction forces measured in clinically healthy dogs. The calculated means and standard deviation listed in Table 1 are based on 6 walkway measurements of each limb of 41 clinically healthy dogs. Temporal characteristics and measured vertical ground reaction forces are listed in the present file.
(PDF)

S5 File Temporal characteristics and vertical ground reaction forces measured in dogs with osteoarthritis. The calculated means and standard deviation listed in Table 2 are based on 6 walkway measurements of each limb of 21 dogs with osteoarthritis. Temporal characteristics and measured ground reaction forces are listed in the present file.
(PDF)

S6 File Precision of vertical impulse and maximum peak pressure measured in individual limbs of dogs previously diagnosed with osteoarthritis. Coefficients of variation calculated for repeated measurements of vertical impulse and maximum peak pressure in 21 dogs with osteoarthritis. In Table 3 precision is compared across limbs affected by osteoarthritis compared with contralateral, ipsilateral and diagonal limbs.
(PDF)

S7 File Precision of temporal characteristics and vertical ground reaction forces in clinically healthy dogs. Inter- and intraanalytical coefficients of variation calculated for individual limbs measured in 10 clinically healthy dogs.
(PDF)

S8 File Precision of temporal characteristics and vertical ground reaction forces in 21 dogs with osteoarthritis. Intraanalytical coefficients of variation calculated for individual limbs in 21 dogs with osteoarthritis.
(PDF)
