# Supplementary material for: Kinetic gait analysis in healthy dogs and dogs with osteoarthritis: An evaluation of precision and overlap performance of a pressure-sensitive walkway and the use of symmetry indices
Source: PLoS One. 2020 Dec 15;15(12):e0243819. doi: 10.1371/journal.pone.0243819 (PMC7737891; doi:10.1371/journal.pone.0243819)
Supplement: S1 File — Fig 1 is based on walkway measurements obtained from clinically healthy dogs. Measurements of stance time (1A), vertical impulse (1B), and maximum peak pressure (1B) are presented in this supplementary file comparing data obtained from different legs of 41clinically healthy dogs walking in different directions. (PDF) [file pone.0243819.s001.pdf]

S1 File. Ground reaction forces in clinically healthy dogs

Fig 1 is based on walkway measurements obtained from clinically healthy dogs.
Measurements of stance time (1A), vertical impulse (1B), and maximum peak pressure (1B) are presented in this supplementary file.
Data obtained from different legs of 41clinically healthy dogs walking in different directions are compared.

Abbreviations:

RF	Measured ground reaction forces in right thoracic limb
RH	Measured ground reaction forces in right pelvic limb
LF	Measured ground reaction forces in left thoracic limb
LH	Measured ground reaction forces in left pelvic limb

1A
Stance Time, sec

Average of 3 recordings in direction I-IV				*Average of 3 recordings in direction IV-I*				*Average of 6 recordings*			
LF	LH	RF	RH	LF	LH	RF	RH	LF	LH	RF	RH
0,54333	0,54	0,57333	0,5533333	0,55333	0,50667	0,54	0,5033333	0,54833	0,52333	0,55667	0,52833
0,4	0,35333	0,36333	0,3466667	0,39333	0,35333	0,37	0,3466667	0,39667	0,35333	0,36667	0,34667
0,47	0,40667	0,48667	0,44	0,47	0,41667	0,48667	0,4366667	0,47	0,41167	0,48667	0,43833
0,44333	0,42667	0,44333	0,4266667	0,46333	0,43	0,46667	0,43	0,45333	0,42833	0,455	0,42833
0,43333	0,42	0,43333	0,4433333	0,41	0,40333	0,41	0,4033333	0,42167	0,41167	0,42167	0,42333
0,54	0,5	0,55333	0,5366667	0,53	0,48	0,52333	0,5133333	0,535	0,49	0,53833	0,525
0,49	0,47	0,48333	0,47	0,51	0,45667	0,49	0,4466667	0,5	0,46333	0,48667	0,45833
0,55333	0,54	0,55667	0,5033333	0,52667	0,51	0,52333	0,51	0,54	0,525	0,54	0,50667
0,49	0,48	0,51667	0,4966667	0,47667	0,46	0,49333	0,48	0,48333	0,47	0,505	0,48833
0,47333	0,44	0,48	0,4533333	0,47	0,44	0,47333	0,4433333	0,47167	0,44	0,47667	0,44833
0,47	0,45667	0,47333	0,4366667	0,51	0,45667	0,49667	0,45	0,49	0,45667	0,485	0,44333
0,47333	0,43667	0,47	0,4266667	0,47333	0,44	0,47	0,4366667	0,47333	0,43833	0,47	0,43167
0,52333	0,49667	0,53667	0,5166667	0,56	0,52	0,55667	0,5333334	0,54167	0,50833	0,54667	0,525
0,52667	0,48667	0,54333	0,4866667	0,51667	0,43333	0,51667	0,4733333	0,52167	0,46	0,53	0,48
0,50667	0,44667	0,50667	0,4666667	0,45	0,39333	0,43333	0,4	0,47833	0,42	0,47	0,43333
0,54667	0,53333	0,54667	0,5533333	0,54667	0,54667	0,54333	0,5433334	0,54667	0,54	0,545	0,54833
0,41333	0,36	0,40333	0,35	0,41667	0,36333	0,42	0,36	0,415	0,36167	0,41167	0,355

0,47	0,45333	0,47	0,4366667	0,54333	0,5	0,55667	0,5	0,50667	0,47667	0,51333	0,46833
0,36667	0,36	0,37	0,3666667	0,39333	0,37	0,37	0,3633333	0,38	0,365	0,37	0,365
0,61667	0,63333	0,61333	0,5833333	0,59333	0,57	0,58667	0,5566667	0,605	0,60167	0,6	0,57
0,48333	0,41333	0,5	0,4333333	0,48	0,47333	0,46667	0,4666667	0,48167	0,44333	0,48333	0,45
0,58667	0,56333	0,60333	0,5833333	0,55333	0,53	0,56	0,5266666	0,57	0,54667	0,58167	0,555
0,51667	0,48333	0,52667	0,4833333	0,52667	0,49667	0,52	0,49	0,52167	0,49	0,52333	0,48667
0,47667	0,45	0,46	0,4466667	0,47333	0,44333	0,47	0,4433333	0,475	0,44667	0,465	0,445
0,45667	0,41667	0,47667	0,4333333	0,43667	0,39	0,43667	0,4	0,44667	0,40333	0,45667	0,41667
0,49667	0,46333	0,48	0,4533333	0,48333	0,45	0,47333	0,4366667	0,49	0,45667	0,47667	0,445
0,39667	0,35333	0,39	0,3566667	0,4	0,37	0,4	0,3666667	0,39833	0,36167	0,395	0,36167
0,54333	0,53333	0,53	0,52	0,52	0,5	0,50333	0,4966667	0,53167	0,51667	0,51667	0,50833
0,51333	0,48333	0,51333	0,4933333	0,53667	0,5	0,54	0,5166667	0,525	0,49167	0,52667	0,505
0,5	0,47333	0,49	0,5	0,51333	0,47333	0,51	0,4933333	0,50667	0,47333	0,5	0,49667
0,53333	0,50667	0,54333	0,5233333	0,52333	0,47333	0,52	0,48	0,52833	0,49	0,53167	0,50167
0,58333	0,53	0,58667	0,5433334	0,58667	0,55667	0,59667	0,5433334	0,585	0,54333	0,59167	0,54333
0,37667	0,33667	0,39	0,3533333	0,38333	0,33667	0,38667	0,3266667	0,38	0,33667	0,38833	0,34
0,58	0,54667	0,58667	0,5633333	0,61333	0,56667	0,62	0,5833333	0,59667	0,55667	0,60333	0,57333
0,52333	0,50333	0,51	0,5033333	0,53667	0,50667	0,54	0,5166667	0,53	0,505	0,525	0,51
0,56333	0,55	0,55333	0,5533333	0,55667	0,52667	0,55333	0,54	0,56	0,53833	0,55333	0,54667
0,40333	0,36	0,41333	0,38	0,38	0,34333	0,38333	0,33	0,39167	0,35167	0,39833	0,355
0,50667	0,47667	0,5	0,4933333	0,49	0,47667	0,48667	0,46	0,49833	0,47667	0,49333	0,47667
0,55667	0,53667	0,56	0,53	0,55333	0,50333	0,53333	0,5133333	0,555	0,52	0,54667	0,52167
0,44	0,39667	0,45333	0,43	0,46667	0,43667	0,47667	0,45	0,45333	0,41667	0,465	0,44
0,49	0,49667	0,49333	0,4933333	0,47	0,45667	0,48	0,47	0,48	0,47667	0,48667	0,48167

1B

Vertical Impulse, %BW*sec

Average of 3 recordings in direction I-IV				*Average of 3 recordings in direction IV-I*				*Average of 6 recordings*			
LF	LH	RF	RH	LF	LH	RF	RH	LF	LH	RF	RH
24,8	11,4667	25,2333	12,03333	24,3	11,2667	23,6	11,6	24,55	11,3667	24,4167	11,8167
14,5333	7,33333	14,1333	7,766667	16,0667	8,63333	13,6333	8,166667	15,3	7,98333	13,8833	7,96667

16,2333	10,7667	17,4667	11,73333	16,6667	10,8333	17,2333	11,63333	16,45	10,8	17,35	11,6833
17,2333	11,3	16,8667	10,83333	18,2	12,0667	17,7667	10,63333	17,7167	11,6833	17,3167	10,7333
14,1333	8,96667	14,3667	10	13,5333	9,1	13,2	8,9	13,8333	9,03333	13,7833	9,45
21,5	12,5	21,5667	13,03333	21,1333	12,6667	19,3333	12,8	21,3167	12,5833	20,45	12,9167
19,7	10,2333	19,5667	10,13333	20,3	10,3333	19,2667	10,36667	20	10,2833	19,4167	10,25
21,7667	11,9333	22,2333	10,53333	20,2667	11,1	21,5667	11,26667	21,0167	11,5167	21,9	10,9
19,8667	10,4667	21,2	11,4	19,8	8,7	18,5	11,63333	19,8333	9,58333	19,85	11,5167
19,8333	9,63333	21,0333	8,5	19,4333	9,93333	19,7	9,433333	19,6333	9,78333	20,3667	8,96667
21,6667	11,0333	22,5667	9,666667	24,9333	11,2333	23,3	10,13333	23,3	11,1333	22,9333	9,9
19,8667	8,93333	19,2667	7,966667	20,4	8,66667	19,1	8,233334	20,1333	8,8	19,1833	8,1
21,8667	13,5667	20,4333	13,7	21,9333	13	22,7	12,86667	21,9	13,2833	21,5667	13,2833
22,9	10,4333	23,1333	11,03333	21,8333	9,33333	22,2	10,86667	22,3667	9,88333	22,6667	10,95
18,5667	9,83333	19,2333	10,3	16,7667	8,73333	15,8667	9,066667	17,6667	9,28333	17,55	9,68333
22,3	12,1333	23,1	13,46667	22,5333	13,5	22,8667	12,7	22,4167	12,8167	22,9833	13,0833
18,4667	8,6	16,8667	8,9	17,8333	8,16667	17,7667	8,533334	18,15	8,38333	17,3167	8,71667
18,4667	11,4667	17,5	11,3	21,3	12,3	20,7	11,63333	19,8833	11,8833	19,1	11,4667
12,4333	6,83333	13,4667	7,133333	13,6667	6,96667	12,3667	6,6	13,05	6,9	12,9167	6,86667
23,2	14,7667	22,7333	13,86667	22	14,0667	21,1667	13,06667	22,6	14,4167	21,95	13,4667
19,0667	9,16667	19,1333	9,566667	18,8333	10,8667	17,4	11,3	18,95	10,0167	18,2667	10,4333
24,4	12,0333	25,9	12,16667	23	11,5333	22,7667	11,16667	23,7	11,7833	24,3333	11,6667
21,6333	10,6	21,5	10,83333	21,6667	11,1333	21,7667	10,96667	21,65	10,8667	21,6333	10,9
20,4333	10,3333	18,8667	9,466666	18,7667	9,66667	18,1667	9,766666	19,6	10	18,5167	9,61667
15,3333	10,4667	17,5333	11	14,6667	10,4	16,2333	10,3	15	10,4333	16,8833	10,65
19,8	9,73333	19,2333	10,26667	19,6667	10,4333	18,6	9,4	19,7333	10,0833	18,9167	9,83333
15,3667	8,33333	15,9333	7,866667	15,7333	8,1	15,9333	7,8	15,55	8,21667	15,9333	7,83333
22,3333	12,5667	22,2	11,5	22	12,0333	20,3	11,66667	22,1667	12,3	21,25	11,5833
19,5	10,6333	18,6	11,03333	20,0667	10,3667	21,5667	11,36667	19,7833	10,5	20,0833	11,2
20,1333	10,2333	19,2	10,43333	20,2	10,9	19,7333	10,8	20,1667	10,5667	19,4667	10,6167
21,6667	11,1333	21,5667	12,1	20	10,8667	19,7667	11,16667	20,8333	11	20,6667	11,6333
23,7	11,7667	24,5667	11,93333	23,1667	13,2667	23,9667	11	23,4333	12,5167	24,2667	11,4667
14,2333	8,36667	15,5667	9,566667	13,9	8,33333	14,7667	8,7	14,0667	8,35	15,1667	9,13333
24,2667	12,4667	23,3667	12,36667	24,3	13,7333	25,9333	11,5	24,2833	13,1	24,65	11,9333

20,8667	12,1333	20,4333	11,06667	21,1667	11,6333	22,3	11,73333	21,0167	11,8833	21,3667	11,4
22,9	12,7667	22,2	11,83333	23,3667	12,2	23	12,33333	23,1333	12,4833	22,6	12,0833
18,5333	6,8	18,3333	6,8	18,1333	6,46667	17,3	6,266667	18,3333	6,63333	17,8167	6,53333
22	9,8	21	10,4	20,3	10,1	19,5	10,06667	21,15	9,95	20,25	10,2333
25,6	12,2333	24,5333	11,83333	23	12,8667	22,2667	11,66667	24,3	12,55	23,4	11,75
17,3	8,23333	17,4667	9,7	18,4333	9,13333	19,4	9,7	17,8667	8,68333	18,4333	9,7
20,6333	11,2	20,6	10,73333	19,8	10,3667	19,5667	10,7	20,2167	10,7833	20,0833	10,7167

1C

Maximum peak pressure, kPa

Average of 3 recordings in direction I-IV				***Average of 3 recordings in direction IV-I***				***Average of 6 recordings***			
LF	LH	RF	RH	LF	LH	RF	RH	LF	LH	RF	RH
144,3	87,7667	127,133	87,66666	137,867	88,1	138,267	91,23333	141,083	87,9333	132,7	89,45
72,4	53,2333	81,8667	54,86666	89,8667	58	86,1333	59,5	81,1333	55,6167	84	57,1833
116,333	88,4	117,6	98,43333	120,133	86,4667	114,867	96,56667	118,233	87,4333	116,233	97,5
116,033	89,7333	111,767	86,23333	131,867	92,7667	105,567	87,46667	123,95	91,25	108,667	86,85
102,933	93,1667	109,133	91,46667	107,067	91,3667	111,833	88,86667	105	92,2667	110,483	90,1667
126,6	84,1333	117,933	80,56667	126	89,4667	115,433	90,46667	126,3	86,8	116,683	85,5167
100,167	94,7667	97,1	84,96667	98,2	90,2667	97,4	86,46667	99,1833	92,5167	97,25	85,7167
103	77	112,333	76,33334	101,333	76,3333	108,667	80,33334	102,167	76,6667	110,5	78,3333
116,2	77,8333	109,467	88,8	109,633	67,4	105,7	97,7	112,917	72,6167	107,583	93,25
101,9	70,5333	93,8333	57,1	91,0667	71,3667	93,5	68,06667	96,4833	70,95	93,6667	62,5833
113,1	83,7	131,7	84	121,2	82,9333	124,967	87,73333	117,15	83,3167	128,333	85,8667
98,8	64,6333	93,1	59,66667	94,7333	61,9667	90,7	62,36666	96,7667	63,3	91,9	61,0167
156,3	108,6	138,133	95,5	136,433	104,567	140,933	90,93333	146,367	106,583	139,533	93,2167
149,067	79	133,333	85,36667	144,633	83	127,067	89,2	146,85	81	130,2	87,2833
82,1	63,9667	85,5333	84,73333	86,5	63,8	79,5333	70,43333	84,3	63,8833	82,5333	77,5833
152,033	99,9	151,067	97,33334	147,967	109,267	147,1	95,46667	150	104,583	149,083	96,4
98,4	73,0667	81,3	71,3	93,5333	64,2667	82,5333	64,33334	95,9667	68,6667	81,9167	67,8167
138,7	100,3	128	102,0333	129,433	98,0667	127,867	100,9	134,067	99,1833	127,933	101,467
54	40,3	53,1	40,6	53,3667	41,4	51,1667	40,96667	53,6833	40,85	52,1333	40,7833

129,9	92,1667	129,067	92	120,233	104,633	129,933	91,6	125,067	98,4	129,5	91,8
122,033	79,4	125,867	88,36667	114,6	88,5333	116,267	87,43333	118,317	83,9667	121,067	87,9
136,433	108,267	145,7	107,6	138,767	110,067	136,1	101,6	137,6	109,167	140,9	104,6
118,6	75,3667	116,767	81,36667	119,6	77,7	117,733	79,4	119,1	76,5333	117,25	80,3833
116,167	83,6333	106,967	82,76667	116,567	89,9333	101,833	87,53333	116,367	86,7833	104,4	85,15
89,9667	70,6	89,4667	69,93333	90,6667	71,6	100,533	81,9	90,3167	71,1	95	75,9167
111,367	72,5333	117,267	78,36667	113,133	77,2333	119,233	72,9	112,25	74,8833	118,25	75,6333
82,6667	59,2	87,9667	60,4	82,7667	58,0667	91,7	58	82,7167	58,6333	89,8333	59,2
124,2	100,733	129,6	98,6	131,267	102,767	128	92,3	127,733	101,75	128,8	95,45
92,8667	78,5333	87,0333	83,1	91,6667	80,2333	96,3333	78,83334	92,2667	79,3833	91,6833	80,9667
106,267	74,9	109,267	80,73333	109,333	74,7667	113,9	77,1	107,8	74,8333	111,583	78,9167
118,167	90,2667	120,933	86,6	119,633	88,2	124,667	88,16666	118,9	89,2333	122,8	87,3833
126,267	80,6	126,9	86,53333	126,267	94,9333	128,433	81,76667	126,267	87,7667	127,667	84,15
97,7333	73,7333	112,2	81,86667	98,5333	78	99,8667	75	98,1333	75,8667	106,033	78,4333
122,067	88,9667	107,9	95,23333	117,9	102,5	124,667	90,9	119,983	95,7333	116,283	93,0667
138,767	101,1	127,533	97,6	131,967	98,6333	129,9	99,06667	135,367	99,8667	128,717	98,3333
126,267	77,6333	125,833	80,16666	135,3	81,1667	135,9	83,9	130,783	79,4	130,867	82,0333
135	65,8	134,133	67,16666	142,633	72,1667	138,9	71,43333	138,817	68,9833	136,517	69,3
118,433	77,4	113,733	80,03333	114,167	77,2333	110,467	78,53333	116,3	77,3167	112,1	79,2833
154,733	74,6333	144,633	75,2	135,333	89,9667	138,567	77,26667	145,033	82,3	141,6	76,2333
115,2	75	108,033	73,53333	108,4	77,4	107,133	78,1	111,8	76,2	107,583	75,8167
114,467	93,4333	124,9	86,53333	112,633	91	117,8	94,7	113,55	92,2167	121,35	90,6167
