# Supplementary material for: Kinetic gait analysis in healthy dogs and dogs with osteoarthritis: An evaluation of precision and overlap performance of a pressure-sensitive walkway and the use of symmetry indices
Source: PLoS One. 2020 Dec 15;15(12):e0243819. doi: 10.1371/journal.pone.0243819 (PMC7737891; doi:10.1371/journal.pone.0243819)
Supplement: S2 File — Fig 2 is based on symmetry indices calculated from measurements of maximum peak presure (A-D) and vertical impulse (E-H) of different legs in dogs with osteoarthritis. 2 different symmetry indices (SI1 (A, C, E, G) and SI2 (B, D, F, H)) are compared in 12 dogs with thoracic limb (A, B, E, F) and 9 dogs with pelvic limb lameness (C, D, G, H), respectively. (PDF) [file pone.0243819.s002.pdf]

S2 File. Comparison of different symmetry indices in dogs with osteoarthritis

Fig 2 is based on symmetry indices calculated from measurements of maximum peak presure (A-D) and vertical impuls (E-H) of different legs in dogs with osteoarthritis. 2 different symmetry indices (SI1 (A, C, E, G) and SI2 (B, D, F, H)) are compared in 12 dogs with thoracic limb (A, B, E, F) and 9 dogs with pelvic limb lameness (C, D, G, H), respectively.

Abbreviations:

RF	Right thoracic limb
RH	Right pelvic limb
LF	Left thoracic limb
LH	Left pelvic limb
SI	Symmetry index

2A
SI 1 of maximum peak pressure measured in dogs with thoracic limb lameness

LF/RH	RF/LH	LF/LH	RF/RH	LF/RF	LH/RH
1,211412	1,21632	1,31767	1,11824	1,08332	0,91936
1,013433	0,76113	0,94422	1,2241	1,24055	1,07331
1,425095	1,39443	1,45321	1,36745	1,04216	0,98065
1,484812	1,35179	1,43479	1,39892	1,0614	1,03487
1,638354	1,32642	1,72698	1,25835	1,30199	0,94868
1,213987	1,61802	1,34329	1,46227	0,83021	0,90374
1,558186	1,56644	1,61178	1,51436	1,02894	0,96675
1,314783	1,38655	1,39651	1,60863	1,00718	0,94148
1,29162	1,23884	1,34065	1,53272	1,08219	0,96342
1,375688	1,13463	1,21669	1,30713	1,07232	1,13068
1,224438	1,33583	1,1817	1,26535	0,88462	1,03617
1,396863	1,3448	1,27072	1,46587	0,94491	1,09927

2B
SI 2 of maximum peak pressure measured in dogs with thoracic limb lameness

LF/RH	RF/LH	LF/LH	RF/RH	LF/RF	LH/RH
0,0956	0,0976	0,13706	0,05582	0,03999	0,04201
0,00667	0,13563	0,02869	0,10076	0,10736	0,03536
0,17529	0,16473	0,18474	0,15521	0,02064	0,00977
0,19511	0,14959	0,17857	0,16629	0,02978	0,01713
0,24195	0,14031	0,26659	0,1144	0,13119	0,02633
0,09665	0,23606	0,1465	0,18774	0,09277	0,05056
0,2182	0,22071	0,23424	0,20457	0,01426	0,01691
0,13599	0,16197	0,16545	0,13248	0,00358	0,03014
0,12725	0,10668	0,14554	0,08823	0,03947	0,01863
0,15814	0,06307	0,09775	0,12392	0,0349	0,06133
0,1009	0,14377	0,08328	0,16113	0,06122	0,01776
0,16558	0,14705	0,11922	0,19299	0,02832	0,04729

2C

SI 1 of maximum peak pressure measured in dogs with pelvic limb lameness

LF/RH	RF/LH	LF/LH	RF/RH	LF/RF	LH/RH
1,644773	1,41142	1,44314	1,30541	1,02247	1,13972
1,214802	1,46755	1,16315	1,19353	0,79258	1,04441
1,906773	1,19673	1,74573	1,28291	1,45875	1,09225
1,303201	1,14065	1,17477	1,38415	1,02991	1,10932
1,345427	1,45148	1,33222	1,4783	0,91784	1,00991
1,643702	1,44594	1,55197	1,5314	1,07333	1,05911
1,796489	2,07555	1,75497	2,12465	0,84555	1,02366
0,8308443	1,50222	1,315	0,94914	0,87537	0,63182
1,228737	1,32109	1,3571	1,19613	1,02726	0,90541

2D

SI 2 of maximum peak pressure measured in dogs with pelvic limb lameness

LF/RH	RF/LH	LF/LH	RF/RH	LF/RF	LH/RH
0,24379	0,17061	0,18138	0,23331	0,01111	0,0653
0,09698	0,18948	0,07542	0,21033	0,11571	0,02172
0,31195	0,08956	0,27159	0,13312	0,18658	0,04409
0,13164	0,06571	0,08036	0,11713	0,01474	0,05183
0,14728	0,18417	0,14245	0,18893	0,04284	0,00493
0,24348	0,18232	0,21629	0,20992	0,03537	0,0287
0,28482	0,34971	0,27404	0,35993	0,08369	0,01169
0,09239	0,20071	0,13607	0,0261	0,06646	0,22563
0,10263	0,13834	0,1515	0,08931	0,01345	0,04964

2E

SI 1 of vertical impulse measured in dogs with thoracic limb lameness

LF/RH	RF/LH	LF/LH	RF/RH	LF/RF	LH/RH
1,46165	1,46726	1,50298	1,42692	1,02434	0,9725
1,877853	1,37174	1,86044	1,38458	1,35626	1,00936
1,780528	1,67524	1,73473	1,71947	1,03551	1,0264
1,019755	0,90521	1,02749	0,8984	1,13508	0,99247
1,693151	1,52306	1,62846	1,58356	1,0692	1,03973
1,572739	1,59012	1,60214	1,56094	1,00756	0,98165
1,97723	2,01996	2,07984	1,9203	1,02964	0,95066
2,005102	2,24958	1,96173	2,29932	0,87204	1,02211
1,324675	1,98931	1,40153	1,88023	0,70453	0,94517
1,811634	1,76818	1,79424	1,78532	1,01474	1,0097
1,771588	1,76439	1,70281	1,83566	0,9651	1,04039
1,713622	1,86227	1,58824	2,00929	0,85285	1,07895

2F

SI2 of vertical impulse measured in dogs with thoracic limb lameness

LF/RH	RF/LH	LF/LH	RF/RH	LF/RF	LH/RH
0,18754	0,18938	0,20095	0,17591	0,01202	0,01394
0,30504	0,15674	0,30081	0,16128	0,1512	0,00466
0,28071	0,2524	0,26867	0,26456	0,01744	0,01303
0,00978	0,04975	0,01356	0,05352	0,06327	0,00378
0,25738	0,20731	0,2391	0,22587	0,03344	0,01948
0,22262	0,22784	0,2314	0,21904	0,00376	0,00926
0,32823	0,33774	0,35062	0,31514	0,01461	0,02529
0,33447	0,38454	0,32472	0,39381	0,06835	0,01093
0,13966	0,33095	0,1672	0,30561	0,17335	0,02819
0,28867	0,2775	0,28424	0,28195	0,00732	0,00482
0,27839	0,27651	0,26003	0,2947	0,01776	0,0198
0,26298	0,30125	0,22727	0,33539	0,07942	0,03797

2G

SI 1 of vertical impulse measured in dogs with pelvic limb lameness

LF/RH	RF/LH	LF/LH	RF/RH	LF/RF	LH/RH
1,755494	1,7903	1,82311	1,7239	1,01833	0,96291
1,684146	1,75095	1,75477	1,68049	1,00218	0,95976
1,805556	1,37873	1,47551	1,68714	1,07019	1,22368
2,069909	2,29556	2,08576	2,27812	0,90861	0,9924
2,089527	1,85736	1,83804	2,11149	0,9896	1,13682
1,936688	1,99456	2,16515	1,78409	1,08553	0,89448
2,076923	2,04052	2,05673	2,06056	1,00794	1,00982
0,8425197	1,52226	1,28253	1	0,84252	0,65692
1,759657	1,92993	1,94774	1,74356	1,00923	0,90343

2H

SI 2 of vertical impulse measured in dogs with pelvic limb lameness

LF/RH	RF/LH	LF/LH	RF/RH	LF/RF	LH/RH
0,27418	0,28323	0,29156	0,26576	0,00908	0,01889
0,25488	0,27298	0,27399	0,25387	0,00109	0,02054
0,28713	0,15922	0,19208	0,25571	0,03391	0,10059
0,34851	0,39312	0,35186	0,38989	0,04789	0,00381
0,35265	0,30005	0,29529	0,35722	0,00523	0,06403
0,31896	0,33212	0,36812	0,28163	0,04101	0,0557
0,35	0,34222	0,34571	0,34652	0,00396	0,00489
0,08547	0,20706	0,12378	0	0,08547	0,20706
0,27527	0,31739	0,32151	0,27102	0,00459	0,05073
