# Supplementary material for: Kinetic gait analysis in healthy dogs and dogs with osteoarthritis: An evaluation of precision and overlap performance of a pressure-sensitive walkway and the use of symmetry indices
Source: PLoS One. 2020 Dec 15;15(12):e0243819. doi: 10.1371/journal.pone.0243819 (PMC7737891; doi:10.1371/journal.pone.0243819)
Supplement: S3 File — Fig 3 is based on left:right symmetry indices (SIs) of Maximum peak pressure (A-D) and Vertical impulse (E-H) measured in 21 dogs with osteoarthritis (OA) and 41 clinically healthy dogs, respectively. Dogs were divided in groups by the visual grade of lameness (0–5) of left (L) or right (R), thoracic (fore—F) and pelvic (hind—H) limb, respectively. SIs were calculated as simple ratios (SI (1), A,C,E,G) and as indices modified from Schnabl-Feichter et al., 2018 (SI (2), B,D,F,H). Using data from the clinically healthy dogs, parametric reference intervals were calculated for SI (1) as mean±2SD, whereas non-parametric reference intervals were calculated for SI (2) using either the 2.5% and 97.5% percentiles or the 95% percentile, as appropriate. (PDF) [file pone.0243819.s003.pdf]

S3 File. Symmetry indices in dogs with osteoarthritis comparing different grades and limbs of lameness

Fig 3 is based on left:right symmetry indices (SIs) of Maximum peak pressure (A-D) and Vertical impulse (E-H) measured in 21 dogs with osteoarthritis (OA) and 41 clinically healthy dogs, respectively.
Dogs were divided in groups by the visual grade of lameness (0-5) of left (L) or right (R), thoracic (F) and pelvic (H) limb, respectively.
SIs were calculated as simple ratios (SI (1), A,C,E,G) and as indices modified from Schnabl-Feichter et al., 2018 (SI (2), B,D,F,H).
Using data from the clinically healthy dogs, parametric reference intervals were calculated for SI (1) as mean±2SD, whereas non-parametric reference intervals were calculated for SI (2) using either the 2.5% and 97.5% percentiles or the 95% percentile, as appropriate.

**Abbreviations:**				
	RF	Right thoracic limb	RF 1, 2, 3	Grade 1, 2 or 3 lameness on right thoracic limb
	RH	Right pelvic limb	RH 1, 2	Grade 1 or 2 lameness on right pelvic limb
	LF	Left thoracic limb	LF 1, 2, 3	Grade 1, 2 or 3 lameness of left thoracic limb
	LH	Left pelvic limb	LH 1, 3	Grade 1 or 3 lameness of left pelvic limb
	SI	Symmetry index		

3A

SI 1 of maximum peak pressure comparing contralateral thoracic limbs (LF/RF)

Clinically healthy	RF1	RF2	RF3	LF1	LF2	LF3	RH1	RH2	LH1	LH3
1,063175	1,083321	1,24055	1,0614	0,88462	1,00718	0,94491	0,79258	1,02247	0,84555	1,07333
0,965873	1,042159				1,08219		1,45875	1,02991	1,02726	0,87537
1,017207	1,301986				1,07232			0,91784		
1,140644	0,83021									
0,9503696	1,028942									
1,082417										
1,01988										
0,9245852										
1,049574										
1,030071										

1,024247
1,039194
0,9128571
1,052956
1,048973
1,12788
1,021405
1,006149
1,171516
1,047942
1,029732
0,9657658
0,9772853
0,9765791
1,015778
1,114623
0,9507018
0,9492601
0,9207792
0,9917184
1,006362
0,9357231
0,9660941
0,968241
0,9890339
0,9254951
1,031819
1,051664
0,9993632
1,016848
1,037467

3E

SI 1 of vertical impulse comparing contralateral thoracic limbs (LF/RF)

Clinically healthy	RF1	RF2	RF3	LF1	LF2	LF3	RH1	RH2	LH1	LH3
1,063175	1,024341	1,35626	1,13508	0,9651	0,87204	0,85285	1,00218	1,01833	1,00794	1,08553
0,965873	1,035509				0,70453		1,07019	0,90861	1,00923	0,84252
1,017207	1,069204				1,01474			0,9896		
1,140644	1,007557									
0,9503696	1,029644									
1,082417										
1,01988										
0,9245852										
1,049574										
1,030071										
1,024247										
1,039194										
0,9128571										
1,052956										
1,048973										
1,12788										
1,021405										
1,006149										
1,171516										
1,047942										
1,029732										
0,9657658										
0,9772853										
0,9765791										
1,015778										
1,114623										
0,9507018										

0,9492601
0,9207792
0,9917184
1,006362
0,9357231
0,9660941
0,968241
0,9890339
0,9254951
1,031819
1,051664
0,9993632
1,016848
1,037467

3B

SI 2 of maximum peak pressure comparing contralateral thoracic limbs (LF/RF)

Clinically healthy	RF1	RF2	RF3	LF1	LF2	LF3	RH1	RH2	LH1	LH3
0,003231	0,03999	0,10736	0,02978	0,06122	0,00358	0,02832	0,11571	0,01111	0,08369	0,03537
0,01735971	0,02064				0,03947		0,18658	0,01474	0,01345	0,06646
0,008529997	0,13119				0,0349			0,04284		
0,0657018	0,09277									
0,02544667	0,01426									
0,03957747										
0,009842186										
0,03918495										
0,02418745										
0,01481287										
0,01197814										
0,01922054										
0,04555639										
0,02579505										
0,02390113										
0,06009746										
0,01058941										
0,003064921										
0,07898435										
0,02340967										
0,01464798										
0,01741521										
0,01148785										
0,01184919										
0,007827375										
0,05420504										
0,02527206										

0,02603037
0,04124408
0,004158004
0,003171152
0,03320562
0,01724531
0,01613571
0,005513258
0,03869388
0,01566027
0,02518144
0,000318492
0,008353511
0,01838879

3F

SI 2 of vertical impulse comparing contralateral thoracic limbs (LF/RF)

Clinically healthy	RF1	RF2	RF3	LF1	LF2	LF3	RH1	RH2	LH1	LH3
0,002722941	0,01202	0,1512	0,06327	0,01776	0,06835	0,07942	0,00109	0,00908	0,00396	0,04101
0,04854369	0,01744				0,17335		0,03391	0,04789	0,00459	0,08547
0,02662722	0,03344				0,00732			0,00523		
0,0114177	0,00376									
0,001810501	0,01461									
0,0207502										
0,01479915										
0,02058252										
0,000419992										
0,01833333										
0,01886792										
0,01561065										
0,007930786										

0,02416278
0,007668712
0,006661732
0,003312825
0,01248164
0,02349624
0,02009406
0,005134788
0,01459035
0,01836095
0,01318529
0,00038506
0,02842151
0,05906953
0,0211298
0,01217575
0,02111324
0,007525084
0,003308519
0,0176619
0,004016064
0,0174703
0,03762828
0,007493188
0,008257963
0,01166181
0,0142923
0,02173913

3C

SI 1 of maximum peak pressure comparing contralateral pelvic limbs (LH/ RH)

Clinically he	RF1	RF2	RF3	LF1	LF2	LF3	RH1	RH2	LH1	LH3
0,9830445	0,91936	1,07331	1,03487	1,03617	0,94148	1,09927	1,04441	1,13972	1,02366	1,05911
0,9726027	0,98065				0,96342		1,09225	1,10932	0,90541	0,63182
0,8967521	0,94868				1,13068			1,00991		
1,050662	0,90374									
1,02329	0,96675									
1,015007										
1,079331										
0,9787234										
0,778731										
1,133688										
1,07958										
1,005056										
0,9703028										
1,037421										
1,143394										
0,9280122										
0,8234157										
1,084889										
1,012534										
0,9774967										
1,001635										
1,071895										
0,9552522										
1,043658										
0,9521045										
1,019182										
0,9365532										

0,9900838
0,9904279
1,066003
0,9804446
1,017657
0,9482577
1,021171
1,042979
0,9672758
1,028653
1,015593
0,9678992
0,9954305
0,9751945

3G

SI 1 of vertical impulse comparing contralateral pelvic limbs (LH/RH)

Clinically healthy	RF1	RF2	RF3	LF1	LF2	LF3	RH1	RH2	LH1	LH3
0,9619182	0,9725	1,00936	0,99247	1,04039	1,02211	1,07895	0,95976	0,96291	1,00982	0,89448
1,002092	1,0264				0,94517		1,22368	0,9924	0,90343	0,65692
0,9243937	1,03973				1,0097			1,13682		
1,088509	0,98165									
0,9559083	0,95066									
0,9741936										
1,003252										
1,056575										
0,8321273										
1,091078										
1,068085										
0,895189										
1,124579										

1,08642
1
0,9025875
0,9586919
0,9796178
0,9617591
1,036337
1,004854
1,070545
0,9600639
1,01
0,9969419
1,039861
0,9796557
1,025424
1,048936
1,06187
0,9375
1,006221
0,9952904
0,9455587
1,09157
0,9142336
1,097765
1,042398
1,033103
1,015306
0,9723127

3D

SI 2 of maximum peak pressure comparing contralateral pelvic limbs (LH/RH)

Clinically he	RF1	RF2	RF3	LF1	LF2	LF3	RH1	RH2	LH1	LH3
0,008550221	0,04201	0,03536	0,01713	0,01776	0,03014	0,04729	0,02172	0,0653	0,01169	0,0287
0,01388889	0,00977				0,01863		0,04409	0,05183	0,04964	0,22563
0,05443403	0,02633				0,06133			0,00493		
0,02470522	0,05056									
0,01151105	0,01691									
0,007447529										
0,03815224										
0,01075269										
0,1243971										
0,06265602										
0,03826745										
0,002521653										
0,01507241										
0,01836707										
0,06690023										
0,03733782										
0,0968426										
0,04071648										
0,006227867										
0,01137968										
0,00081666										
0,03470032										
0,02288596										
0,02136286										
0,02453532										
0,009499806										
0,03276272										

0,004982837
0,004809052
0,03194726
0,009874233
0,008751139
0,02655827
0,01047466
0,02103732
0,01663426
0,01412429
0,007736293
0,0163122
0,002289984
0,01255854

3H

SI 2 of vertical impulse comparing contralateral pelvic limbs (LH/RH)

Clinically healthy	RF1	RF2	RF3	LF1	LF2	LF3	RH1	RH2	LH1	LH3
0,0194105	0,01394	0,00466	0,00378	0,0198	0,01093	0,03797	0,02054	0,01889	0,00489	0,0557
0,001044932	0,01303				0,02819		0,10059	0,00381	0,05073	0,20706
0,03928836	0,01948				0,00482			0,06403		
0,04237918	0,00926									
0,02254283	0,02529									
0,01307189										
0,001623377										
0,02750929										
0,09162717										
0,04355556										
0,03292181										
0,05530372										
0,05863708										

0,04142012

0

0,0512

0,02108963

0,01029601

0,01949318

0,0178444

0,002421308

0,03407053

0,0203749

0,004975124

0,001531394

0,01954121

0,01027668

0,0125523

0,0238837

0,03000698

0,03225806

0,003100775

0,002360346

0,02798233

0,0437804

0,04480458

0,04660453

0,02075877

0,01628223

0,007594937

0,01403799
