# Supplementary material for: Kinetic gait analysis in healthy dogs and dogs with osteoarthritis: An evaluation of precision and overlap performance of a pressure-sensitive walkway and the use of symmetry indices
Source: PLoS One. 2020 Dec 15;15(12):e0243819. doi: 10.1371/journal.pone.0243819 (PMC7737891; doi:10.1371/journal.pone.0243819)
Supplement: S4 File — The calculated means and standard deviation listed in Table 1 are based on 6 walkway measurements of each limb of 41 clinically healthy dogs. Temporal characteristics and measured vertical ground reaction forces are listed in the present file. (PDF) [file pone.0243819.s004.pdf]

S4 File. Temporal characteristics and vertical ground reaction forces measured in clinically healthy dogs

The calculated means and standard deviation listed in Table 1 are based on 6 walkway measurements of each limb of 41 clinically healthy dogs. Temporal characteristics and measured vertical ground reaction forces are listed in the present file.

**Abbreviations**:	RF	Measured ground reaction forces in 6 recordings of right thoracic limb
	RH	Measured ground reaction forces in 6 recordings of right pelvic limb
	LF	Measured ground reaction forces in 6 recordings of left thoracic limb
	LH	Measured ground reaction forces in 6 recordings of left pelvic limb

Stance time (sec)

**LF**						**RF**					
0,47	0,45	0,45	0,47	0,5	0,51	0,45	0,44	0,47	0,45	0,49	0,49
0,48	0,48	0,49	0,51	0,49	0,49	0,47	0,47	0,48	0,48	0,48	0,48
0,49	0,47	0,45	0,46	0,47	0,48	0,51	0,48	0,47	0,49	0,48	0,49
0,54	0,55	0,54	0,53	0,55	0,58	0,54	0,56	0,62	0,52	0,54	0,56
0,49	0,5	0,48	0,53	0,49	0,51	0,49	0,49	0,47	0,5	0,46	0,51
0,58	0,63	0,63	0,6	0,55	0,59	0,58	0,64	0,64	0,59	0,58	0,59
0,63	0,6	0,62	0,56	0,61	0,61	0,63	0,59	0,62	0,57	0,58	0,61
0,45	0,43	0,45	0,45	0,47	0,47	0,44	0,42	0,47	0,46	0,46	0,48
0,47	0,49	0,45	0,52	0,5	0,51	0,44	0,5	0,48	0,5	0,49	0,5
0,49	0,52	0,55	0,53	0,56	0,53	0,49	0,52	0,52	0,52	0,57	0,53
0,38	0,4	0,39	0,39	0,41	0,42	0,37	0,41	0,39	0,4	0,39	0,41
0,54	0,55	0,52	0,52	0,52	0,5	0,54	0,55	0,53	0,53	0,52	0,49
0,54	0,53	0,51	0,52	0,51	0,52	0,53	0,53	0,5	0,53	0,52	0,53
0,49	0,47	0,49	0,46	0,48	0,51	0,51	0,5	0,51	0,48	0,49	0,54
0,52	0,51	0,54	0,52	0,61	0,55	0,53	0,52	0,56	0,53	0,6	0,54
0,51	0,54	0,52	0,55	0,53	0,54	0,5	0,53	0,51	0,53	0,5	0,53
0,56	0,55	0,56	0,57	0,54	0,55	0,56	0,54	0,58	0,52	0,52	0,56
0,42	0,4	0,42	0,39	0,42	0,44	0,4	0,41	0,4	0,39	0,42	0,45
0,47	0,48	0,46	0,5	0,48	0,49	0,49	0,48	0,47	0,49	0,51	0,48

0,54	0,57	0,55	0,59	0,58	0,59	0,54	0,58	0,56	0,58	0,61	0,62
0,47	0,48	0,47	0,47	0,49	0,46	0,47	0,47	0,45	0,47	0,49	0,47
0,53	0,55	0,54	0,54	0,53	0,52	0,55	0,55	0,56	0,53	0,52	0,52
0,46	0,49	0,51	0,48	0,48	0,47	0,47	0,5	0,49	0,49	0,51	0,44
0,39	0,4	0,35	0,41	0,39	0,41	0,4	0,4	0,35	0,43	0,4	0,41
0,43	0,45	0,43	0,46	0,45	0,46	0,43	0,46	0,42	0,47	0,47	0,49
0,41	0,36	0,39	0,39	0,35	0,38	0,42	0,38	0,4	0,36	0,38	0,39
0,49	0,5	0,51	0,53	0,5	0,51	0,47	0,49	0,51	0,52	0,5	0,51
0,42	0,48	0,45	0,49	0,53	0,5	0,4	0,45	0,45	0,51	0,52	0,49
0,53	0,5	0,54	0,55	0,52	0,53	0,54	0,51	0,51	0,55	0,53	0,55
0,58	0,59	0,59	0,57	0,58	0,6	0,59	0,6	0,6	0,55	0,58	0,63
0,45	0,43	0,44	0,48	0,47	0,45	0,45	0,45	0,45	0,49	0,49	0,46
0,52	0,5	0,53	0,54	0,52	0,52	0,51	0,51	0,53	0,56	0,53	0,54
0,45	0,47	0,49	0,54	0,55	0,54	0,46	0,47	0,48	0,54	0,56	0,57
0,5	0,51	0,48	0,49	0,51	0,5	0,5	0,5	0,48	0,47	0,5	0,51
0,36	0,36	0,38	0,41	0,4	0,37	0,37	0,37	0,37	0,39	0,37	0,35
0,48	0,47	0,46	0,49	0,46	0,47	0,48	0,46	0,48	0,5	0,47	0,47
0,57	0,52	0,57	0,53	0,59	0,58	0,57	0,51	0,56	0,53	0,59	0,56
0,38	0,43	0,41	0,43	0,44	0,44	0,39	0,42	0,41	0,43	0,43	0,45
0,53	0,57	0,53	0,54	0,56	0,55	0,54	0,56	0,49	0,54	0,59	0,55
0,53	0,52	0,56	0,57	0,54	0,52	0,54	0,52	0,55	0,58	0,54	0,51
0,41	0,41	0,41	0,38	0,38	0,39	0,37	0,36	0,38	0,37	0,35	0,37

**LH**						**RH**					
0,42	0,43	0,44	0,44	0,47	0,48	0,43	0,42	0,41	0,44	0,49	0,48
0,45	0,44	0,46	0,49	0,45	0,45	0,45	0,43	0,43	0,45	0,44	0,47
0,43	0,4	0,39	0,42	0,41	0,42	0,46	0,42	0,44	0,45	0,43	0,43
0,52	0,54	0,56	0,5	0,52	0,5	0,53	0,56	0,57	0,48	0,5	0,53
0,47	0,48	0,46	0,47	0,43	0,47	0,46	0,48	0,47	0,47	0,41	0,46
0,53	0,58	0,59	0,56	0,52	0,56	0,54	0,61	0,6	0,58	0,54	0,57
0,64	0,61	0,65	0,54	0,58	0,59	0,59	0,57	0,59	0,53	0,57	0,57
0,44	0,41	0,43	0,43	0,43	0,43	0,43	0,4	0,45	0,43	0,43	0,43

0,44	0,47	0,46	0,46	0,46	0,45	0,42	0,44	0,45	0,46	0,44	0,45
0,48	0,5	0,51	0,52	0,53	0,49	0,48	0,5	0,51	0,52	0,54	0,51
0,34	0,35	0,37	0,35	0,37	0,39	0,33	0,37	0,37	0,34	0,37	0,39
0,49	0,51	0,5	0,49	0,49	0,47	0,53	0,53	0,49	0,49	0,5	0,49
0,51	0,49	0,49	0,49	0,5	0,46	0,49	0,49	0,49	0,48	0,49	0,48
0,49	0,47	0,47	0,46	0,45	0,48	0,49	0,49	0,5	0,47	0,47	0,51
0,49	0,48	0,53	0,48	0,55	0,52	0,51	0,5	0,54	0,51	0,56	0,53
0,48	0,53	0,51	0,55	0,51	0,52	0,49	0,53	0,5	0,52	0,5	0,51
0,55	0,51	0,55	0,51	0,5	0,5	0,54	0,51	0,54	0,52	0,5	0,52
0,37	0,36	0,35	0,31	0,37	0,41	0,35	0,34	0,36	0,33	0,36	0,39
0,46	0,45	0,46	0,51	0,5	0,48	0,48	0,49	0,44	0,49	0,49	0,5
0,52	0,55	0,52	0,56	0,56	0,57	0,51	0,54	0,53	0,57	0,59	0,59
0,42	0,45	0,44	0,44	0,45	0,43	0,42	0,43	0,43	0,43	0,45	0,43
0,49	0,51	0,5	0,48	0,49	0,47	0,55	0,53	0,53	0,51	0,52	0,51
0,44	0,39	0,49	0,39	0,46	0,49	0,44	0,42	0,48	0,39	0,49	0,48
0,36	0,36	0,31	0,37	0,35	0,36	0,34	0,35	0,3	0,39	0,37	0,38
0,39	0,41	0,37	0,42	0,41	0,42	0,39	0,42	0,39	0,44	0,43	0,43
0,37	0,32	0,33	0,33	0,31	0,36	0,35	0,33	0,34	0,32	0,31	0,39
0,49	0,42	0,51	0,48	0,47	0,47	0,46	0,53	0,51	0,5	0,48	0,5
0,36	0,43	0,39	0,45	0,45	0,44	0,36	0,44	0,4	0,46	0,49	0,45
0,49	0,45	0,48	0,53	0,49	0,5	0,53	0,48	0,48	0,48	0,53	0,51
0,54	0,56	0,57	0,51	0,53	0,55	0,53	0,55	0,55	0,54	0,53	0,56
0,41	0,39	0,39	0,45	0,45	0,41	0,43	0,42	0,45	0,47	0,45	0,42
0,48	0,46	0,36	0,54	0,41	0,51	0,5	0,49	0,43	0,55	0,4	0,51
0,44	0,45	0,47	0,5	0,5	0,5	0,42	0,43	0,46	0,49	0,51	0,5
0,48	0,49	0,47	0,47	0,46	0,49	0,49	0,49	0,45	0,46	0,5	0,47
0,35	0,37	0,36	0,39	0,37	0,35	0,35	0,38	0,37	0,37	0,37	0,35
0,44	0,43	0,45	0,45	0,45	0,42	0,44	0,44	0,45	0,46	0,44	0,46
0,53	0,49	0,55	0,5	0,62	0,54	0,55	0,51	0,57	0,5	0,58	0,57
0,37	0,39	0,41	0,44	0,43	0,43	0,39	0,43	0,4	0,43	0,42	0,47
0,53	0,54	0,51	0,53	0,56	0,57	0,53	0,58	0,5	0,55	0,59	0,54
0,5	0,49	0,55	0,57	0,53	0,51	0,51	0,5	0,45	0,55	0,52	0,51

0,36	0,38	0,35	0,35	0,33	0,35	0,35	0,36	0,36	0,35	0,32	0,34

Swing time (sec)

LF						RF					
0,26	0,28	0,26	0,28	0,27	0,28	0,28	0,27	0,26	0,28	0,29	0,29
0,29	0,3	0,3	0,32	0,29	0,3	0,3	0,3	0,3	0,36	0,3	0,3
0,29	0,26	0,28	0,28	0,27	0,26	0,26	0,25	0,25	0,25	0,26	0,26
0,28	0,32	0,37	0,3	0,26	0,3	0,3	0,3	0,3	0,32	0,3	0,34
0,26	0,28	0,27	0,28	0,28	0,28	0,28	0,28	0,28	0,3	0,28	0,28
0,34	0,32	0,34	0,34	0,34	0,32	0,34	0,32	0,34	0,36	0,32	0,34
0,36	0,32	0,36	0,3	0,32	0,34	0,35	0,34	0,34	0,31	0,32	0,32
0,29	0,27	0,3	0,29	0,27	0,28	0,3	0,28	0,28	0,28	0,28	0,28
0,29	0,3	0,3	0,3	0,3	0,3	0,32	0,3	0,27	0,3	0,3	0,3
0,26	0,29	0,28	0,29	0,28	0,3	0,28	0,28	0,3	0,3	0,3	0,29
0,21	0,24	0,23	0,21	0,24	0,24	0,24	0,24	0,24	0,22	0,25	0,25
0,3	0,32	0,3	0,28	0,28	0,3	0,32	0,32	0,3	0,28	0,32	0,32
0,3	0,32	0,31	0,3	0,3	0,3	0,3	0,32	0,32	0,28	0,3	0,29
0,34	0,34	0,34	0,34	0,34	0,34	0,32	0,3	0,32	0,33	0,32	0,3
0,3	0,29	0,32	0,3	0,32	0,31	0,28	0,28	0,3	0,29	0,3	0,32
0,24	0,26	0,28	0,27	0,27	0,28	0,27	0,28	0,27	0,3	0,3	0,29
0,34	0,34	0,34	0,34	0,34	0,36	0,36	0,38	0,38	0,36	0,38	0,34
0,26	0,26	0,24	0,23	0,25	0,26	0,28	0,26	0,27	0,23	0,26	0,26
0,32	0,32	0,3	0,32	0,34	0,31	0,3	0,31	0,28	0,3	0,3	0,3
0,3	0,32	0,32	0,31	0,34	0,32	0,3	0,3	0,32	0,32	0,34	0,32
0,28	0,28	0,28	0,28	0,28	0,29	0,29	0,29	0,28	0,28	0,29	0,28
0,32	0,31	0,32	0,3	0,3	0,3	0,29	0,32	0,3	0,3	0,3	0,3
0,26	0,28	0,3	0,3	0,3	0,29	0,27	0,28	0,3	0,28	0,28	0,32
0,26	0,26	0,24	0,28	0,26	0,26	0,25	0,26	0,26	0,27	0,27	0,28
0,26	0,27	0,25	0,29	0,29	0,28	0,24	0,26	0,28	0,25	0,26	0,25
0,23	0,24	0,24	0,22	0,25	0,26	0,23	0,22	0,24	0,24	0,25	0,26
0,28	0,29	0,3	0,28	0,3	0,3	0,29	0,28	0,3	0,3	0,28	0,28
0,26	0,26	0,26	0,3	0,3	0,28	0,36	0,31	0,26	0,3	0,3	0,3

0,29	0,3	0,28	0,3	0,32	0,32	0,28	0,28	0,32	0,28	0,3	0,3
0,32	0,33	0,35	0,32	0,34	0,34	0,32	0,32	0,32	0,34	0,34	0,32
0,29	0,28	0,26	0,28	0,3	0,28	0,28	0,26	0,26	0,28	0,29	0,27
0,32	0,32	0,32	0,32	0,32	0,32	0,32	0,31	0,32	0,33	0,32	0,32
0,28	0,26	0,26	0,26	0,28	0,29	0,26	0,26	0,26	0,27	0,26	0,26
0,3	0,3	0,28	0,26	0,3	0,28	0,29	0,31	0,28	0,3	0,3	0,29
0,28	0,29	0,24	0,26	0,25	0,27	0,26	0,28	0,27	0,29	0,28	0,28
0,32	0,31	0,3	0,32	0,32	0,3	0,34	0,3	0,3	0,3	0,31	0,31
0,32	0,32	0,36	0,32	0,34	0,32	0,33	0,34	0,34	0,32	0,35	0,32
0,27	0,28	0,27	0,28	0,3	0,28	0,26	0,29	0,26	0,28	0,29	0,29
0,36	0,36	0,34	0,36	0,4	0,36	0,36	0,34	0,36	0,36	0,36	0,34
0,28	0,28	0,28	0,3	0,29	0,3	0,27	0,3	0,3	0,3	0,3	0,29
0,22	0,23	0,23	0,24	0,23	0,23	0,27	0,27	0,25	0,26	0,25	0,26

**LH**						**RH**					
0,3	0,27	0,29	0,28	0,32	0,32	0,28	0,28	0,3	0,3	0,3	0,31
0,32	0,32	0,34	0,35	0,35	0,34	0,34	0,35	0,38	0,36	0,36	0,34
0,33	0,32	0,32	0,32	0,34	0,32	0,31	0,3	0,28	0,3	0,31	0,3
0,34	0,34	0,36	0,34	0,32	0,32	0,32	0,32	0,34	0,32	0,34	0,32
0,3	0,32	0,32	0,3	0,3	0,34	0,32	0,34	0,32	0,31	0,31	0,32
0,38	0,4	0,4	0,4	0,38	0,4	0,38	0,36	0,4	0,38	0,34	0,38
0,38	0,38	0,36	0,32	0,36	0,36	0,4	0,36	0,38	0,34	0,36	0,38
0,3	0,29	0,34	0,32	0,34	0,32	0,32	0,28	0,32	0,32	0,33	0,3
0,32	0,34	0,34	0,34	0,34	0,34	0,34	0,34	0,34	0,33	0,34	0,34
0,3	0,32	0,3	0,32	0,34	0,32	0,31	0,32	0,32	0,32	0,33	0,31
0,28	0,28	0,28	0,28	0,28	0,3	0,27	0,27	0,28	0,28	0,28	0,29
0,38	0,36	0,36	0,34	0,36	0,34	0,32	0,35	0,34	0,35	0,33	0,32
0,32	0,35	0,35	0,34	0,34	0,36	0,36	0,34	0,34	0,34	0,34	0,35
0,38	0,34	0,36	0,34	0,36	0,38	0,36	0,3	0,34	0,34	0,32	0,34
0,34	0,33	0,34	0,36	0,36	0,35	0,32	0,32	0,33	0,32	0,36	0,34
0,32	0,32	0,3	0,31	0,31	0,32	0,3	0,31	0,3	0,34	0,3	0,32

0,4	0,4	0,42	0,4	0,38	0,4	0,4	0,38	0,4	0,38	0,38	0,38
0,31	0,3	0,32	0,3	0,31	0,31	0,33	0,31	0,32	0,29	0,32	0,36
0,34	0,32	0,28	0,33	0,34	0,32	0,32	0,32	0,3	0,34	0,34	0,34
0,32	0,36	0,36	0,34	0,38	0,38	0,34	0,36	0,34	0,4	0,37	0,36
0,33	0,32	0,32	0,3	0,32	0,34	0,35	0,34	0,35	0,33	0,34	0,32
0,36	0,36	0,36	0,34	0,34	0,34	0,32	0,36	0,33	0,32	0,32	0,31
0,28	0,27	0,3	0,23	0,36	0,3	0,3	0,21	0,32	0,23	0,32	0,3
0,3	0,32	0,3	0,33	0,32	0,32	0,31	0,32	0,31	0,32	0,31	0,3
0,3	0,3	0,3	0,32	0,33	0,32	0,3	0,31	0,29	0,3	0,3	0,3
0,26	0,27	0,29	0,26	0,25	0,3	0,28	0,27	0,27	0,28	0,26	0,3
0,26	0,34	0,33	0,34	0,33	0,34	0,31	0,27	0,32	0,32	0,3	0,31
0,32	0,32	0,32	0,34	0,37	0,32	0,3	0,3	0,3	0,33	0,3	0,32
0,34	0,34	0,34	0,35	0,33	0,34	0,32	0,34	0,3	0,34	0,36	0,32
0,38	0,36	0,36	0,4	0,4	0,4	0,38	0,38	0,38	0,38	0,38	0,4
0,34	0,3	0,32	0,32	0,32	0,31	0,34	0,29	0,3	0,31	0,31	0,3
0,34	0,34	0,2	0,38	0,28	0,36	0,32	0,3	0,24	0,36	0,36	0,36
0,31	0,3	0,3	0,31	0,34	0,33	0,3	0,3	0,3	0,34	0,31	0,32
0,32	0,34	0,31	0,3	0,36	0,32	0,32	0,33	0,32	0,31	0,34	0,34
0,29	0,29	0,29	0,3	0,3	0,3	0,28	0,26	0,28	0,31	0,3	0,28
0,36	0,38	0,36	0,36	0,34	0,36	0,34	0,36	0,34	0,34	0,34	0,32
0,38	0,36	0,38	0,34	0,38	0,36	0,34	0,3	0,36	0,32	0,38	0,36
0,28	0,32	0,29	0,3	0,31	0,33	0,28	0,29	0,3	0,3	0,32	0,31
0,34	0,36	0,34	0,36	0,36	0,36	0,36	0,36	0,34	0,34	0,34	0,38
0,34	0,34	0,3	0,38	0,32	0,34	0,34	0,32	0,36	0,36	0,32	0,34
0,26	0,27	0,28	0,28	0,27	0,27	0,28	0,28	0,27	0,27	0,27	0,29

Stride time (sec)

LF						RF					
0,74	0,7	0,72	0,72	0,77	0,78	0,73	0,72	0,74	0,74	0,77	0,78
0,76	0,78	0,77	0,84	0,78	0,79	0,76	0,77	0,76	0,84	0,78	0,76
0,78	0,76	0,72	0,74	0,74	0,72	0,78	0,75	0,72	0,75	0,72	0,75
0,8	0,86	0,91	0,84	0,82	0,92	0,84	0,85	0,96	0,84	0,86	0,9

0,76	0,77	0,74	0,82	0,8	0,8	0,78	0,76	0,74	0,82	0,76	0,8
0,94	0,94	0,96	0,94	0,9	0,9	0,92	0,96	0,98	0,96	0,9	0,94
0,96	0,92	0,96	0,86	0,92	0,94	0,97	0,94	0,94	0,89	0,88	0,92
0,72	0,7	0,73	0,73	0,73	0,75	0,72	0,72	0,72	0,74	0,72	0,76
0,75	0,8	0,74	0,84	0,8	0,8	0,76	0,82	0,74	0,8	0,8	0,8
0,76	0,81	0,82	0,8	0,84	0,84	0,76	0,8	0,84	0,78	0,84	0,82
0,58	0,65	0,6	0,58	0,64	0,65	0,63	0,65	0,61	0,6	0,64	0,66
0,84	0,84	0,8	0,8	0,78	0,79	0,84	0,86	0,82	0,8	0,84	0,78
0,86	0,85	0,82	0,82	0,8	0,82	0,82	0,84	0,82	0,8	0,81	0,82
0,82	0,82	0,84	0,8	0,82	0,83	0,82	0,82	0,82	0,8	0,8	0,84
0,8	0,8	0,86	0,82	0,92	0,85	0,8	0,78	0,85	0,82	0,88	0,86
0,74	0,78	0,78	0,8	0,79	0,8	0,77	0,8	0,78	0,82	0,8	0,8
0,88	0,88	0,88	0,94	0,88	0,92	0,9	0,92	0,94	0,88	0,92	0,9
0,67	0,67	0,64	0,61	0,66	0,69	0,68	0,68	0,66	0,62	0,67	0,69
0,78	0,82	0,76	0,8	0,8	0,79	0,78	0,8	0,76	0,8	0,8	0,76
0,84	0,88	0,86	0,88	0,92	0,9	0,84	0,86	0,88	0,88	0,92	0,92
0,74	0,76	0,73	0,75	0,78	0,74	0,75	0,76	0,72	0,76	0,78	0,74
0,84	0,84	0,86	0,84	0,84	0,84	0,84	0,84	0,86	0,84	0,84	0,82
0,72	0,76	0,81	0,78	0,77	0,75	0,75	0,76	0,78	0,78	0,74	0,76
0,66	0,66	0,58	0,68	0,64	0,66	0,65	0,66	0,59	0,69	0,66	0,68
0,69	0,73	0,69	0,74	0,73	0,75	0,66	0,72	0,72	0,71	0,72	0,74
0,64	0,61	0,64	0,63	0,61	0,64	0,65	0,6	0,66	0,62	0,65	0,65
0,78	0,77	0,8	0,81	0,8	0,81	0,76	0,77	0,8	0,82	0,78	0,8
0,69	0,72	0,74	0,79	0,82	0,76	0,78	0,75	0,72	0,8	0,82	0,78
0,82	0,8	0,84	0,83	0,84	0,84	0,82	0,78	0,84	0,82	0,82	0,86
0,9	0,92	0,93	0,88	0,9	0,93	0,9	0,92	0,92	0,88	0,92	0,94
0,74	0,71	0,68	0,76	0,78	0,72	0,72	0,72	0,71	0,76	0,79	0,73
0,84	0,82	0,86	0,84	0,82	0,82	0,84	0,82	0,86	0,88	0,84	0,84
0,72	0,73	0,74	0,8	0,86	0,84	0,72	0,74	0,72	0,81	0,82	0,84
0,78	0,78	0,76	0,74	0,79	0,78	0,78	0,8	0,74	0,77	0,78	0,79
0,64	0,64	0,62	0,67	0,65	0,64	0,63	0,65	0,64	0,68	0,66	0,63
0,81	0,77	0,76	0,8	0,76	0,78	0,82	0,74	0,78	0,8	0,78	0,77

0,88	0,84	0,9	0,84	0,92	0,88	0,89	0,86	0,9	0,84	0,92	0,86
0,65	0,7	0,69	0,7	0,74	0,72	0,66	0,7	0,68	0,7	0,72	0,72
0,88	0,9	0,88	0,92	0,94	0,9	0,92	0,88	0,86	0,9	0,96	0,9
0,8	0,78	0,84	0,84	0,82	0,82	0,8	0,8	0,86	0,85	0,84	0,79
0,63	0,64	0,63	0,63	0,62	0,62	0,65	0,63	0,63	0,63	0,61	0,63

**LH**						**RH**					
0,72	0,7	0,72	0,74	0,8	0,8	0,71	0,7	0,72	0,74	0,78	0,78
0,76	0,76	0,78	0,82	0,79	0,78	0,78	0,78	0,82	0,8	0,8	0,8
0,77	0,73	0,72	0,75	0,74	0,74	0,78	0,74	0,73	0,74	0,75	0,74
0,88	0,88	0,94	0,86	0,86	0,84	0,84	0,86	0,92	0,82	0,84	0,88
0,78	0,79	0,76	0,78	0,74	0,82	0,78	0,82	0,78	0,79	0,73	0,8
0,9	0,98	1	0,96	0,9	0,94	0,92	0,96	1	0,94	0,88	0,94
1	0,94	0,97	0,86	0,94	0,96	0,98	0,9	0,96	0,9	0,9	0,94
0,73	0,69	0,77	0,76	0,76	0,76	0,74	0,68	0,76	0,76	0,76	0,74
0,76	0,82	0,78	0,8	0,82	0,8	0,72	0,8	0,77	0,8	0,78	0,8
0,76	0,82	0,82	0,83	0,86	0,82	0,77	0,82	0,82	0,84	0,87	0,82
0,61	0,64	0,63	0,62	0,65	0,68	0,58	0,65	0,64	0,61	0,65	0,68
0,86	0,88	0,84	0,82	0,84	0,82	0,83	0,88	0,8	0,83	0,83	0,8
0,82	0,85	0,83	0,83	0,84	0,8	0,85	0,84	0,82	0,82	0,82	0,82
0,86	0,81	0,82	0,8	0,8	0,86	0,84	0,78	0,84	0,8	0,78	0,84
0,82	0,8	0,84	0,84	0,91	0,86	0,82	0,82	0,87	0,83	0,92	0,86
0,78	0,84	0,82	0,85	0,8	0,84	0,78	0,83	0,8	0,84	0,8	0,83
0,94	0,9	0,96	0,92	0,88	0,9	0,92	0,88	0,92	0,9	0,9	0,9
0,68	0,65	0,67	0,6	0,67	0,71	0,68	0,65	0,68	0,61	0,66	0,74
0,8	0,78	0,76	0,82	0,84	0,78	0,8	0,82	0,74	0,82	0,84	0,8
0,84	0,9	0,88	0,86	0,92	0,92	0,86	0,88	0,86	0,94	0,93	0,93
0,75	0,76	0,74	0,76	0,78	0,75	0,76	0,75	0,75	0,76	0,79	0,75
0,84	0,84	0,84	0,82	0,82	0,8	0,84	0,88	0,85	0,84	0,84	0,82
0,74	0,6	0,8	0,58	0,8	0,77	0,74	0,68	0,8	0,69	0,82	0,76
0,65	0,67	0,59	0,7	0,66	0,68	0,65	0,66	0,6	0,7	0,67	0,67

0,69	0,72	0,68	0,74	0,75	0,75	0,7	0,74	0,69	0,74	0,74	0,74
0,62	0,6	0,64	0,62	0,57	0,64	0,64	0,61	0,62	0,64	0,6	0,65
0,76	0,68	0,82	0,82	0,79	0,82	0,74	0,78	0,8	0,81	0,78	0,82
0,7	0,74	0,71	0,8	0,81	0,8	0,66	0,72	0,7	0,79	0,74	0,78
0,84	0,8	0,82	0,85	0,82	0,84	0,86	0,82	0,78	0,82	0,86	0,82
0,92	0,92	0,92	0,9	0,92	0,96	0,92	0,92	0,92	0,92	0,92	0,98
0,75	0,68	0,7	0,77	0,77	0,72	0,78	0,71	0,71	0,77	0,76	0,72
0,82	0,78	0,44	0,92	0,65	0,86	0,82	0,8	0,63	0,9	0,64	0,86
0,73	0,73	0,75	0,81	0,82	0,83	0,7	0,72	0,74	0,82	0,82	0,82
0,8	0,84	0,78	0,78	0,82	0,8	0,81	0,82	0,78	0,77	0,82	0,8
0,63	0,65	0,65	0,7	0,66	0,66	0,62	0,64	0,64	0,69	0,67	0,66
0,8	0,8	0,8	0,8	0,78	0,76	0,78	0,79	0,78	0,8	0,78	0,78
0,9	0,86	0,98	0,82	0,96	0,86	0,88	0,82	0,92	0,82	0,94	0,9
0,64	0,7	0,7	0,72	0,74	0,74	0,66	0,71	0,68	0,72	0,74	0,76
0,88	0,88	0,86	0,88	0,92	0,94	0,9	0,92	0,84	0,9	0,94	0,92
0,82	0,82	0,83	0,92	0,84	0,82	0,83	0,82	0,7	0,9	0,82	0,84
0,62	0,64	0,65	0,63	0,62	0,61	0,63	0,64	0,63	0,62	0,6	0,63

Stride length (cm)

**LF**						**RF**					
78,2	70,1	76,2	74,2	73,2	75,2	75,7	70,6	76,2	74,2	72,6	74,7
76,2	79,2	80,3	82,3	81,3	79,2	76,2	78,2	79,2	82,3	79,2	76,2
71,1	70,1	74,2	73,2	75,2	75,2	70,1	70,6	74,2	72,6	74,2	74,2
88,4	81,3	86,9	92,5	89,4	90,4	86,4	84,3	90,4	90,4	86,4	86,4
79,2	80,3	79,8	79,2	82,3	78,2	78,2	79,2	80,3	80,3	86,4	77,2
94,5	91,4	92,5	93,5	94,5	88,4	94,5	92,5	90,4	94,5	93,5	89,4
85,3	87,4	87,4	85,3	94,5	86,4	87,4	88,4	86,4	85,3	92,5	88,4
75,7	74,2	74,2	75,2	76,2	73,7	73,2	73,2	75,2	76,2	76,2	73,2
79,2	78,7	80,3	79,2	79,2	78,2	82,3	79,2	79,2	79,2	78,7	78,7
80,3	81,3	77,2	78,7	76,2	78,2	81,3	80,3	76,7	77,2	77,2	77,7
62	59,9	62,5	62,5	58,9	61	60,5	60,5	62	61,5	58,9	60,6
80,3	81,3	79,2	81,3	81,3	79,8	80,3	77,7	78,7	81,3	79,2	77,2

79,2	81,3	81,3	75,7	81,3	79,2	79,2	82,3	83,3	75,2	80,8	79,8
78,2	82,3	81,3	80,3	83,3	80,8	78,7	82,3	78,7	79,8	81,8	81,3
80,3	78,7	79,2	78,2	79,2	81,3	79,8	80,3	79,2	78,7	81,3	80,3
77,2	73,2	75,2	73,2	76,7	75,2	76,7	73,2	73,7	75,2	78,2	75,2
90,4	93,5	88,4	85,3	88,4	89,4	91,4	92,5	88,4	88,4	92,5	91,4
71,6	69,6	70,1	66,5	67,1	68,1	71,1	68,1	71,6	66	69,1	67,6
84,8	83,3	83,3	87,4	83,3	86,9	88,4	83,8	82,3	86,4	83,3	87,4
85,3	86,4	85,3	83,8	85,3	84,3	88,4	86,4	85,3	84,3	85,3	82,3
73,2	73,7	72,6	71,1	69,1	73,7	72,6	73,7	73,7	68,1	70,6	72,1
81,3	80,3	82,3	83,3	78,2	82,3	80,3	80,3	80,3	84,3	81,3	82,3
79,2	86,4	79,8	82,3	83,8	78,2	79,8	83,8	80,3	83,3	86,4	80,3
62	63	65,5	61,5	63,5	62	63	63,5	64,5	62	63	63
74,7	72,6	74,7	66,5	69,1	67,6	75,2	72,1	75,2	66,5	71,1	69,6
62	66	67,1	63	67,6	68,1	60,5	62	66,5	64	64,5	68,1
71,1	70,6	72,6	73,2	73,2	74,7	70,1	71,6	73,2	74,2	72,6	76,2
81,3	76,2	79,2	79,2	79,2	75,2	70,1	75,7	79,2	80,3	80,3	77,7
86,4	85,3	86,4	83,8	85,3	85,3	85,3	87,4	86,4	86,4	84,3	85,3
86,4	83,8	84,3	89,9	85,3	86,4	84,3	86,4	85,3	90,4	88,4	86,4
77,2	74,2	73,2	71,1	73,2	75,2	75,2	75,2	75,2	72,1	73,7	75,2
90,4	92,5	90,4	86,4	87,4	88,4	89,4	89,9	89,4	87,4	87,4	88,4
79,2	76,7	78,2	76,2	78,2	76,2	78,2	75,2	78,2	75,7	79,2	75,2
76,2	75,2	73,2	76,2	77,2	75,2	76,2	75,7	77,2	76,7	76,2	74,2
70,1	68,1	66	64	68,6	69,1	70,1	67,6	68,1	63,5	69,1	68,1
82,3	84,3	84,3	84,3	84,3	82,3	83,3	82,3	83,8	84,3	84,8	81,3
87,4	87,4	83,3	88,4	83,3	84,8	87,9	91,4	84,3	88,4	85,3	87,4
70,6	73,2	72,1	74,7	73,2	72,1	69,1	71,6	73,2	74,2	72,1	72,6
96,5	94,5	94,5	96,5	95,5	96,5	95,5	91,4	97,5	98,6	91,4	97,5
78,2	80,3	78,2	77,2	77,7	78,2	78,7	79,2	75,2	78,2	80,3	80,3
59,9	55,9	57,4	59,3	58,4	62	59,4	57,6	57,6	60,5	61	62

**LH**						**RH**					
76,2	69,6	76,7	72,1	74,2	75,2	76,2	68,1	76,2	73,2	74,2	73,2

76,2	77,2	81,3	80,3	79,8	79,2	76,7	77,7	81,3	80,3	80,3	79,2
70,6	71,6	73,2	72,6	76,2	74,2	70,6	73,2	74,2	73,2	76,2	74,7
89,4	85,3	88,4	88,4	90,4	92,5	89,4	84,3	93,5	90,4	95,5	90,4
80,3	78,7	80,3	80,3	85,3	79,2	80,3	81,3	80,3	79,8	84,3	80,3
94,5	91,4	89,4	93,5	92,5	88,4	94,5	89,4	90,4	93,5	93,5	89,4
86,4	85,3	85,9	88,4	89,4	86,4	85,3	88,4	87,4	88,4	90,4	87,4
71,6	75,7	72,6	78,2	76,2	76,2	74,2	76,2	69,1	77,2	76,7	75,2
81,3	79,2	78,2	80,3	77,2	80,3	79,2	80,3	79,8	80,8	78,2	79,8
81,3	82,3	76,2	76,7	76,2	75,2	80,3	81,3	77,2	76,2	76,2	76,7
60,6	61	63,5	62	58,9	60,5	62,5	62	61,3	62,5	59,4	60,5
77,2	75,2	77,2	81,3	78,2	78,2	77,7	76,7	79,2	80,3	77,7	76,2
77,2	79,2	80,8	77,7	80,3	79,2	78,7	80,3	78,2	73,2	81,3	79,2
78,2	79,8	81,3	82,3	83,3	80,3	79,2	80,3	78,2	83,3	80,3	83,3
78,2	77,7	77,2	78,2	79,2	78,7	78,7	76,2	77,2	78,2	79,2	81,3
78,2	73,2	73,2	71,6	76,7	74,2	77,2	72,1	74,2	72,1	77,2	74,7
87,4	88,4	82,3	93,5	90,4	90,4	92,5	91,4	87,4	90,4	90,4	89,4
70,6	68,1	72,1	65	69,6	67,6	71,1	69,1	70,1	67,1	69,1	67,1
86,4	85,3	83,3	84,8	83,3	87,4	85,3	87,4	81,3	84,3	84,3	84,3
85,3	83,3	85,3	82,3	83,3	80,3	85,3	85,3	85,3	83,3	82,8	79,8
72,1	72,1	72,1	70,1	69,6	72,6	71,6	72,6	73,2	71,6	69,6	73,2
79,2	79,2	80,3	84,3	83,3	82,3	79,2	78,2	79,8	82,3	81,8	82,3
80,3	59,9	80,3	62	85,3	76,2	83,3	63	81,3	61,5	80,3	76,2
61,6	62	65	61	61,5	63	62,5	64	65,5	59,9	63,5	62,5
74,7	72,1	75,2	65	69,6	70,6	73,2	73,7	75,2	65	69,1	72,1
55,9	65,5	67,6	64	67,6	68,6	62	66,5	66,5	66	68,6	65
69,6	77,2	70,6	73,2	72,6	75,2	70,6	70,6	70,1	72,6	72,1	73,7
81,3	74,2	79,8	77,2	75,2	75,2	82,3	74,2	78,2	78,7	71,1	76,7
85,3	86,4	87,4	82,8	83,3	83,3	83,3	87,4	86,4	86,4	83,3	84,3
87,4	84,3	83,3	90,4	87,4	85,3	88,4	86,4	84,3	88,4	85,3	86,4
75,7	73,2	73,7	71,6	72,6	73,7	74,2	73,7	72,1	71,6	72,6	76,2
90,4	91,4	44,7	88,4	68,1	86,4	88,4	90,4	65,5	88,4	87,4	90,4
78,7	76,7	75,7	76,7	79,2	75,7	78,2	77,2	77,2	77,2	77,2	76,2

72,1	74,2	74,2	75,2	77,2	75,2	74,7	74,7	71,1	76,2	75,2	77,2
68,6	67,1	66	64	68,1	65	68,1	66	66,5	66	70,1	69,6
82,3	84,3	85,3	83,3	81,3	82,3	83,3	84,8	85,3	83,3	83,3	81,3
88,4	90,4	84,3	88,4	82,3	84,3	85,3	81,3	79,2	86,4	83,3	81,3
71,1	70,1	74,7	72,6	72,6	72,6	71,1	71,6	75,2	70,1	70,1	70,6
93,5	90,4	95,5	95,5	89,4	92,5	94,5	94,5	95,5	97,5	90,4	96,5
77,2	79,2	75,2	76,2	75,2	79,2	78,2	77,2	82,3	78,2	76,2	78,7
60,5	56,4	57,4	59,9	61	62	59,9	56,9	56,9	59,9	62	62

Stride Velocity (cm/sec)

**LF**						**RF**					
105,7	100,1	105,8	103	95	96,4	103,7	98,1	103	100,2	94,3	95,7
100,3	101,6	104,2	98	104,2	100,3	100,3	101,6	104,3	98	101,6	100,3
91,2	92,2	103	98,9	101,6	104,4	89,9	94,1	103	96,9	103	98,9
110,5	94,5	95,5	110,1	109	98,3	102,8	99,2	94,2	107,6	100,4	96
104,3	104,2	107,8	96,6	102,9	97,8	100,3	104,3	108,5	97,9	113,6	96,5
100,5	97,3	96,3	99,4	105	98,2	102,7	96,3	92,3	98,4	103,9	95,1
88,9	95	91	99,2	102,7	91,9	90,1	94	91,9	95,9	105,1	96,1
105,1	106	101,6	103	104,4	98,2	101,6	101,6	104,4	103	105,8	96,3
105,7	98,4	108,5	94,3	99,1	97,8	108,3	96,6	107,1	99,1	98,4	98,4
105,6	100,3	94,2	98,4	90,7	93,1	106,9	100,3	91,3	99	91,9	94,8
106,9	92,2	104,1	107,7	92,1	93,8	96	93	101,6	102,4	92,1	91,9
95,6	96,8	99,1	101,6	104,2	101	95,6	90,4	96	101,6	94,3	99
92,1	95,6	99,1	92,3	101,6	96,6	96,6	98	101,6	94	99,7	97,3
95,4	100,4	96,8	100,3	101,6	97,3	96	100,4	96	99,7	102,2	96,8
100,3	98,4	92,1	95,4	86,1	95,6	99,7	102,9	93,2	96	92,4	93,3
104,3	93,8	96,4	91,4	97,1	94	99,6	91,4	94,4	91,7	97,8	94
102,8	106,2	100,4	90,8	100,4	97,2	101,6	100,5	94	100,4	100,5	101,6
106,9	103,9	109,5	109,1	101,6	98,7	104,6	100,1	108,5	106,5	103,1	97,9
108,8	101,6	109,6	109,2	104,1	110	113,3	104,8	108,3	108	104,1	115
101,6	98,1	99,2	95,3	92,8	93,7	105,2	100,4	97	95,8	92,8	89,5
98,9	96,9	99,5	94,8	88,6	99,5	96,9	96,9	102,3	89,6	90,5	97,5

96,8	95,6	95,7	99,2	93,1	98	95,6	95,6	93,3	100,4	96,8	100,4
110,1	113,6	98,5	105,5	108,9	104,3	106,3	110,3	102,9	106,8	116,7	105,6
93,9	95,4	113	90,4	99,2	93,9	96,9	96,2	109,3	89,8	95,4	92,6
108,2	99,5	108,2	89,9	94,6	90,1	113,9	100,2	104,4	93,7	98,8	94
96,8	108,3	104,8	100	110,8	106,4	93	103,3	100,8	103,2	99,3	104,7
91,2	91,7	90,8	90,3	91,4	92,2	92,2	93	91,4	90,4	93,1	95,3
117,8	105,8	107,1	100,3	96,6	98,9	89,9	100,9	110,1	100,3	97,9	99,6
105,3	106,7	102,8	101	101,6	101,6	104,1	112	102,8	105,3	102,8	99,2
96	91,1	90,7	102,2	94,8	92,9	93,7	93,9	92,8	102,8	96,1	91,9
104,3	104,5	107,6	93,6	93,8	104,4	104,4	104,4	105,9	94,9	93,2	103
107,6	112,8	105,1	102,8	106,6	107,8	106,4	109,7	104	99,3	104	105,2
110,1	105,1	105,7	95,3	91	90,7	108,7	101,6	108,7	93,4	96,6	89,5
97,7	96,4	96,3	103	97,7	96,4	97,7	94,6	104,3	99,6	97,7	93,9
109,5	106,4	106,5	95,5	105,5	108	111,3	103,9	106,4	93,4	104,7	108,1
101,6	109,5	111	105,4	111	105,5	101,6	111,2	107,5	105,4	108,8	105,6
99,3	104	92,6	105,2	90,6	96,4	98,7	106,3	93,7	105,2	92,8	101,6
108,6	104,5	104,5	106,7	98,9	100,2	104,7	102,3	107,6	106	100,2	100,9
109,7	105	107,4	104,9	101,6	107,2	103,8	103,9	113,4	109,5	95,3	108,4
97,8	102,9	93,1	91,9	94,8	95,4	98,4	99,1	87,4	92	95,6	101,6
95,1	87,3	91,1	94,6	94,2	100	91,4	90,9	90,9	96	99,9	98,4

**LH**						**RH**					
105,8	99,4	106,5	97,5	92,7	94	107,3	97,2	105,8	98,9	95,1	93,8
100,3	101,6	104,2	97,9	101	101,6	98,3	99,6	99,1	100,3	100,3	99,1
91,7	98,1	101,6	96,9	103	100,2	90,5	98,9	101,6	98,9	101,6	100,9
101,6	97	94	102,8	105,1	110,1	106,4	98,1	101,6	110,3	113,7	102,8
102,9	99,7	105,6	102,9	115,3	96,6	102,9	99,1	102,9	101	115,5	100,3
105	93,3	89,4	97,4	102,7	94	102,7	93,1	90,4	99,4	106,2	95,1
86,4	90,8	88,5	102,8	95,1	90	87,1	98,2	91	98,2	100,5	93
98,1	109,7	94,3	102,9	100,3	100,3	100,2	112,1	90,9	101,6	100,9	101,6
106,9	96,6	100,3	100,3	94,2	100,3	110,1	100,3	103,6	101	100,3	99,7

106,9	100,4	92,9	92,4	88,6	91,7	104,2	99,1	94,2	90,7	87,6	93,5
99,9	95,3	100,8	100	90,7	88,9	107,7	95,3	95,8	102,4	91,4	88,9
89,8	85,4	91,9	99,1	93,1	95,4	93,6	87,2	99,1	96,7	93,6	95,3
94,2	93,2	97,3	93,6	95,6	99,1	92,6	95,6	95,4	89,2	99,1	96,6
91	98,5	99,1	102,9	104,1	93,3	94,3	102,9	93,1	104,1	102,9	99,2
95,4	97,2	91,9	93,1	87,1	91,6	96	92,9	88,8	94,3	86,1	94,5
100,3	87,1	89,2	84,3	95,9	88,3	99	86,9	92,7	85,9	96,5	90
93	98,2	85,7	101,6	102,8	100,5	100,5	103,9	95	100,5	100,5	99,3
103,8	104,7	107,7	108,4	103,9	95,2	104,6	106,3	103,1	109,9	104,7	90,6
107,9	109,4	109,6	103,5	99,2	112	106,7	106,6	109,8	102,8	100,4	105,4
101,6	92,6	97	95,7	90,6	87,2	99,2	97	99,2	88,6	89	85,8
96,2	94,9	97,5	92,2	89,2	96,9	94,2	96,9	97,5	94,2	88,1	97,5
94,3	94,3	95,6	102,8	101,6	102,9	94,3	88,9	93,8	98	97,4	100,4
108,5	99,9	100,3	106,9	106,7	99	112,6	92,6	101,6	89,1	97,9	100,3
94,3	92,5	110,2	87,1	93,1	92,6	96,1	97	109,2	85,6	94,8	93,3
108,2	100,2	110,6	87,9	92,8	94,1	104,5	99,5	109	87,9	93,4	97,5
90,1	109,2	105,6	103,2	118,5	107,2	96,8	109,1	107,3	103,2	114,3	100
91,6	113,6	86,1	89,2	92	91,7	95,4	90,5	87,6	89,7	92,5	89,8
116,1	100,2	112,3	96,5	92,8	94	124,7	103	111,8	99,7	96,1	98,3
101,6	108	106,6	97,4	101,6	99,2	96,9	106,6	110,7	105,3	96,9	102,8
95	91,7	90,6	100,5	95	88,9	96,1	93,9	91,7	96,1	92,8	88,1
100,9	107,6	105,2	93	94,3	102,3	95,1	103,7	101,6	93	95,6	105,8
110,3	117,2	101,6	96,1	104,7	100,4	107,8	113	104	98,2	136,5	105,1
107,9	105,1	100,9	94,7	96,6	91,2	111,8	107,2	104,3	94,2	94,2	92,9
90,2	88,3	95,1	96,4	94,2	94	92,2	91,1	91,2	99	91,7	96,5
108,9	103,2	101,6	91,4	103,1	98,5	109,8	103,2	104	95,7	104,6	105,4
102,9	105,4	106,7	104,1	104,2	108,3	106,8	107,4	109,4	104,1	106,8	104,2
98,2	105,1	86	107,8	85,7	98,1	97	99,1	86,1	105,3	88,6	90,3
111,1	100,1	106,7	100,9	98,2	98,2	107,8	100,9	110,6	97,4	94,7	92,9
106,2	102,8	111,1	108,5	97,2	98,4	105	102,7	113,7	108,4	96,2	104,9
94,2	96,6	90,6	82,8	89,5	96,6	94,3	94,2	117,6	86,9	92,9	93,7
97,5	88,1	88,3	94,6	98,3	101,6	95,1	88,9	89,8	96,7	103,3	98,4

Peak vertical force (N)

LF						RF					
146,224	151,883	145,821	157,071	150,106	161,613	144,412	144,64	144,991	158,088	147,894	154,857
115,839	120,06	125,175	117,246	117,849	109,785	117,078	106,111	109,911	106,906	114,419	114,674
141,906	138,026	136,878	132,959	143,294	143,904	137,195	137,611	142,797	133,635	133,615	136,74
210,406	196,402	204,124	181,877	184,543	206,046	189,254	200,157	210,312	202,081	190,257	198,553
163,225	169,22	166,701	165,227	167,752	164,886	167,595	173,021	175,858	162,25	175,305	161,733
175,139	180,936	225,375	215,291	180,129	210,182	204,614	205,068	205,573	192,885	213,63	177,151
191,91	184,894	182,824	189,157	186,387	186,925	192,963	196,283	181,655	177,307	181,17	187,585
147,372	150,456	147,395	156,675	147,338	150,937	144,243	140,646	136,081	140,156	147,429	136,688
201,941	189,491	189,894	199,123	207,717	203,021	191,916	202,66	225,24	193,759	203,434	210,616
162,764	161,329	164,642	168,869	160,638	160,99	169,475	191,702	169,438	171,03	160,775	164,992
73,346	76,586	80,622	73,974	74,475	78,472	82,095	75,143	75,918	76,92	74,239	75,143
119,899	136,919	128,755	127,715	131,018	130,299	137,162	127,138	126,118	118,184	121,525	122,543
166,859	159,194	164,619	164,711	157,612	184,186	151,844	171,915	171,145	151,8	166,068	162,701
126,258	124,309	129,908	126,596	126,256	123,031	132,405	108,768	110,278	117,92	110,167	121,382
221,99	221,049	226,161	200,262	194,956	224,377	204,877	181,406	205,514	198,98	221,835	210,481
186,647	179,895	186,337	178,14	175,274	172,021	160,919	168,213	161,472	187,723	177,699	172,429
175,568	155,449	161,51	158,125	146,953	147,068	179,99	160,079	159,098	159,749	156,495	153,943
81,92	93,128	84,228	80,054	87,881	82,621	82,956	81,347	77,811	81,424	87,364	83,037
143,954	146,657	140,697	141,338	135,196	148,671	144,256	135,929	132,71	138,248	144,932	144,586
155,026	154,36	164,042	159,691	161,69	162,798	151,959	152,772	154,536	173,774	156,545	161,749
105,321	103,212	101,135	97,885	107,414	112,272	101,642	98,389	101,466	98,106	100,777	104,417
198,252	166,025	169,11	168,421	170,745	195,006	168,857	185,78	166,147	162,199	167,622	166,156
154,393	169,187	152,837	142,314	150,43	151,96	138,698	147,27	139,84	144,686	151,137	141,108
110,651	129,043	123,775	113,493	116,387	114,434	111,581	119,671	113,197	111,342	114,118	112,485
84,052	83,305	95,721	81,961	81,435	83,07	97,807	91,48	92,701	93,237	91,577	85,781
97,916	85,097	90,197	92,386	86,992	94,847	88,881	94,957	95,14	88,548	94,187	91,346
153,141	160,173	152,608	153,645	149,189	148,535	153,501	148,077	143,546	148,79	148,647	148,763
99,998	100,885	95,965	100,714	89,425	99,208	85,348	99,352	95,224	96,502	96,829	92,952
186,163	167,784	170,234	184,543	172,171	182,049	172,487	174,868	170,087	185,579	167,09	190,135

169,363	162,941	169,228	166,556	172,953	166,987	171,608	161,503	176,463	173,69	179,182	159,877
97,586	95,233	93,094	99,589	97,201	100,067	94,084	91,542	98,823	98,67	101,441	93,601
166,355	199,829	162,73	199,865	163,031	166,18	168,099	175,947	172,153	171,4	167,265	171,834
195,275	170,354	193,35	175,683	184,295	179,354	195,756	182,072	185,058	185,065	178,19	175,902
171,683	156,351	160,296	143,06	161,643	167,579	164,844	160,938	143,577	154,064	159,098	161,951
63,599	63,81	63,329	66,802	67,39	70,604	69,57	69,101	70,086	65,044	63,945	62,724
111,099	114,886	115,561	108,416	113,592	115,931	107,245	111,669	115,87	120,455	128,722	109,962
174,584	152,031	149,616	150,276	155,904	151,956	157,491	149,331	159,669	148,455	149,356	144,74
118,765	112,649	109,863	107,575	112,772	115,328	113,277	109,442	110,441	118,085	107,585	105,338
191,987	188,957	183,279	181,953	182,689	197,771	192,882	185,826	189,895	188,961	195,789	186,972
18,864	19,438	17,87	17,919	17,393	18,927	17,758	18,455	20,679	17,05	19,409	19,05
87,534	82,099	74,879	82,26	72,741	86,435	76,366	75,55	76,252	71,707	83,222	79,159

**LH**						**RH**					
84,972	85,194	87,6	95,117	80,335	80,339	93,768	86,81	86,891	75,866	70,651	70,984
78,932	77,398	77,745	70,732	66,314	68,892	69,917	67,048	68,422	70,597	71,113	76,096
95,825	114,028	110,203	103,061	111,746	104,549	105,077	110,141	116,637	111,822	111,234	114,63
109,542	99,958	93,277	124,966	113,14	111,957	112,411	99,673	107,873	133,812	115,234	104,656
83,994	80,431	83,151	91,997	88,965	80,364	83,927	76,768	83,796	88,511	93,543	84,939
128,651	115,802	111,86	98,761	128,533	121,238	110,488	98,963	89,554	126,552	113,783	99,146
108,148	118,037	137,717	137,259	133,187	136,211	121,443	133,865	130,167	139,003	131,051	125,813
88,877	95,43	90,486	96,447	107,117	95,662	84,047	93,878	100,975	92,267	91,739	88,856
114,728	96,043	92,231	101,811	102,464	104,881	89,751	92,267	93,28	93,705	95,87	89,727
107,653	88,314	102,752	90,668	99,72	87,886	90,646	93,52	86,611	88,592	85,188	99,029
52,419	51,624	50,563	52,203	54,776	49,895	45,251	46,606	47,097	47,146	45,948	44,838
69,307	67,964	73,103	83,535	72,639	86,721	82,583	69,058	79,583	76,595	82,938	87,5
94,602	90,896	94,175	84,459	90,465	98,173	96,263	101,312	104,263	102,736	95,095	96,381
77,36	63,157	60,752	55,986	57,98	58,932	59,8	70,867	73,92	70,304	74,119	75,074
143,519	149,165	127,043	155,093	113,213	122,832	146,099	155,422	145,258	135,745	129,778	120,218
106,848	96,239	100,668	98,836	100,519	97,283	99,247	89,17	96,57	100,618	102,862	93,944
87,554	91,018	84,936	115,044	115,663	97,171	89,417	103,452	82,501	93,714	106,459	99,541

58,399	45,997	52,653	53,034	45,978	45,243	54,468	49,399	53,444	51,321	46,963	45,835
92,514	82,169	84,068	81,695	87,659	84,937	85,419	87,005	90,543	78,93	77,689	86,299
83,114	81,85	78,39	80,879	77,498	81,869	76,234	75,538	77,675	79,39	79,488	75,303
48,515	51,297	53,902	50,373	48,276	46,244	43,971	40,58	48,045	46,804	42,253	46,858
105,719	114,074	119,445	124,621	118,73	130,9	110,436	105,706	107,753	115,156	106,989	114,876
117,778	97,334	109,166	104,662	103,157	103,973	110,259	93,682	106,491	101,057	96,546	109,054
58,907	51,835	64,527	55,329	51,709	57,157	56,88	49,186	61,012	47,104	49,828	49,188
74,109	81,179	74,388	59,044	63,95	68,153	75,388	73,75	76,693	70,124	62,157	68,357
58,199	68,368	60,857	63,762	68,23	59,774	68,848	77,059	68,447	64,34	64,27	60,609
93,016	83,053	80,806	88,661	82,877	84,813	65,799	91,925	87,034	84,636	93,633	82,064
56,809	59,799	53,487	58,578	49,478	49,393	66,086	57,659	61,084	55,084	55,473	53,294
113,987	112,572	110,534	92,156	109,549	111,076	112,358	106,813	131,678	117,111	101,074	119,142
93,772	89,67	93,58	95,073	84,276	82,064	78,535	76,901	80,351	84,936	82,263	79,596
56,781	51,982	55,143	52,863	49,288	54,882	56,161	53,317	57,337	53,057	52,253	61,147
95,617	93,345	94,146	83,875	99,692	97,035	102,664	105,387	97,547	88,631	86,59	103,297
147,581	134,779	144,197	124,756	127,407	122,985	134,994	145,077	128,798	119,695	112,213	105,293
78,739	73,192	80,473	84,672	73,74	76,462	79,923	78,969	77,028	87,009	79,919	82,521
41,511	44,363	40,627	42,047	41,345	38,676	40,579	40,591	42,707	40,075	36,324	39,35
70,798	75,937	60,467	66,759	65,414	64,243	60,048	68,911	74,234	58,686	58,445	59,162
88,883	100,439	88,225	110,807	89,601	91,741	83,841	92,402	95,816	96,573	75,676	79,971
84,395	76,764	84,015	76,937	74,736	70,404	77,643	77,343	81,288	85,567	79,735	86,868
98,804	100,53	109,928	95,611	111,455	108,637	112,073	102,018	110,967	105,098	96,735	95,9
11,849	10,739	9,888	9,43	10,736	10,239	10,049	10,575	9,815	9,803	10,646	10,167
60,139	46,378	42,941	54,202	43,685	58,819	54,017	44,427	49,899	55,284	54,362	53,8

Peak vertical force (%BW)

**LF**						**RF**					
53,3	55,3	53,1	57,2	54,7	58,9	52,6	52,7	52,8	57,6	53,9	56,4
53,7	55,6	58	54,3	54,6	50,9	54,3	49,2	50,9	49,6	53	53,2
50,8	49,4	49	47,6	51,3	51,5	49,1	49,2	51,1	47,8	47,8	48,9
63,1	58,9	61,2	54,5	55,3	61,8	56,8	60	63,1	60,6	57,1	59,5

54,4	56,4	55,6	55,1	55,9	54,9	55,8	57,7	58,6	54,1	58,4	53,9
48,9	50,5	63	60,1	50,3	58,7	57,2	57,3	57,4	53,9	59,7	49,5
52	50,1	49,6	51,3	50,5	50,7	52,3	53,2	49,3	48,1	49,1	50,9
54,6	55,8	54,7	58,1	54,6	56	53,5	52,2	50,5	52	54,7	50,7
66,4	62,3	62,5	65,5	68,3	66,8	63,1	66,7	74,1	63,7	66,9	69,3
51,9	51,4	52,5	53,8	51,2	51,3	54	61,1	54	54,5	51,2	52,6
49,8	52	54,7	50,2	50,6	53,3	55,7	51	51,5	52,2	50,4	51
47,4	54,1	50,9	50,5	51,8	51,5	54,2	50,2	49,8	46,7	48	48,4
56,5	53,9	55,8	55,8	53,4	62,4	51,4	58,2	58	51,4	56,3	55,1
57,5	56,6	59,1	57,6	57,5	56	60,3	49,5	50,2	53,7	50,2	55,3
58,3	58,1	59,4	52,6	51,2	59	53,8	47,7	54	52,3	58,3	55,3
58,6	56,4	58,5	55,9	55	54	50,5	52,8	50,7	58,9	55,8	54,1
64,2	56,8	59	57,8	53,7	53,8	65,8	58,5	58,1	58,4	57,2	56,3
54,6	62,1	56,1	53,4	58,6	55,1	55,3	54,2	51,9	54,3	58,2	55,3
55,2	56,2	53,9	54,2	51,8	57	55,3	52,1	50,9	53	55,6	55,4
53,8	53,6	56,9	55,4	56,1	56,5	52,7	53	53,6	60,3	54,3	56,1
56,5	55,4	54,3	52,5	57,6	60,3	54,6	52,8	54,5	52,7	54,1	56
59,1	49,5	50,4	50,2	50,9	58,1	50,3	55,4	49,5	48,4	50	49,5
53,2	58,3	52,7	49	51,8	52,4	47,8	50,7	48,2	49,8	52,1	48,6
61	71,1	68,2	62,6	64,2	63,1	61,5	66	62,4	61,4	62,9	62
46	45,5	52,3	44,8	44,5	45,4	53,5	50	50,7	51	50,1	46,9
55,5	48,2	51,1	52,3	49,3	53,7	50,4	53,8	53,9	50,2	53,4	51,7
52,9	55,4	52,8	53,1	51,6	51,3	53,1	51,2	49,6	51,4	51,4	51,4
52,8	53,3	50,7	53,2	47,2	52,4	45,1	52,5	50,3	51	51,2	49,1
57,4	51,7	52,4	56,9	53	56,1	53,1	53,9	52,4	57,2	51,5	58,6
58,1	55,9	58,1	57,2	59,4	57,3	58,9	55,5	60,6	59,6	61,5	54,9
52,9	51,7	50,5	54	52,7	54,3	51	49,7	53,6	53,5	55	50,8
55,4	66,6	54,2	66,6	54,3	55,4	56	58,6	57,4	57,1	55,7	57,3
57,6	50,2	57	51,8	54,3	52,9	57,7	53,7	54,5	54,5	52,5	51,8
60,4	55	56,4	50,3	56,8	58,9	58	56,6	50,5	54,2	55,9	56,9
42,7	42,8	42,5	44,8	45,2	47,4	46,7	46,4	47	43,6	42,9	42,1
56,6	58,6	58,9	55,3	57,9	59,1	54,7	56,9	59,1	61,4	65,6	56,1

59,9	52,2	51,4	51,6	53,5	52,2	54,1	51,3	54,8	51	51,3	49,7
46,6	44,2	43,1	42,2	44,2	45,2	44,4	42,9	43,3	46,3	42,2	41,3
57,1	56,2	54,5	54,1	54,3	58,8	57,3	55,2	56,5	56,2	58,2	55,6
55,5	57,2	52,6	52,7	51,2	55,7	52,2	54,3	60,8	50,1	57,1	56
55,1	51,7	47,1	51,8	45,8	54,4	48,1	47,6	48	45,1	52,4	49,8

**LH**						**RH**					
30,9	31	31,9	34,6	29,3	29,3	34,1	31,6	31,6	27,6	25,7	25,9
36,6	35,9	36	32,8	30,7	31,9	32,4	31,1	31,7	32,7	33	35,3
34,3	40,8	39,4	36,9	40	37,4	37,6	39,4	41,7	40	39,8	41
32,9	30	28	37,5	33,9	33,6	33,7	29,9	32,4	40,1	34,6	31,4
28	26,8	27,7	30,7	29,6	26,8	28	25,6	27,9	29,5	31,2	28,3
35,9	32,4	31,3	27,6	35,9	33,9	30,9	27,6	25	35,4	31,8	27,7
29,3	32	37,3	37,2	36,1	36,9	32,9	36,3	35,3	37,7	35,5	34,1
33	35,4	33,6	35,8	39,7	35,5	31,2	34,8	37,4	34,2	34	32,9
37,7	31,6	30,3	33,5	33,7	34,5	29,5	30,4	30,7	30,8	31,5	29,5
34,3	28,1	32,7	28,9	31,8	28	28,9	29,8	27,6	28,2	27,1	31,6
35,6	35	34,3	35,4	37,2	33,9	30,7	31,6	32	32	31,2	30,4
27,4	26,9	28,9	33	28,7	34,3	32,6	27,3	31,5	30,3	32,8	34,6
32	30,8	31,9	28,6	30,6	33,3	32,6	34,3	35,3	34,8	32,2	32,7
35,2	28,8	27,7	25,5	26,4	26,8	27,2	32,3	33,7	32	33,7	34,2
37,7	39,2	33,4	40,8	29,8	32,3	38,4	40,8	38,2	35,7	34,1	31,6
33,5	30,2	31,6	31	31,5	30,5	31,1	28	30,3	31,6	32,3	29,5
32	33,3	31	42	42,3	35,5	32,7	37,8	30,2	34,3	38,9	36,4
38,9	30,7	35,1	35,3	30,6	30,2	36,3	32,9	35,6	34,2	31,3	30,5
35,5	31,5	32,2	31,3	33,6	32,6	32,7	33,4	34,7	30,3	29,8	33,1
28,8	28,4	27,2	28,1	26,9	28,4	26,5	26,2	27	27,6	27,6	26,1
26	27,5	28,9	27	25,9	24,8	23,6	21,8	25,8	25,1	22,7	25,1
31,5	34	35,6	37,2	35,4	39	32,9	31,5	32,1	34,3	31,9	34,3
40,6	33,5	37,6	36,1	35,5	35,8	38	32,3	36,7	34,8	33,3	37,6
32,5	28,6	35,6	30,5	28,5	31,5	31,4	27,1	33,6	26	27,5	27,1

40,5	44,4	40,7	32,3	35	37,3	41,2	40,3	41,9	38,3	34	37,4
33	38,7	34,5	36,1	38,7	33,9	39	43,7	38,8	36,4	36,4	34,3
32,2	28,7	27,9	30,6	28,6	29,3	22,7	31,8	30,1	29,3	32,4	28,4
30	31,6	28,3	30,9	26,1	26,1	34,9	30,5	32,3	29,1	29,3	28,2
35,1	34,7	34,1	28,4	33,7	34,2	34,6	32,9	40,6	36,1	31,1	36,7
32,2	30,8	32,1	32,6	28,9	28,2	27	26,4	27,6	29,2	28,2	27,3
30,8	28,2	29,9	28,7	26,7	29,8	30,5	28,9	31,1	28,8	28,3	33,2
31,9	31,1	31,4	28	33,2	32,3	34,2	35,1	32,5	29,5	28,9	34,4
43,5	39,7	42,5	36,8	37,5	36,2	39,8	42,8	38	35,3	33,1	31
27,7	25,7	28,3	29,8	25,9	26,9	28,1	27,8	27,1	30,6	28,1	29
27,8	29,8	27,3	28,2	27,7	25,9	27,2	27,2	28,7	26,9	24,4	26,4
36,1	38,7	30,8	34	33,4	32,8	30,6	35,1	37,8	29,9	29,8	30,2
30,5	34,5	30,3	38	30,8	31,5	28,8	31,7	32,9	33,2	26	27,5
33,1	30,1	33	30,2	29,3	27,6	30,5	30,3	31,9	33,6	31,3	34,1
29,4	29,9	32,7	28,4	33,1	32,3	33,3	30,3	33	31,2	28,8	28,5
34,9	31,6	29,1	27,7	31,6	30,1	29,6	31,1	28,9	28,8	31,3	29,9
37,9	29,2	27	34,1	27,5	37	34	28	31,4	34,8	34,2	33,9

Vertical impulse (N*sec)

**LF**						**RF**					
48,393	50,86	49,925	56,673	56,299	60,914	47,434	46,601	48,782	51,741	53,299	57,068
41,071	41,634	44,732	45,881	42,537	39,754	42,139	37,517	40,777	40,348	42,748	41,416
46,252	44,104	45,773	44,352	47,599	47,756	51,183	47,44	47,925	48,148	47,713	48,507
84,506	82,83	81,044	74,995	77,845	90,268	76,865	79,648	95,707	75,565	75,831	84,551
58,828	61,991	56,392	63,36	58,813	60,692	57,597	59,685	58,919	56,586	55,104	61,397
76,212	81,718	103,201	95,537	73,225	91,507	89,059	96,303	92,99	83,869	90,253	76,93
89,056	83,009	84,588	78,065	82,641	82,748	87,307	83,401	80,908	75,93	75,561	82,732
46,079	45,073	48,218	49,418	47,005	50,883	45,942	42,689	47,893	46,821	49,593	47,187
68,313	66,949	62,229	75,465	77,585	74,572	59,013	70,862	76,033	69,964	69,392	73,272
60,707	64,106	67,886	68,099	69,102	66,233	60,241	73,118	65,388	66,814	69,355	67,47
20,789	23,401	23,421	21,27	23,696	24,962	23,077	24,157	23,126	22,89	23,185	24,413
48,919	53,886	49,574	48,951	50,86	48,279	56,475	54,78	52,418	47,277	47,874	46,046

65,951	64,344	61,603	62,989	60,841	67,927	60,906	66,637	65,371	61,752	63,091	65,609
42,386	44,067	45,761	42,298	42,343	44,452	49,129	39,528	41,312	40,966	39,692	50,958
81,058	80,805	91,559	77,403	81,452	87,82	75,591	70,04	86,643	76,547	98,344	85,199
69,551	71,497	71,877	72,091	68,863	70,139	61,977	67,697	63,019	74,627	69,014	70,092
76,284	66,271	67,678	66,551	60,527	61,787	69,029	63,242	69,173	59,423	59,842	63,351
27,164	28,973	27,051	22,807	29,564	27,913	25,958	25,797	24,179	23,744	28,219	28,055
51,824	54,054	49,067	56,564	50,034	54,827	53,79	51,555	47,808	51,222	57,905	52,172
62,132	68,012	68,639	71,099	69,786	69,962	64,641	66,415	65,552	75,342	73,186	75,46
37,914	37,153	36,471	36,211	40,083	37,449	35,864	36,231	33,252	35,755	36,474	37,172
78,795	69,855	67,81	68,781	67,969	75,797	70,743	77,021	69,164	64,844	66,274	63,479
53,661	60,193	57,033	52,246	53,712	53,455	50,067	56,333	52,467	52,271	57,917	48,949
32,33	36,422	29,864	34,684	33,084	33,158	31,611	34,487	28,057	33,965	33,495	32,376
26,395	26,512	27,558	27,191	28,082	28,667	30,877	30,441	27,873	32,479	32,81	30,929
26,345	22,062	25,522	25,098	22,212	27,714	28,258	26,748	27,75	24,327	25,615	27,943
55,878	58,415	60,602	61,641	55,805	57,929	53,68	56,54	56,295	57,91	55,578	57,899
29,839	34,901	30,507	35,191	34,503	35,75	25,604	33,07	31,41	36,99	37,707	34,649
70,469	58,769	65,482	74,872	66,582	69,606	64,537	64,563	63,258	71,734	64,758	73,425
66,758	65,956	69,887	67,562	70,725	68,607	68,423	67,959	72,984	69,61	71,845	72,981
32,466	31,54	30,208	35,451	34,056	33,904	33,37	31,397	32,895	36,077	37,764	32,494
61,631	72,923	62,226	78,229	64,454	63,364	64,229	66,693	69,032	71,899	66,64	69,724
62,957	59,89	64,729	68,363	75,05	73,667	58,321	59,743	60,145	70,56	71,049	69,195
64,55	60,391	55,128	54,564	62,848	63,284	59,341	61,387	52,736	53,614	58,288	60,115
18,205	18,753	18,558	20,271	20,838	19,953	20,001	20,101	20,126	19,228	18,786	17,293
40,049	37,825	36,438	39,632	37,383	39,682	38,731	37,469	39,727	42,925	42,625	38,343
76,283	60,824	65,076	62,946	71,629	67,587	67,956	60,384	70,152	62,981	69,734	63,939
32,502	35,77	32,917	34,18	38,151	38,157	32,396	34,594	32,946	38,858	35,683	36,38
72,869	78,273	70,574	73,808	77,16	79,825	77,843	79,097	69,656	76,269	84,093	77,128
7,114	7,085	7,464	7,662	6,746	6,864	7,053	7,065	8,121	7,504	7,563	7,367
26,089	25,381	22,788	24,975	21,144	25,551	21,717	21,884	21,893	20,344	23,526	22,84

LH **RH**

24,38	27,182	27,242	30,051	28,02	28,087	27,517	27,51	26,322	24,816	26,739	25,82
23,249	22,853	21,451	21,062	21,012	20,83	21,015	19,394	20,552	22,692	20,804	22,988
30,845	31,12	28,644	29,607	30,583	30,794	33,969	32,08	32,013	32,978	32,479	32,108
37,361	38,857	38,222	39,994	37,406	35,187	39,793	38,927	41,596	41,393	36,642	38,108
30,936	31,492	29,76	34,085	28,829	30,102	31,236	30,027	30,077	32,379	29,192	31,931
47,429	49,5	50,333	38,635	46,054	49,002	40,352	42,344	40,966	51,026	40,735	40,647
49,968	52,381	60,807	48,758	52,615	54,368	50,678	49,823	53,036	44,766	51,808	48,058
29,961	29,942	31,482	31,341	33,849	32,274	26,644	26,728	34,355	29,755	28,803	27,603
34,879	33,931	31,479	32,715	34,862	34,671	26,181	30,717	31,29	31,154	31,044	30,518
36,582	34,006	40,038	37,35	41,453	34,26	33,231	35,192	33,976	36,886	36,875	38,438
12,177	12,088	11,322	11,872	12,589	12,471	10,517	12,295	11,912	10,763	12,069	11,843
25,326	26,479	26,705	27,187	26,23	27,448	31,662	27,761	26,869	26,092	29,96	27,838
35,29	31,621	31,533	30,363	29,845	33,644	31,792	31,86	33,38	35,377	29,71	30,64
26,965	21,328	19,659	18,622	19,054	20,679	22,105	26,595	25,777	24,824	26,237	26,42
49,226	49,946	51,75	53,323	49,276	49,67	50,503	51,625	55,476	49,179	52,381	43,907
38,381	38,821	38,588	41,289	38,189	39,713	35,81	35,229	36,956	38,324	38,831	36,279
34,264	33,394	32,934	38,239	34,242	33,126	32,132	33,357	31,796	32,997	30,842	31,691
14,344	11,411	12,852	11,695	12,307	12,761	14,203	12,427	13,29	12,162	12,778	13,535
27,804	26,236	26,767	29,149	32,099	26,267	25,562	30,974	27,168	27,102	26,524	30,325
32,967	34,741	31,977	34,633	33,447	35,888	30,713	33,379	32,34	34,692	35,995	34,672
15,577	17,325	16,953	17,023	16,346	15,139	14,644	14,313	15,467	15,527	15,116	15,448
39,053	43,392	43,708	41,246	40,842	45,345	44,129	42,88	43,938	43,256	42,776	42,66
29,751	25,176	32,452	25,641	28,989	32,462	28,689	25,794	34,012	25,282	32,115	35,808
12,301	11,281	11,643	12,667	11,163	13,068	12,212	10,798	10,994	12,693	11,965	12,345
17,843	21,827	17,464	18,453	18,471	20,521	18,026	19,856	18,461	21,537	18,85	19,95
16,146	15,06	14,467	14,431	13,54	14,842	16,915	17,951	14,995	14,906	14,378	17,684
33,327	24,227	31,386	33,42	30,316	30,628	24,138	34,105	32,26	31,373	32,097	30,444
15	19,037	15,468	20,43	17,919	17,353	15,416	19,063	17,045	19,433	21,05	18,011
38,06	34,26	33,284	36,195	34,793	37,415	39,214	33,755	37,016	37,91	39,045	39,467
37,416	38,182	40,343	33,714	33,187	35,794	31,957	32,069	32,122	34,581	34,373	35,319
16,694	14,635	15,115	17,289	16,47	15,945	17,188	16,132	19,205	18,647	17,862	18,336
30,799	28,667	24,349	33,351	27,549	33,148	33,635	32,189	32,158	36,844	27,016	35,431

38,12	36,281	42,447	39,961	44,474	40,83	35,394	42,18	37,621	40,051	40,393	38,021
29,004	27,654	28,182	28,991	26,995	28,904	29,095	29,295	27,53	28,447	30,428	29,997
9,875	10,798	10	10,921	10,227	9,952	10,014	10,728	11,225	10,77	9,278	9,585
19,886	20,187	18,345	19,1	19,886	17,933	16,766	19,067	19,858	16,854	16,126	16,975
34,656	32,742	35,659	36,425	42,422	36,401	35,518	33,342	38,988	33,289	34,235	35,757
22,091	22,566	24,221	23,575	23,115	22,547	20,91	23,703	22,464	24,052	24,613	28,885
40,738	41,898	43,251	39,829	46,222	46,635	44,807	46,602	42,096	44,565	45,346	40,73
4,259	3,621	3,865	4,051	4,007	3,698	3,646	3,66	3,154	3,936	4,195	3,637
14,798	12,959	11,344	12,554	10,629	13,889	13,173	12,245	12,771	13,016	12,02	12,748

Maximum peak pressure (kPa)

**LF**						**RF**					
119,1	114,9	114,7	124,5	115,9	109,1	102,1	101,7	101,5	109,3	101,9	109,9
104,1	128,4	106,9	108,7	114,7	110,7	128,5	111,3	117,9	121,1	122,3	108,4
116,8	110,2	122	128,6	122,7	109,1	125	115,3	112,5	114,2	112,5	117,9
153,4	151	128,5	129,3	143,9	140,4	119,7	124,6	137,1	139,4	141,6	133,8
102,2	101,4	96,9	97,9	95,8	100,9	92,4	101,6	97,3	95,5	99,9	96,8
109	117,3	127,4	130,9	112,7	122,6	128	124,5	121,5	101,7	122,9	99,1
122,7	137,1	129,9	121,2	118	121,5	135,1	129,1	123	126,4	128,4	135
116,9	118	113,2	133,1	135,2	127,3	105	105,1	125,2	97,6	111,5	107,6
121,6	109,4	108,3	125,6	119,3	118,7	119,4	137,2	138,5	122,4	128,3	124,2
139,5	133,5	130,1	146,7	131,4	131	134,8	133,7	122,8	125	124,4	131,6
83,4	76,9	77,7	90,3	87,7	80,3	90,4	85	91,1	88,2	88,5	95,8
91,4	91	92,6	98,4	93,4	86,8	97,2	100,7	91,1	91,2	80,8	89,1
122,7	124,4	111,7	120,1	120,8	114,9	109,3	115,9	128	114,3	115,3	120,7
102,7	126,4	109,4	116,5	103	119,5	116	100,9	100	108,8	108,3	111,5
136,9	158,3	163,6	147	127,3	145,1	127,9	144,4	142,4	127,6	142,9	152
140,2	121,3	127,1	120,9	126,5	130,4	117	120,2	125,6	135,1	141,4	133,5
167,8	153,7	142,7	137,4	134,8	133,8	152,4	139,8	141,7	136,6	137,9	141,2
101,3	105,5	88,4	91,3	107,1	82,2	84,4	77,6	81,9	78,2	80,3	89,1
115,9	107,2	114,8	123,4	104	116	123,6	110,2	119,6	121	133	120,7
141,1	141,6	133,6	134,6	135,9	138,8	135,3	129,3	143,7	145,9	153,8	137,4

98,3	97,8	93,8	100,3	88,4	102	86,4	95,9	84,9	97	92,4	94,8
137,1	117,5	125,2	130,5	117,2	130,3	122,4	121,1	110,3	113,4	115,2	117,7
117,8	126,2	118,3	123,2	116,7	107,7	110,1	127,3	116,5	127	123,3	122,2
141,4	147,1	139,4	139,7	126,3	139	150,9	130,7	135,1	146,5	134	121,9
92,2	85,7	94,1	91	86,6	92,3	105,7	103,8	92,1	89,7	90,7	88
102,1	88,1	93,8	92,7	100,8	111,3	99,7	127,8	112,5	92,1	107,8	96,3
104,1	109,8	104,9	117,3	104,1	106,6	112,6	109,2	106	115,5	111,3	114,9
76,8	88,6	94,1	88,2	75,6	82,5	76	87,5	75,1	77,7	83,8	95,1
120,6	121,5	116,8	120,4	124,9	109,2	122,1	127,2	124,7	123,3	119,4	120,1
129,7	121,9	127,2	124,8	125,6	128,4	128,3	126,6	130,4	130	132,1	118,6
107,7	111,6	121,4	103,7	113,8	112,6	106,4	97,9	116,6	102	113	109,6
125	170,8	138,1	166,7	146,5	134	130,2	131	120	134,9	125,6	139,5
146,3	137,1	132,7	119,6	135,1	133,6	131	127,8	125,2	132,2	122,9	128,5
116,1	118	116,4	113,4	121,2	112,7	123	113,3	108,2	107,5	104,9	115,7
57	57,5	47,5	54,5	50,3	55,3	55,3	56,2	47,8	49,5	52,4	51,6
97,8	91,1	84,3	92,3	108,5	104,9	93,9	96,1	90,5	97,6	95,6	88,3
145,4	127,3	125,7	134,8	115,6	135,9	129,4	123	134,4	143,9	130,7	123,8
99,5	106,9	98,6	92,7	123,1	109,2	119,2	109,9	101,3	114,3	115	103,2
154,1	152,8	147,5	149,2	138,6	157,8	156,3	158,7	136,3	138,2	157,1	147,9
103,2	112,3	103,9	101,8	96,4	95,9	105,9	103,9	125,6	105,3	110,8	110,7
94,9	71,9	72,1	91,5	73,2	83,2	90,9	82,6	81,8	73,9	81,2	93,6

**LH**						**RH**					
88,6	80,2	92,9	89,5	88,3	81,2	90,6	89	94,5	84,6	77,5	74,7
83,2	80	68,5	65,6	75	77	66,8	77,5	74,4	80,5	74,8	79,8
93,6	86,6	85	81,4	84,3	93,7	105	97,2	93,1	91,2	97,1	101,4
82,5	84,4	96,4	91,6	84,8	87,9	92,8	81,8	88,4	97,1	88,2	88,4
99,3	89,9	95,1	98,5	92,4	79,9	87,3	78,7	88,9	82,2	82,9	94,3
104,6	105,3	97,6	76,7	87,2	103	87,7	88,9	96,1	107,6	87,5	90,6
82,5	98,4	95,6	103,6	105	105,3	94,3	87,9	93,8	87,2	94	93,6
85,8	86,1	97,3	84,4	96,4	97,5	83,2	79,9	95,6	94,5	88,8	79,1

89	81,5	80,6	76,4	86,5	85,9	77,9	88,4	85,7	89,9	86,2	87,1
107,4	97,7	96,5	99,4	99,6	98,6	99,5	99,1	100,6	92,7	96,9	101,2
65,2	57,2	57,6	61,6	55,2	55	60,4	60,5	59,9	55,9	60,3	58,2
73,2	85,9	81,6	76,3	79,7	79,6	82,7	75,6	78,2	84,4	84,4	80,5
87	73,4	72,7	73,2	70,6	82,3	74,8	77,9	85,5	89,9	78,2	76
89,6	74,4	65,2	72,4	64,6	69,5	77,5	85	100,7	94,6	97,8	103,9
100,4	109,5	105,5	110,8	106,3	107	104	96,8	98,4	91,3	89,5	79,3
100,8	104,9	96,8	95,7	110,7	101,6	93,1	93,3	87,4	106,4	96,4	96,1
78,6	75,6	69,7	93,3	93,3	83,3	74,2	78,1	73,3	79,7	75,1	77
82,2	66,9	70,1	71,2	58,7	62,9	76,1	74	63,8	69,5	61,2	62,3
90,4	90,7	91,9	89,6	104,9	85,8	90,4	101,6	92,1	89,5	80,1	90
111,2	103,4	115,6	110,4	101,1	113,3	92,5	109,2	103,1	116,9	110,3	95,6
72,9	60,8	64,3	60,2	60,5	61,1	62,8	59,2	63,6	57	58	65,5
80,4	91,3	80,7	84,9	87,2	96,3	84,3	77,2	80,2	88,2	96,9	86,3
84,3	73,1	97,1	82,1	83	84,2	82,7	85	99,3	93,8	86,3	80,3
71,9	61,4	83,2	67,7	64	65,7	68,1	68,4	77,8	67,4	65,5	68,6
75,7	72,8	66,3	68,6	66,9	76,3	80,7	83,8	81,2	73,6	64,9	71,3
71,5	74,2	77,3	82	80,5	69,7	71,9	93,8	81,6	73,8	79,3	70,2
82,8	67,5	74,4	72,9	73,8	77,6	88,4	76,1	77,7	80,1	77,8	73,4
55	69,8	66,6	68,5	65,5	57,9	78,8	71,6	60,9	111,1	71,8	71,3
91	90,1	83,5	89,1	86,8	94,9	90,9	80,3	88	96,2	88,2	80,7
89,8	93,3	101,7	78,7	81,4	81,7	81	79,4	84,9	88,7	83,4	87,5
71,4	73,9	77,1	81	79,8	74	80,6	70,4	82,5	71,8	81,9	67,7
82,6	85,7	80,7	86,2	78,1	72,7	83,7	84,2	99,7	81,1	88,6	86,4
95,4	96,5	109	97,1	105,7	91,4	97,2	113	95,9	101,6	108,5	92,6
74,7	73	76,2	77,9	84,5	77,6	77,6	82,9	76,1	77,8	79,6	81,7
44,9	37,2	38,8	45,4	43,3	35,5	34,9	41,7	45,2	39,8	35,7	47,4
67,5	80,6	66	72,3	63,4	75,9	54,9	74,7	74,6	66,1	53,4	51,8
85,9	74,1	70	87,6	85,6	73,2	83,3	80,1	84,1	84,3	76,3	84,1
94	99	85,4	87,1	94,7	93,4	78,3	79	86	89,4	102,3	106
102,7	103,7	119,9	93,3	96,9	111	108	91,1	102,2	92,9	93,6	90,6
81	75,2	76,3	73,7	76,2	78,3	74,7	80,2	81,1	73	79,8	80,7

54,8	51,9	52,2	54,7	55,6	64,5	49,6	49,1	57,3	59,2	58,2	69,7
