# Supplementary material for: Kinetic gait analysis in healthy dogs and dogs with osteoarthritis: An evaluation of precision and overlap performance of a pressure-sensitive walkway and the use of symmetry indices
Source: PLoS One. 2020 Dec 15;15(12):e0243819. doi: 10.1371/journal.pone.0243819 (PMC7737891; doi:10.1371/journal.pone.0243819)
Supplement: S5 File — The calculated means and standard deviation listed in Table 2 are based on 6 walkway measurements of each limb of 21 dogs with osteoarthritis. Temporal characteristics and measured ground reaction forces are listed in the present file. (PDF) [file pone.0243819.s005.pdf]

S5 File. Temporal characteristics and ground reaction forces measured in dogs with osteoarthritis

The calculated means and standard deviation listed in Table 2 are based on 6 walkway measurements of each limb of 21 dogs with osteoarthritis. Temporal characteristics and measured ground reaction forces are listed in the present file.

Abbreviations:

RF	Measured ground reaction forces in 6 recordings of right thoracic limb
RH	Measured ground reaction forces in 6 recordings of right pelvic limb
LF	Measured ground reaction forces in 6 recordings of left thoracic limb
LH	Measured ground reaction forces in 6 recordings of left pelvic limb

Stance time (sec)

**LF**						**RF**					
0,41	0,47	0,46	0,5	0,52	0,51	0,44	0,46	0,46	0,49	0,49	0,48
0,44	0,5	0,47	0,48	0,48	0,51	0,47	0,53	0,51	0,51	0,54	0,51
0,57	0,6	0,54	0,58	0,6	0,59	0,53	0,57	0,55	0,58	0,56	0,6
0,52	0,54	0,56	0,54	0,54	0,59	0,53	0,55	0,56	0,54	0,54	0,56
0,5	0,52	0,46	0,47	0,46	0,48	0,5	0,52	0,48	0,51	0,48	0,5
0,47	0,49	0,51	0,52	0,49	0,51	0,47	0,48	0,49	0,51	0,5	0,52
0,49	0,53	0,54	0,53	0,49	0,47	0,48	0,52	0,54	0,52	0,5	0,49
0,43	0,51	0,5	0,46	0,47	0,48	0,43	0,5	0,48	0,47	0,49	0,46
0,43	0,44	0,45	0,44	0,44	0,41	0,43	0,45	0,46	0,44	0,42	0,42
0,5	0,48	0,5	0,49	0,52	0,54	0,51	0,47	0,51	0,5	0,51	0,54
0,55	0,56	0,56	0,57	0,55	0,58	0,55	0,57	0,54	0,55	0,57	0,54
0,45	0,45	0,41	0,48	0,48	0,45	0,45	0,47	0,42	0,47	0,47	0,46
0,45	0,47	0,49	0,49	0,47	0,47	0,44	0,46	0,48	0,49	0,47	0,47
0,54	0,53	0,43	0,56	0,55	0,51	0,54	0,51	0,45	0,56	0,57	0,53
0,42	0,43	0,5	0,54	0,52	0,55	0,45	0,47	0,51	0,55	0,59	0,61
0,54	0,66	0,65	0,68	0,71	0,69	0,6	0,69	0,68	0,68	0,7	0,67
0,57	0,55	0,59	0,57	0,59	0,63	0,57	0,56	0,58	0,58	0,58	0,61
0,54	0,57	0,58	0,57	0,55	0,57	0,55	0,55	0,6	0,57	0,56	0,6
0,49	0,46	0,49	0,54	0,51	0,53	0,49	0,49	0,5	0,51	0,48	0,49

0,58	0,54	0,59	0,59	0,58	0,56	0,58	0,6	0,62	0,61	0,65	0,59
0,55	0,52	0,55	0,55	0,57	0,56	0,56	0,52	0,56	0,55	0,55	0,54

**LH**						**RH**					
0,39	0,42	0,42	0,43	0,45	0,42	0,41	0,43	0,44	0,44	0,46	0,42
0,43	0,49	0,45	0,47	0,48	0,48	0,44	0,49	0,46	0,48	0,48	0,49
0,56	0,58	0,52	0,56	0,55	0,56	0,56	0,59	0,53	0,57	0,56	0,57
0,55	0,53	0,55	0,53	0,52	0,54	0,51	0,54	0,54	0,51	0,49	0,52
0,49	0,53	0,46	0,47	0,44	0,48	0,47	0,51	0,45	0,48	0,45	0,47
0,48	0,5	0,5	0,52	0,49	0,5	0,49	0,5	0,49	0,51	0,5	0,5
0,47	0,5	0,5	0,52	0,49	0,47	0,47	0,49	0,52	0,52	0,47	0,45
0,42	0,47	0,48	0,44	0,45	0,45	0,41	0,47	0,47	0,42	0,45	0,43
0,63	0,69	0,73	0,67	0,66	0,67	0,65	0,67	0,67	0,71	0,61	0,67
0,51	0,47	0,49	0,49	0,5	0,53	0,47	0,46	0,48	0,48	0,48	0,5
0,55	0,54	0,52	0,56	0,57	0,53	0,55	0,56	0,53	0,54	0,57	0,56
0,41	0,41	0,38	0,43	0,42	0,41	0,39	0,41	0,37	0,41	0,43	0,41
0,34	0,43	0,31	0,46	0,29	0,43	0,39	0,46	0,25	0,47	0,35	0,45
0,5	0,51	0,46	0,54	0,53	0,49	0,52	0,51	0,45	0,52	0,53	0,47
0,44	0,44	0,54	0,53	0,55	0,56	0,47	0,45	0,53	0,55	0,57	0,59
0,55	0,66	0,65	0,64	0,64	0,63	0,52	0,66	0,7	0,7	0,7	0,66
0,53	0,57	0,56	0,55	0,55	0,6	0,54	0,57	0,55	0,55	0,56	0,59
0,55	0,55	0,57	0,52	0,51	0,52	0,51	0,57	0,55	0,52	0,5	0,55
0,59	0,59	0,6	0,56	0,58	0,58	0,59	0,57	0,57	0,57	0,54	0,55
0,58	0,51	0,57	0,55	0,66	0,57	0,58	0,6	0,62	0,52	0,6	0,52
0,54	0,5	0,56	0,53	0,53	0,52	0,51	0,48	0,55	0,48	0,55	0,52

Swing time (sec)

**LF**						**RF**					
0,29	0,28	0,29	0,26	0,26	0,28	0,28	0,29	0,28	0,28	0,29	0,28
0,27	0,28	0,28	0,28	0,32	0,26	0,22	0,25	0,24	0,26	0,24	0,25
0,3	0,28	0,32	0,32	0,3	0,3	0,3	0,3	0,31	0,32	0,3	0,32
0,28	0,3	0,32	0,32	0,3	0,28	0,34	0,28	0,32	0,3	0,3	0,32

0,3	0,28	0,28	0,3	0,28	0,28	0,28	0,27	0,24	0,26	0,26	0,25
0,22	0,24	0,25	0,24	0,26	0,24	0,24	0,24	0,26	0,25	0,24	0,24
0,26	0,28	0,28	0,28	0,28	0,28	0,28	0,3	0,29	0,3	0,28	0,27
0,26	0,26	0,26	0,28	0,28	0,27	0,27	0,28	0,26	0,29	0,28	0,28
0,25	0,26	0,22	0,23	0,24	0,24	0,25	0,25	0,25	0,24	0,22	0,25
0,28	0,28	0,26	0,27	0,28	0,28	0,28	0,28	0,27	0,26	0,3	0,29
0,26	0,28	0,27	0,28	0,28	0,28	0,3	0,26	0,32	0,31	0,28	0,34
0,23	0,24	0,24	0,24	0,25	0,26	0,23	0,25	0,22	0,25	0,26	0,24
0,27	0,29	0,28	0,29	0,3	0,3	0,26	0,28	0,28	0,3	0,3	0,34
0,34	0,32	0,33	0,3	0,32	0,32	0,32	0,32	0,32	0,3	0,3	0,32
0,2	0,22	0,21	0,2	0,23	0,24	0,18	0,18	0,19	0,19	0,19	0,19
	0,34	0,34	0,34	0,34	0,32	0,32	0,34	0,36	0,34	0,32	0,32
0,3	0,32	0,3	0,32	0,3	0,32	0,34	0,32	0,34	0,32	0,32	0,32
0,3	0,3	0,32	0,32	0,3	0,34	0,32	0,3	0,3	0,31	0,3	0,3
0,3	0,28	0,31	0,29	0,3	0,28	0,31	0,27	0,32	0,32	0,31	0,3
0,36	0,34	0,32	0,32	0,32	0,36	0,3	0,3	0,26	0,3	0,29	0,31
0,29	0,29	0,3	0,26	0,28	0,3	0,26	0,3	0,29	0,28	0,3	0,3

**LH**						**RH**					
0,33	0,34	0,34	0,33	0,34	0,32	0,32	0,34	0,32	0,32	0,33	0,32
0,29	0,3	0,3	0,29	0,31	0,3	0,28	0,31	0,28	0,28	0,31	0,29
0,34	0,32	0,32	0,34	0,35	0,36	0,34	0,31	0,32	0,32	0,36	0,32
0,3	0,34	0,34	0,32	0,32	0,34	0,34	0,32	0,34	0,34	0,35	0,36
0,31	0,3	0,3	0,32	0,32	0,3	0,32	0,33	0,32	0,32	0,3	0,3
0,24	0,24	0,26	0,24	0,25	0,26	0,24	0,24	0,26	0,26	0,25	0,26
0,3	0,3	0,34	0,31	0,3	0,3	0,28	0,32	0,32	0,32	0,31	0,3
0,3	0,3	0,3	0,32	0,32	0,3	0,3	0,3	0,3	0,32	0,34	0,32
0,37	0,34	0,36	0,43	0,36	0,42	0,32	0,38	0,4	0,4	0,36	0,37
0,28	0,3	0,3	0,29	0,32	0,3	0,32	0,3	0,3	0,32	0,32	0,32
0,28	0,3	0,33	0,3	0,27	0,32	0,3	0,28	0,32	0,32	0,28	0,32
0,28	0,3	0,26	0,28	0,3	0,3	0,29	0,29	0,29	0,31	0,3	0,3

0,34	0,34	0,2	0,34	0,26	0,34	0,24	0,32	0,21	0,32	0,21	0,32
0,34	0,32	0,3	0,34	0,34	0,34	0,34	0,34	0,32	0,34	0,36	0,34
0,18	0,19	0,19	0,19	0,18	0,2	0,16	0,19	0,16	0,18	0,18	0,18
0,34	0,38	0,4	0,38	0,4	0,38	0,3	0,36	0,38	0,34	0,34	0,34
0,36	0,32	0,3	0,36	0,36	0,36	0,32	0,34	0,34	0,34	0,34	0,34
0,32	0,35	0,36	0,34	0,34	0,36	0,36	0,34	0,36	0,36	0,34	0,34
0,26	0,22	0,28	0,27	0,26	0,24	0,27	0,27	0,3	0,24	0,29	0,27
0,38	0,4	0,42	0,32	0,36	0,32	0,34	0,32	0,34	0,34	0,4	0,36
0,3	0,32	0,32	0,31	0,32	0,35	0,34	0,34	0,33	0,34	0,34	0,36

Stride time (sec)

**LF**						**LH**					
0,68	0,73	0,75	0,74	0,74	0,74	0,7	0,73	0,76	0,76	0,74	0,76
0,74	0,81	0,82	0,8	0,76	0,74	0,77	0,8	0,84	0,84	0,78	0,76
0,68	0,78	0,76	0,74	0,74	0,75	0,72	0,76	0,75	0,74	0,76	0,74
0,68	0,66	0,67	0,68	0,62	0,67	0,98	0,92	1,1	1,04	0,96	1,06
0,78	0,74	0,74	0,75	0,79	0,82	0,76	0,75	0,78	0,78	0,8	0,81
0,8	0,84	0,82	0,84	0,84	0,86	0,82	0,82	0,85	0,86	0,84	0,86
0,68	0,7	0,66	0,72	0,73	0,7	0,7	0,71	0,64	0,7	0,72	0,71
0,71	0,75	0,76	0,78	0,78	0,77	0,76	0,78	0,47	0,8	0,47	0,77
0,9	0,86	0,76	0,88	0,88	0,84	0,86	0,86	0,76	0,86	0,88	0,84
0,62	0,66	0,71	0,74	0,74	0,81	0,61	0,64	0,73	0,75	0,75	0,79
	0,98	0,96	1,02	1,08	1,04	0,96	1,04	1,02	1,02	1,06	1,02
0,69	0,72	0,73	0,76	0,8	0,82	0,72	0,75	0,76	0,77	0,8	0,76
0,7	0,78	0,76	0,75	0,8	0,76	0,72	0,78	0,75	0,74	0,79	0,78
0,84	0,82	0,84	0,9	0,88	0,9	0,88	0,92	0,84	0,9	0,91	0,92
0,74	0,84	0,9	0,86	0,82	0,88	0,86	0,86	0,86	0,86	0,84	0,9
0,78	0,78	0,73	0,75	0,72	0,74	0,79	0,82	0,75	0,78	0,76	0,76
0,9	0,86	0,88	0,9	0,88	0,96	0,88	0,88	0,88	0,9	0,92	0,94
0,82	0,84	0,9	0,9	0,86	0,92	0,84	0,87	0,92	0,86	0,84	0,88
0,76	0,7	0,77	0,83	0,8	0,8	0,8	0,72	0,84	0,82	0,84	0,8
0,9	0,87	0,9	0,96	0,86	0,94	0,94	0,96	0,98	0,88	1	0,9

0,82	0,8	0,84	0,82	0,83	0,84	0,82	0,82	0,84	0,83	0,84	0,85

**RF**						**RH**					
0,7	0,72	0,75	0,75	0,73	0,76	0,71	0,72	0,76	0,76	0,75	0,76
0,76	0,82	0,83	0,8	0,78	0,75	0,74	0,81	0,86	0,84	0,78	0,74
0,69	0,78	0,74	0,75	0,76	0,74	0,71	0,77	0,74	0,72	0,77	0,76
0,64	0,69	0,68	0,63	0,61	0,71	0,92	1,03	1,02	1,02	0,96	1,03
0,78	0,74	0,77	0,74	0,8	0,82	0,79	0,76	0,78	0,8	0,8	0,82
0,85	0,82	0,86	0,87	0,86	0,88	0,84	0,84	0,84	0,86	0,84	0,88
0,68	0,71	0,64	0,72	0,72	0,7	0,68	0,71	0,66	0,71	0,72	0,7
0,72	0,74	0,76	0,76	0,76	0,8	0,67	0,78	0,39	0,79	0,51	0,76
0,88	0,84	0,78	0,86	0,86	0,86	0,88	0,86	0,78	0,86	0,88	0,84
0,62	0,65	0,69	0,75	0,75	0,81	0,62	0,65	0,71	0,75	0,77	0,79
1	1,02	1,02	1,04	1,04	0,98	0,82	1,02	1,06	1,06	1,04	0,98
0,7	0,73	0,72	0,8	0,78	0,78	0,72	0,76	0,75	0,76	0,78	0,76
0,68	0,77	0,74	0,78	0,78	0,74	0,72	0,79	0,74	0,74	0,79	0,78
0,8	0,85	0,86	0,9	0,84	0,92	0,88	0,9	0,84	0,9	0,92	0,86
0,86	0,84	0,9	0,86	0,84	0,9	0,84	0,84	0,86	0,84	0,85	0,88
0,76	0,79	0,72	0,76	0,74	0,75	0,8	0,83	0,76	0,8	0,74	0,76
0,92	0,9	0,92	0,88	0,9	0,92	0,86	0,88	0,88	0,9	0,88	0,92
0,84	0,82	0,9	0,87	0,86	0,9	0,86	0,86	0,88	0,88	0,84	0,9
0,77	0,71	0,78	0,82	0,79	0,77	0,82	0,81	0,84	0,8	0,83	0,8
0,82	0,88	0,84	0,93	0,88	0,9	0,9	0,92	0,9	0,86	0,94	0,88
0,8	0,8	0,84	0,82	0,86	0,8	0,82	0,81	0,85	0,82	0,84	0,88

Stride length (cm)

**LF**						**RF**					
74,2	75,2	75,2	69,1	73,2	75,2	76,2	76,7	75,2	71,1	73,7	74,2
72,1	69,1	72,1	70,1	72,1	69,1	72,1	70,1	71,1	71,1	70,6	68,6
87,4	75,2	84,3	87,4	88,4	87,4	86,4	79,2	85,9	87,4	87,4	84,3
81,3	83,3	84,3	82,3	84,3	82,3	83,3	84,3	83,3	84,3	84,3	82,3
75,2	76,2	75,7	73,7	77,7	76,2	75,2	75,7	76,2	75,2	78,2	75,7

71,1	71,1	70,1	67,1	71,1	68,6	71,1	74,2	71,1	69,1	71,1	69,1
77,2	76,2	73,2	75,7	81,3	80,3	76,7	76,2	77,7	77,2	81,3	80,8
75,2	69,1	73,2	80,3	77,2	79,2	75,2	72,1	75,2	80,3	80,3	78,2
62	55,4	52,3	55,5	51,3	56,2	59,4	56,9	54,5	56,4	47,8	63,5
76,2	80,3	76,2	76,2	76,2	74,2	75,2	82,3	78,2	77,2	77,2	75,7
81,3	80,3	82,8	82,3	84,3	82,3	82,3	83,3	83,3	83,3	81,3	83,3
69,6	71,1	72,1	71,1	72,1	72,1	70,1	71,6	71,1	70,1	73,2	71,1
69,1	74,2	75,2	71,6	76,2	73,2	67,1	74,2	74,2	71,1	75,7	74,2
82,3	84,3	81,8	79,2	81,3	81,8	82,3	82,8	79,2	79,2	77,7	83,3
52,2	54,4	45,7	44,2	46,7	47,8	53,3	52,5	47,1	47,1	47,1	47,4
	93,5	96,5	96,5	98,6	100,6	103,6	97,5	99,6	95,5	94,5	99,6
85,3	85,3	81,3	91,4	92,5	87,4	87,4	84,3	86,4	90,4	88,9	87,4
87,4	82,3	79,2	86,4	87,4	84,3	87,4	86,4	81,3	83,8	86,4	85,3
77,2	75,2	79,2	74,7	72,1	73,2	76,7	74,7	79,2	76,2	72,1	74,7
86,4	86,9	86,4	84,3	87,4	83,3	89,4	89,4	89,4	85,9	83,3	86,9
74,2	76,2	77,2	75,2	74,7	77,2	73,2	76,2	76,2	75,7	75,2	77,2

**LH**						**RH**					
72,6	74,2	74,2	73,7	73,2	76,2	74,2	76,2	75,2	74,2	73,7	74,2
70,6	69,1	70,6	70,1	71,1	68,1	70,1	69,6	69,1	70,1	70,1	69,1
79,2	72,1	80,3	86,4	87,4	86,4	88,4	77,2	85,3	87,4	89,4	88,4
75,2	83,3	84,3	83,3	84,3	83,3	80,8	84,3	86,4	82,3	83,8	83,3
75,2	71,1	75,2	73,2	78,2	73,2	77,2	75,2	75,2	73,2	76,2	74,7
68,1	70,6	69,1	67,1	70,1	68,1	67,6	70,1	70,1	68,1	69,6	69,1
76,7	76,2	80,3	78,2	78,2	82,3	76,2	75,2	78,2	79,2	79,2	80,3
75,2	71,1	72,1	80,3	77,2	80,3	75,2	71,1	72,1	80,3	77,7	80,3
81,8	79,2	78,2	80,3	81,3	83,3	75,2	78,2	78,2	85,3	83,3	82,3
75,2	77,2	76,2	74,7	73,2	72,6	74,7	78,2	77,2	74,2	75,7	75,2
83,3	83,3	83,3	83,3	81,8	81,3	83,8	82,3	84,3	81,3	83,3	82,3
70,1	71,6	71,1	70,1	71,1	71,6	70,1	70,6	72,6	69,1	72,1	72,6
75,7	72,6	48,4	71,6	54,9	73,7	59,6	72,1	37,3	70,6	49,8	71,1
82,3	82,3	80,3	79,2	78,2	82,3	81,3	81,3	81,3	78,2	79,2	85,3

52,2	53,5	47,8	47,4	47,8	48,1	52,3	54,4	46,5	47,1	48,4	47,4
112,8	97,5	95,5	96,5	97,5	99,6	112,8	92,5	96,5	94,5	94,5	99,6
87,4	86,4	85,3	89,4	88,4	84,3	86,4	82,3	84,3	89,4	88,4	85,3
82,3	80,3	79,2	84,3	86,4	84,3	84,3	81,3	80,3	84,3	87,4	84,3
71,1	64	68,1	72,6	75,2	72,1	72,6	70,1	72,1	72,1	73,2	72,1
84,3	87,4	82,3	90,4	77,2	86,4	84,3	86,4	84,3	90,4	83,3	85,3
72,1	74,2	73,2	75,2	75,2	75,2	73,2	75,7	73,2	76,2	73,2	74,2

Stride velocity (cm/sec)

**LF**						**RF**					
107,5	104,4	103	90,9	91,4	91,7	108,9	105,1	104,4	88,9	94,4	95,1
103,1	88,6	94,9	93,5	90,2	90,9	106,1	91	96,1	91,2	90,5	92,7
104	91,7	100,4	97,1	100,4	97,1	108	93,2	99,8	97,1	104	91,7
109,8	99,2	93,7	95,7	102,8	93,5	96,9	100,4	92,6	98,1	100,4	91,4
96,4	97,7	103,7	98,2	108	103	98,9	95,8	105,8	98,9	105,7	100,9
104,6	97,4	93,5	90,6	96,1	92,7	101,6	103	94,8	92,1	97,4	90,9
104,3	94,1	89,2	94,6	106,9	108,5	100,9	92,9	93,6	96,5	104,2	107,7
110,6	88,6	96,3	108,5	104,3	105,7	109	92,5	101,6	107	105,6	105,7
91,1	83,9	78,1	81,7	82,8	83,5	92,9	82,9	80,2	89,5	78,3	89,4
97,7	108,5	103	101,6	96,5	90,4	96,4	111,2	101,6	104,3	96,5	92,3
101,6	95,6	101	98	100,4	95,7	96,8	101,6	96,9	95,8	94,5	94,7
102,3	101,6	109,3	98,8	98,8	103,1	103,1	100,9	111,1	97,4	101,6	101,6
97,3	98,9	98,9	91,8	97,7	95	93,1	100,2	97,6	93,6	99,6	92,7
91,4	98,1	107,6	90,1	92,4	97,4	93,5	98,6	101,6	92,1	90,4	96,9
84,1	82,4	64,7	59,7	63,2	59,2	86	81,2	67,9	63	63	58,3
	95,4	100,5	94,6	91,3	96,7	103,6	95,6	97,6	91,8	90,9	101,6
94,8	99,2	92,4	101,6	105,1	91	95	93,7	93,9	102,8	98,8	95
106,6	98	88,1	96	101,6	91,7	104	105,3	90,3	96,3	100,4	94,8
101,6	107,4	102,9	90	90,2	91,4	99,6	105,2	101,6	92,9	91,3	97
96	99,8	96	87,8	101,6	88,6	109	101,6	106,4	92,3	94,7	96,5
90,4	95,3	91,9	91,7	90	91,9	91,4	95,3	90,7	92,3	87,4	96,5

LH						RH					
100,9	98,9	97,6	95,7	91,4	100,3	103	100,3	100,2	97,6	94,4	97,6
98,1	88,6	94,1	94,7	90	87,3	97,4	88,1	93,4	94,7	88,7	88,6
90,1	78,4	95,6	96	96	93,9	100,4	85,8	101,6	97,1	97,2	102,8
87,4	96,9	98,1	96,9	100,4	92,6	96,2	100,4	100,4	98	98,6	94,7
95,2	86,7	100,2	93,8	102,9	96,3	96,5	90,6	98,9	91,4	103	98,3
97,2	96,7	90,9	88,2	94,7	89,6	95,2	97,4	92,2	89,6	92,8	90,9
99,6	95,3	95,6	93,1	100,3	108,3	103	92,8	91	94,3	101,6	108,5
104,4	93,6	96,2	108,5	101,6	108,5	105,9	92,4	97,5	111,5	100,9	105,6
83,5	86,1	71,1	77,2	84,7	78,6	81,7	76	76,7	83,7	86,8	79,9
98,9	103	97,7	95,7	91,4	89,7	94,5	102,9	99	92,7	94,6	91,7
101,6	101,6	98	96,9	97,4	94,5	99,8	98	100,4	94,5	99,2	93,5
100,1	100,9	111,1	100,1	98,8	100,9	103,1	99,5	110,1	97,3	100,2	103,8
99,6	93,1	103,8	89,5	116,7	95,7	88,5	92,5	96,3	89,4	97	93,6
95,7	95,7	105,6	92,1	88,9	98	92,4	94,5	104,2	91	90,1	101,6
85	83,6	65,1	63,5	63,7	61,1	84,4	83,6	65	62,5	63,2	59,8
117,5	93,8	93,6	94,6	92	97,6	137,5	90,6	91,1	89,1	90,9	101,6
99,3	98,1	97	99,3	96,1	89,7	100,4	93,5	95,8	99,3	100,4	92,8
98	92,3	86,1	98,1	102,8	95,8	98,1	94,5	91,2	95,8	104	93,7
88,9	88,9	81	88,6	89,5	90,2	88,6	86,5	85,9	90,2	88,1	90,2
89,7	91	84	102,8	77,2	96	93,7	93,9	93,7	105,1	88,6	97
88	90,4	87,1	90,6	89,5	88,5	89,2	93,4	86,1	92,9	87,1	84,3

Peak vertical force (N)

LF						RF					
60,1	54,8	60,8	64,4	57	61,3	62,4	65,4	79,2	61,2	57,1	67,2
46,8	46,6	48,9	42,3	41,9	43,4	52,1	58,8	54,8	58,2	57,4	56,7
49,1	52,5	57,5	46,6	44	55,8	48,5	51,6	55,5	51	52,7	49,4
58,1	52,7	53,3	56,4	55,6	55,4	56,6	70,8	60,8	55	61,4	53,3
51,2	58,9	60,4	54,4	56,9	65,3	56	55,8	59,8	59,5	59,3	59,8
49,4	45,9	51,5	44,8	44,8	43,6	44,7	48,6	46,2	46	45,1	48
570,9	581	585,9	521,3	527,8	561,9	496,4	418,5	462,1	420	443,3	502,2

55,3	44,4	51,4	59,5	48,5	48,2	52	48,1	45,8	51,3	49,6	56,3
71,4	70	68,8	62,7	67,2	65,2	57,2	61	57,5	69,7	59,7	67,2
54,1	54,1	59,9	47,4	48,1	55	48,1	45	47,6	55,7	57,3	51,6
51,3	54,6	45,8	46,1	44,6	43,4	27,2	51,2	29,4	26,9	52,7	34,5
62,4	53,8	56,3	49,2	51,9	53,2	52	51,3	59,6	47,4	59,9	53,1
65,8	58,9	61	57,7	55,5	51,1	56,4	50,4	59,2	49,4	58,4	55,4
58,9	63,4	59,7	52,2	54,7	58	58,6	53,4	58,6	51,4	54,7	56,7
36,4	38,7	42,9	36,2	30,9	32,7	46,4	50,4	36,9	43,6	45,3	42,8
56,3	60	56,7	60	59,4	60,9	51,6	57,4	62,5	56,4	59,7	56,8
48,6	50,3	47	45	56,3	44,5	42,8	50,2	47,8	51,5	52,6	47,3
56	51,7	57,8	54,5	63,5	47,3	51,7	51,3	50,4	56,3	54,7	53,2
61,6	64,7	60,3	54,3	62,9	64,1	59	51,7	57,4	55,6	56,5	57,6
55,8	64,2	53,4	58,3	61,7	61,7	54,7	63,3	59,5	62,9	53,2	60,3
53,7	52,4	49,5	52,1	50,2	50,3	55,6	53,3	53,7	51,2	50,7	46,5

**LH**						**RH**					
34,7	37,1	34,6	35,9	32	43,3	32,6	36	34,1	38,2	31,2	34
28,7	31,5	30,5	32,2	31,6	28,9	34,2	30,1	33,9	31,2	33,3	33,7
26,2	34,7	27,2	27,9	30,5	26,1	30,8	29,2	30,7	30,8	30,7	27,7
28,9	29,8	25,5	38,4	32,9	27,2	28,1	34,4	26,3	28,9	33,6	24,4
32,2	37,1	34,5	32,1	41,3	31,9	30,5	26,5	33,3	33,6	33,6	28,9
32,6	29,6	29,6	25,5	28,7	29,9	29,3	27,7	33,4	30,1	29,6	27,9
300,9	314,8	286,3	302,7	273,8	265	348,4	266,7	331,3	296,1	302,1	310,3
30,9	27,3	27,2	31,1	32,1	30,5	30,1	27,9	30,6	27,7	31,2	30,4
40,2	39,6	34,7	37	31,8	36,9	39,8	37,4	34,3	37,8	38,2	36,3
36,1	36,2	34,7	34,9	36,6	34,9	32,6	35,1	35,8	32,9	37,7	38,3
32	53,4	28,4	28,9	50,4	23,6	28,5	47,8	35,8	27,7	49,6	28,4
32,6	34,3	35,3	30,5	33,3	34,1	37,6	36,9	38,6	31,5	30,3	33,6
43,6	39,5	26,7	41	31,4	43,1	39	42,1	39,9	38,7	36	44,4
28,5	26,9	27,8	27,1	29	25	24,9	26,2	32,4	27,9	29,7	31,1
26	26	27,1	25,6	27,7	23,9	48,2	40,2	36,9	37,4	38,1	40
36,9	28,3	30	25,2	25	26,4	35,5	27,3	33,1	28,7	29,3	30,2

29,8	27,6	29,8	26,6	26,8	25	33,5	26	30,7	30,6	23,6	26,3
39,8	38,2	32,8	29,2	30,8	30,6	38,3	34,4	30,6	36,6	38,8	40,9
31,4	33,8	35,3	31,3	32,6	30,8	28,2	27,4	31,2	32,8	30,2	28,5
25,6	23	23,2	28,8	25,6	26,6	27,3	31,2	26,8	22,3	25,2	23
33,6	30,5	28,3	28,8	29,5	31,7	26,7	26,3	24,7	28,1	24,5	26,4

Peak vertical force (%BW)

**LF**						**RF**					
185,532	169,154	187,707	198,968	176,05	189,255	192,781	202,022	244,796	189,167	176,313	207,498
160,76	160,067	167,828	145,31	143,819	149,068	178,888	201,768	188,238	199,609	197,076	194,512
207,113	221,388	242,385	196,496	185,495	235,425	204,636	217,577	233,848	214,91	222,254	208,197
205,034	185,906	188,086	199,137	196,375	195,555	199,942	249,98	214,649	194,278	216,624	188,005
155,741	178,917	183,542	165,259	173,033	198,407	170,353	169,648	181,867	180,857	180,131	181,779
227,55	211,421	237,244	206,412	206,499	201,146	205,847	224,137	213,137	211,793	208,076	221,015
171,269	174,291	175,758	156,383	158,352	168,564	148,933	125,551	138,632	126,008	132,978	150,67
177,897	142,762	165,275	191,521	156,119	154,931	167,169	154,709	147,406	165,036	159,468	181,051
279,448	274,013	269,053	245,406	263,131	255,231	223,917	238,585	224,968	272,759	233,555	263,102
159,068	159,118	176,211	139,336	141,567	161,924	141,364	132,448	139,935	163,85	168,522	151,733
201,71	214,626	180,098	181,441	175,225	170,701	107,062	201,187	115,519	105,776	207,114	135,481
165,27	142,453	149,066	130,365	137,414	140,868	137,605	135,9	157,83	125,59	158,625	140,683
175,425	157,137	162,719	153,966	147,932	136,262	150,402	134,506	157,997	131,866	155,9	147,683
196,3	211,491	199,101	174,133	182,485	193,495	195,268	178,009	195,282	171,544	182,479	189,023
121,435	128,982	143,184	120,753	103,189	109,097	154,75	168,165	123,133	145,31	151,002	142,673
267,637	285,202	269,601	285,306	282,487	289,455	245,196	273,188	297,065	268,486	284,107	270,072
171,707	177,581	165,977	158,946	198,856	156,949	151,229	177,07	168,774	181,674	185,726	167,115
191,243	176,29	197,377	185,827	216,733	161,49	176,283	175,171	172,122	192,001	186,606	181,547
265,892	279,158	260,189	234,33	271,295	276,763	254,702	222,912	247,569	240,122	243,61	248,625
218,978	251,982	209,639	228,611	241,93	241,948	214,737	248,273	233,257	246,749	208,748	236,589
205,245	200,256	189,194	199,23	192,153	192,536	212,594	203,971	205,424	195,767	194,029	177,773

**LH**						**RH**					
107,075	114,72	106,956	110,886	98,866	133,846	100,631	111,172	105,434	117,966	96,305	105,126

98,646	108,248	104,818	110,439	108,297	99,03	117,536	103,143	116,332	107,148	114,342	115,789
110,645	146,302	114,787	117,798	128,802	109,922	129,952	123,138	129,39	129,786	129,397	116,868
102,102	105,294	90,165	135,705	115,996	95,942	99,232	121,576	92,844	101,977	118,777	86,043
97,818	112,741	104,743	97,438	125,428	97,033	92,701	80,48	101,381	102,027	102,192	87,765
150,08	136,244	136,31	117,611	132,366	137,832	135,111	127,774	153,723	138,642	136,648	128,592
90,281	94,431	85,897	90,814	82,147	79,504	104,512	79,999	99,403	88,829	90,631	93,09
99,474	87,926	87,519	100,169	103,183	98,234	96,945	89,793	98,466	89,179	100,465	97,713
157,156	155,144	135,58	144,629	124,554	144,478	155,792	146,262	134,136	147,966	149,561	141,88
106,229	106,574	102,125	102,592	107,755	102,721	96,008	103,326	105,291	96,646	110,884	112,532
125,864	209,799	111,668	113,784	198,316	92,717	112,186	188,055	140,802	109,013	194,869	111,614
86,302	90,864	93,378	80,728	88,097	90,259	99,634	97,721	102,101	83,413	80,207	88,891
116,399	105,301	71,156	109,48	83,759	114,888	103,905	112,408	106,505	103,098	96,081	118,526
95,014	89,637	92,714	90,486	96,857	83,309	83,02	87,368	107,991	93,169	99,107	103,805
86,685	86,678	90,311	85,469	92,424	79,579	160,706	134,153	123,112	124,665	126,95	133,261
175,563	134,609	142,746	119,908	118,679	125,6	168,635	129,701	157,484	136,279	139,187	143,805
105,241	97,419	105,188	93,743	94,487	88,089	118,438	91,96	108,476	108,037	83,295	92,996
135,814	130,288	111,904	99,814	105,099	104,352	130,692	117,371	104,321	124,868	132,331	139,539
135,584	146,046	152,129	135,183	140,698	132,961	121,511	118,442	134,828	141,651	130,346	123,16
100,368	90,357	91,2	113,141	100,346	104,277	107,02	122,378	105,233	87,299	99,017	90,169
128,413	116,468	108,244	109,964	112,948	121,417	102,181	100,737	94,562	107,614	93,787	101,053

Vertical impulse (%BW*sec)

**LF**						**RF**					
17,1	17,7	19,6	21,5	19,9	22,1	20,5	22,2	27,3	21,7	20,8	22,7
14,2	16,5	16	14,8	14,6	15,7	18,9	22,8	21,2	22	22,7	22,7
20,6	23,8	23	19,8	19,2	24,4	20	20,7	23,1	22,3	21,2	21,6
21,1	19,6	21	21,5	20,5	23,5	21,5	25,5	22,6	20,2	22,4	19,6
17,6	19,9	18,3	17,3	17,6	20	20,8	22,1	21,1	22,7	21,4	21,7
17	16,3	18,9	16,8	16	16	15,1	16,8	16,4	16,8	16,2	17,3
208,6	234,3	236,7	208,9	200,2	195	165,2	150,8	169,6	149,2	148,6	163,1
17,5	16,6	19,3	19,5	17,2	17,8	16,3	18	16,6	17,4	17,8	18,1
18,1	19,1	20,4	16,6	17,9	16,3	14,4	16,1	15,9	18,2	13,9	17

20,4	19,7	23,6	17,9	19,6	22,4	18,2	16,5	18,4	20,3	21,6	20,6
20,8	23	19,3	19,9	18,7	18,3	11,8	19,4	12,5	12,2	21,2	14,4
19,5	17,2	15,2	16,7	17,9	17,7	16,4	16,6	16,1	15,8	19,7	16,6
20	19,9	21,1	21,1	19,3	17,9	16,8	17	19,6	17,8	20	18,7
23,4	23,5	18,1	20,5	20,7	20,7	22,9	20,2	18,6	20,6	22,3	21,3
10,3	11,3	15	14,2	11,2	12,9	13,2	14	12,3	14,9	16,8	17,7
20	29,6	26,6	28	30,2	29,6	22,7	28,3	30	26,6	28,9	26
21,2	20,5	21,8	19	24,4	20,9	18,7	20,9	20,1	22,1	22,6	11,6
22,4	22,5	24,4	23,9	24,6	20,3	22	21,4	22,8	23,7	23,7	11,8
19,7	20,2	20,1	20,2	21,2	22,1	20,1	18	20,8	18,7	18,6	13,4
22,1	23,3	20,6	23,4	23,6	23,2	21,4	27	24,8	27	24,9	11,4
21,2	19,6	20,1	20,8	21,5	20,5	21,8	20,7	22,1	20,7	20,7	11,5

**LH**						**RH**					
8,4	9,9	9,5	10,5	9,5	12,3	8,6	9,6	9,7	11,6	9,7	9,6
9,6	12	10,6	11,4	11,4	10,5	11,1	11,2	11,4	10,7	12,3	12,6
10,9	14,7	11,1	11,9	13,3	11	11,4	12,8	10,8	12,8	12,7	11,7
12	12,1	11,3	15,4	12,6	11,3	10,9	14,6	11,5	11,4	13	10,4
11,2	14,8	10,7	10,6	11,6	10,8	10,8	10,8	10,5	12,4	10,6	9,5
12	11	11,4	10,3	10,9	11,6	10,9	10,8	12,9	11,9	11,5	11,1
116,2	130,3	115,3	126	105,6	96,6	124,4	105,6	127,7	118,1	102,5	105,3
9,8	10,5	10,1	10,5	11	10,3	9,8	10,2	10,7	9	10,8	10,1
17,7	20,8	18,3	17,3	15	16,4	18,7	17,3	15,7	19,3	16,1	19,2
13,1	11,8	12,6	11,5	13,4	13,5	11,2	10,9	12,3	11,7	13,2	13,7
13,6	19,6	11,7	13	19,5	10	12,2	18,6	15,1	12,1	20,8	12,6
8,1	9	7,8	8,2	8,6	8,4	9,1	9,7	8,1	8,9	8,2	8,7
10	10,6	6,2	11,3	6,3	10,7	9,6	11,7	6,9	12,1	8,6	12,7
10,6	10	9,2	10,8	11,8	9,3	9,5	9,4	9,4	11,1	11,6	10,1
8,5	8,8	10,2	9,9	11,4	9,6	15,2	13,2	14,4	14,7	14,7	16,7
15,3	14,8	15,4	13	12,9	12,8	12,9	14,5	17,9	15,6	16,7	15,6
12,1	11,7	12,4	11,2	11,1	11,6	13,6	11,3	12,8	12,9	10,3	11,9

15,8	14,8	14,6	11,1	10,6	11,8	13,3	13,7	12,3	13,4	13,2	16,1
13,1	15	15,6	13,1	13,5	13,4	11,1	10,5	12,6	12,5	11,4	10,3
11,2	8,8	9,6	11,8	12,5	11,4	11	14,1	12,2	8,6	10,9	9
12,1	10,9	11,4	10,5	10,9	11,5	9,8	9,3	10	9,9	10,1	10,1

Vertical impulse (N*sec)

**LF**						**RF**					
52,94	54,609	60,472	66,477	61,439	68,238	63,41	68,459	84,304	67,022	64,264	70,189
48,842	56,602	54,98	50,695	50,257	53,799	65,021	78,188	72,839	75,546	77,766	77,853
86,747	100,352	96,885	83,511	80,789	102,743	84,135	87,422	97,32	94,002	89,403	91,061
74,459	69,272	74,155	75,873	72,275	82,863	75,747	89,887	79,666	71,206	79,061	69,274
53,436	60,349	55,709	52,721	53,423	60,652	63,154	67,242	64,241	68,866	65,133	66,103
78,281	75,033	87,07	77,409	73,571	73,708	69,371	77,344	75,781	77,391	74,853	79,834
62,59	70,298	71	62,669	60,054	58,507	49,563	45,24	50,88	44,756	44,579	48,931
56,325	53,435	62,185	62,605	55,42	57,313	52,43	57,748	53,395	55,919	57,206	58,327
71,008	74,767	79,633	64,864	69,876	63,632	56,2	62,875	62,042	71,044	54,528	66,443
60,005	58	69,535	52,769	57,647	66,039	53,52	48,664	54,199	59,592	63,65	60,729
81,684	90,405	75,735	78,152	73,69	72,088	46,497	76,393	49,218	47,895	83,272	56,437
51,515	45,582	40,115	44,248	47,331	46,738	43,398	43,845	42,688	41,834	52,205	43,824
53,462	53,037	56,242	56,179	51,455	47,839	44,777	45,306	52,357	47,545	53,449	49,982
77,929	78,491	60,338	68,393	69,172	68,98	76,303	67,418	61,885	68,639	74,225	70,883
34,5	37,519	49,878	47,253	37,202	42,886	44,097	46,658	40,862	49,578	55,924	59,112
95,218	140,987	126,613	133,064	143,42	140,825	107,809	134,631	142,631	126,704	137,242	123,644
74,814	72,343	76,963	67,062	86,067	73,945	65,92	73,711	70,999	78,047	79,829	74,475
76,54	76,654	83,107	81,593	83,98	69,136	75,199	72,968	77,727	80,873	80,787	82,584
85,151	87,307	86,556	87,37	91,643	95,411	86,742	77,761	89,789	80,85	80,299	82,917
86,549	91,542	80,737	91,702	92,397	90,874	84,024	105,817	97,259	105,893	97,599	97,211
81,255	74,927	76,769	79,623	82,053	78,297	83,537	79,241	84,626	79,351	79,142	72,725

**LH**						**RH**					
25,848	30,69	29,373	32,425	29,193	38,005	26,443	29,79	29,91	35,88	30,108	29,73

33,078	41,285	36,248	39,227	39,111	36,126	38,141	38,474	39,018	36,754	42,048	43,3
45,921	61,983	46,98	50,095	56,06	46,221	48,099	53,937	45,607	54,059	53,434	49,33
42,539	42,837	39,735	54,219	44,534	39,791	38,434	51,452	40,686	40,199	45,994	36,729
34,084	44,964	32,62	32,352	35,327	32,878	32,917	32,847	31,909	37,596	32,221	29,01
55,125	50,682	52,433	47,606	50,2	53,244	50,342	49,974	59,551	54,692	52,961	51,148
34,87	39,091	34,576	37,795	31,67	28,985	37,32	31,675	38,308	35,417	30,737	31,592
31,681	33,691	32,561	33,637	35,43	33,233	31,417	32,89	34,541	29,027	34,776	32,599
69,3	81,333	71,432	67,532	58,512	64,224	73,049	67,542	61,415	75,696	62,885	75,138
38,54	34,696	37,019	33,914	39,51	39,857	32,954	31,95	36,31	34,34	38,941	40,242
53,317	77,099	45,858	51,046	76,535	39,421	48,081	73,244	59,251	47,541	81,743	49,44
21,32	23,759	20,596	21,833	22,819	22,312	24,114	25,615	21,484	23,541	21,826	23,04
26,59	28,383	16,454	30,183	16,92	28,609	25,517	31,335	18,468	32,354	22,935	33,746
35,257	33,446	30,706	35,847	39,238	31,154	31,755	31,215	31,263	37,134	38,548	33,609
28,46	29,289	33,989	32,928	38,017	32,022	50,691	43,973	47,924	48,968	48,959	55,807
72,731	70,591	73,483	61,657	61,351	61,058	61,484	68,787	85,306	74,235	79,432	74,063
42,8	41,455	43,944	39,577	39,043	41,043	47,897	40,054	45,203	45,419	36,529	42,174
53,954	50,461	49,741	37,74	36,147	40,335	45,497	46,92	42,047	45,886	44,969	55,016
56,471	64,834	67,381	56,378	58,104	57,65	48,088	45,159	54,493	53,747	48,98	44,526
44,125	34,434	37,517	46,283	49,049	44,624	43,241	55,425	47,97	33,58	42,819	35,158
46,38	41,766	43,719	40,128	41,786	44,053	37,426	35,64	38,219	37,907	38,571	38,513

Maximum peak pressure (kPa)

**LF**						**RF**					
60,1	54,8	60,8	64,4	57	61,3	62,4	65,4	79,2	61,2	57,1	67,2
46,8	46,6	48,9	42,3	41,9	43,4	52,1	58,8	54,8	58,2	57,4	56,7
49,1	52,5	57,5	46,6	44	55,8	48,5	51,6	55,5	51	52,7	49,4
58,1	52,7	53,3	56,4	55,6	55,4	56,6	70,8	60,8	55	61,4	53,3
51,2	58,9	60,4	54,4	56,9	65,3	56	55,8	59,8	59,5	59,3	59,8
49,4	45,9	51,5	44,8	44,8	43,6	44,7	48,6	46,2	46	45,1	48
570,9	581	585,9	521,3	527,8	561,9	496,4	418,5	462,1	420	443,3	502,2
55,3	44,4	51,4	59,5	48,5	48,2	52	48,1	45,8	51,3	49,6	56,3
71,4	70	68,8	62,7	67,2	65,2	57,2	61	57,5	69,7	59,7	67,2

54,1	54,1	59,9	47,4	48,1	55	48,1	45	47,6	55,7	57,3	51,6
51,3	54,6	45,8	46,1	44,6	43,4	27,2	51,2	29,4	26,9	52,7	34,5
62,4	53,8	56,3	49,2	51,9	53,2	52	51,3	59,6	47,4	59,9	53,1
65,8	58,9	61	57,7	55,5	51,1	56,4	50,4	59,2	49,4	58,4	55,4
58,9	63,4	59,7	52,2	54,7	58	58,6	53,4	58,6	51,4	54,7	56,7
36,4	38,7	42,9	36,2	30,9	32,7	46,4	50,4	36,9	43,6	45,3	42,8
56,3	60	56,7	60	59,4	60,9	51,6	57,4	62,5	56,4	59,7	56,8
48,6	50,3	47	45	56,3	44,5	42,8	50,2	47,8	51,5	52,6	47,3
56	51,7	57,8	54,5	63,5	47,3	51,7	51,3	50,4	56,3	54,7	53,2
61,6	64,7	60,3	54,3	62,9	64,1	59	51,7	57,4	55,6	56,5	57,6
55,8	64,2	53,4	58,3	61,7	61,7	54,7	63,3	59,5	62,9	53,2	60,3
53,7	52,4	49,5	52,1	50,2	50,3	55,6	53,3	53,7	51,2	50,7	46,5

**LH**						**RH**					
34,7	37,1	34,6	35,9	32	43,3	32,6	36	34,1	38,2	31,2	34
28,7	31,5	30,5	32,2	31,6	28,9	34,2	30,1	33,9	31,2	33,3	33,7
26,2	34,7	27,2	27,9	30,5	26,1	30,8	29,2	30,7	30,8	30,7	27,7
28,9	29,8	25,5	38,4	32,9	27,2	28,1	34,4	26,3	28,9	33,6	24,4
32,2	37,1	34,5	32,1	41,3	31,9	30,5	26,5	33,3	33,6	33,6	28,9
32,6	29,6	29,6	25,5	28,7	29,9	29,3	27,7	33,4	30,1	29,6	27,9
300,9	314,8	286,3	302,7	273,8	265	348,4	266,7	331,3	296,1	302,1	310,3
30,9	27,3	27,2	31,1	32,1	30,5	30,1	27,9	30,6	27,7	31,2	30,4
40,2	39,6	34,7	37	31,8	36,9	39,8	37,4	34,3	37,8	38,2	36,3
36,1	36,2	34,7	34,9	36,6	34,9	32,6	35,1	35,8	32,9	37,7	38,3
32	53,4	28,4	28,9	50,4	23,6	28,5	47,8	35,8	27,7	49,6	28,4
32,6	34,3	35,3	30,5	33,3	34,1	37,6	36,9	38,6	31,5	30,3	33,6
43,6	39,5	26,7	41	31,4	43,1	39	42,1	39,9	38,7	36	44,4
28,5	26,9	27,8	27,1	29	25	24,9	26,2	32,4	27,9	29,7	31,1
26	26	27,1	25,6	27,7	23,9	48,2	40,2	36,9	37,4	38,1	40
36,9	28,3	30	25,2	25	26,4	35,5	27,3	33,1	28,7	29,3	30,2
29,8	27,6	29,8	26,6	26,8	25	33,5	26	30,7	30,6	23,6	26,3
39,8	38,2	32,8	29,2	30,8	30,6	38,3	34,4	30,6	36,6	38,8	40,9

31,4	33,8	35,3	31,3	32,6	30,8	28,2	27,4	31,2	32,8	30,2	28,5
25,6	23	23,2	28,8	25,6	26,6	27,3	31,2	26,8	22,3	25,2	23
33,6	30,5	28,3	28,8	29,5	31,7	26,7	26,3	24,7	28,1	24,5	26,4
