# Supplementary material for: Kinetic gait analysis in healthy dogs and dogs with osteoarthritis: An evaluation of precision and overlap performance of a pressure-sensitive walkway and the use of symmetry indices
Source: PLoS One. 2020 Dec 15;15(12):e0243819. doi: 10.1371/journal.pone.0243819 (PMC7737891; doi:10.1371/journal.pone.0243819)
Supplement: S6 File — Coefficients of variation calculated for repeated measurements of vertical impulse and maximum peak pressure in 21 dogs with osteoarthritis. In Table 3 precision is compared across limbs affected by osteoarthritis compared with contralateral, ipsilateral and diagonal limbs. (PDF) [file pone.0243819.s006.pdf]

S6 File. Precision of vertical impulse and maximum peak pressure measured in individual limbs of dogs previously diagnosed with osteoarthritis

Coefficients of variation calculated for repeated measurements of vertical impulse and maximum peak pressure in 21 dogs with osteoa
Precision is compared across limbs affected by osteoarthritis compared with contralateral, ipsilateral and diagonal limbs.

Vertical Impulse

**DOG 1-21**	1	2	3	4	5	6	7	8	9	10
**AFFECTED LIMB**	13.6	13.3	11.1	11.0	9.8	17.1	14.2	20.6	21.1	17.6
	11.3	13.7	10.5	14.1	9.3	17.7	16.5	23.8	19.6	19.9
	12.8	12.3	12.6	12.2	10.0	19.6	16.0	23.0	21.0	18.3
	12.9	13.4	12.5	8.6	9.9	21.5	14.8	19.8	21.5	17.3
	10.3	13.2	11.4	10.9	10.1	19.9	14.6	19.2	20.5	17.6
	11.9	16.1	10.3	9.0	10.1	22.1	15.7	24.4	23.5	20.0
CV	9.95%	9.38%	8.56%	18.60%	3.05%	10.13%	5.88%	10.13%	6.14%	6.55%

	12	13	14	15	16	17	18	19	20	21
	10.6	8.5	15.3	15.1	165.2	16.3	14.4	18.2	11.8	16.4
	10.0	8.8	14.8	16.8	150.8	18.0	16.1	16.5	19.4	16.6
	9.2	10.2	15.4	16.4	169.6	16.6	15.9	18.4	12.5	16.1
	10.8	9.9	13.0	16.8	149.2	17.4	18.2	20.3	12.2	15.8
	11.8	11.4	12.9	16.2	148.6	17.8	13.9	21.6	21.2	19.7
	9.3	9.6	12.8	17.3	163.1	18.1	17.0	20.6	14.4	16.6
CV	9.62%	10.71%	8.98%	4.60%	5.88%	4.35%	10.05%	9.80%	26.57%	8.43%

Dog 1-21	1	2	3	4	5	6	7	8	9	10
**IPSILATERAL LIMB**	18.7	22.0	20.1	21.4	21.8	8.4	9.6	10.9	12.0	11.2
	20.9	21.4	18.0	27.0	20.7	9.9	12.0	14.7	12.1	14.8
	20.1	22.8	20.8	24.8	22.1	9.5	10.6	11.1	11.3	10.7

	22.1	23.7	18.7	27.0	20.7	10.5	11.4	11.9	15.4	10.6
	22.6	23.7	18.6	24.9	20.7	9.5	11.4	13.3	12.6	11.6
	21.1	24.2	19.2	24.8	19.0	12.3	10.5	11.0	11.3	10.8
CV	6.71%	4.78%	5.41%	8.21%	5.24%	13.10%	7.82%	12.68%	12.28%	13.80%
	12	13	14	15	16	17	18	19	20	21
	23.4	10.3	20.0	10.9	124.4	9.8	18.7	11.2	12.2	9.1
	23.5	11.3	29.6	10.8	105.6	10.2	17.3	10.9	18.6	9.7
	18.1	15.0	26.6	12.9	127.7	10.7	15.7	12.3	15.1	8.1
	20.5	14.2	28.0	11.9	118.1	9.0	19.3	11.7	12.1	8.9
	20.7	11.2	30.2	11.5	102.5	10.8	16.1	13.2	20.8	8.2
	20.7	12.9	29.6	11.1	105.3	10.1	19.2	13.7	12.6	8.7
CV	9.62%	14.89%	14.00%	6.87%	9.54%	6.51%	8.93%	9.15%	24.26%	6.77%
Dog 1-21	1	2	3	4	5	6	7	8	9	10
**CONTRALATERAL LIMB**	12.1	15.8	13.1	11.2	12.1	20.5	18.9	20.0	21.5	20.8
	11.7	14.8	15.0	8.8	10.9	22.2	22.8	20.7	25.5	22.1
	12.4	14.6	15.6	9.6	11.4	27.3	21.2	23.1	22.6	21.1
	11.2	11.1	13.1	11.8	10.5	21.7	22.0	22.3	20.2	22.7
	11.1	10.6	13.5	12.5	10.9	20.8	22.7	21.2	22.4	21.4
	11.6	11.8	13.4	11.4	11.5	22.7	22.7	21.6	19.6	21.7
CV	4.31%	16.83%	7.70%	12.87%	5.06%	11.00%	6.95%	5.18%	9.55%	3.20%
Dog 12-21	12	13	14	15	16	17	18	19	20	21
	9.5	15.2	12.9	17.0	208.6	17.5	18.1	20.4	20.8	19.5
	9.4	13.2	14.5	16.3	234.3	16.6	19.1	19.7	23.0	17.2
	9.4	14.4	17.9	18.9	236.7	19.3	20.4	23.6	19.3	15.2
	11.1	14.7	15.6	16.8	208.9	19.5	16.6	17.9	19.9	16.7
	11.6	14.7	16.7	16.0	200.2	17.2	17.9	19.6	18.7	17.9

	10.1	16.7	15.6	16.0	195.0	17.8	16.3	22.4	18.3	17.7
CV	9.37%	7.70%	11.14%	6.49%	8.19%	6.50%	8.51%	10.04%	8.58%	8.19%

Dog 1-11	1	2	3	4	5	6	7	8	9	10
**DIAGONAL LIMB**	21.2	22.4	19.7	22.1	21.2	8.6	11.1	11.4	10.9	10.8
	20.5	22.5	20.2	23.3	19.6	9.6	11.2	12.8	14.6	10.8
	21.8	24.4	20.1	20.6	20.1	9.7	11.4	10.8	11.5	10.5
	19.0	23.9	20.2	23.4	20.8	11.6	10.7	12.8	11.4	12.4
	24.4	24.6	21.2	23.6	21.5	9.7	12.3	12.7	13.0	10.6
	20.9	20.3	22.1	23.2	20.5	9.6	12.6	11.7	10.4	9.5
CV	8.39%	7.07%	4.34%	5.09%	3.41%	9.98%	6.40%	7.10%	13.02%	8.69%

Dog 12-21	12	13	14	15	16	17	18	19	20	21
	22.9	13.2	22.7	12.0	116.2	9.8	17.7	13.1	13.6	8.1
	20.2	14.0	28.3	11.0	130.3	10.5	20.8	11.8	19.6	9.0
	18.6	12.3	30.0	11.4	115.3	10.1	18.3	12.6	11.7	7.8
	20.6	14.9	26.6	10.3	126.0	10.5	17.3	11.5	13.0	8.2
	22.3	16.8	28.9	10.9	105.6	11.0	15.0	13.4	19.5	8.6
	21.3	17.7	26.0	11.6	96.6	10.3	16.4	13.5	10.0	8.4
CV	7.37%	14.11%	9.62%	5.33%	10.89%	3.94%	11.09%	6.65%	27.82%	5.01%

Maximum Peak Pressure

**Dog 1-11**	1	2	3	4	5	6	7	8	9	10
**AFFECTED LIMB**	99.4	75.4	124.5	148.3	90.9	114.6	104.2	128.9	64.7	68.7
	113.6	66.9	118.9	151.1	87.7	159.0	97.4	92.4	78.5	73.4
	116.8	77.1	111.5	152.0	93.3	128.4	95.9	78.8	59.8	67.0
	126.7	67.9	119.3	168.0	104.7	103.1	96.5	90.8	69.0	69.2
	107.3	64.6	102.8	151.7	98.0	206.9	107.0	81.8	69.0	76.9
	114.3	73.8	108.5	163.8	94.3	144.8	100.2	100.7	61.4	72.1

**CV**	8.13%	7.24%	7.04%	5.15%	6.26%	26.12%	4.51%	18.96%	10.08%	5.10%

Dog 12-21	12	13	14	15	16	17	18	19	20	21
	126.1	119.3	227.4	107.9	114.5	104.6	129.8	107.5	82.9	84.3
	117.5	119.1	192.1	99.2	129.1	80.4	117.5	103.2	117.5	78.9
	152.0	120.0	170.4	101.4	122.1	83.8	101.0	103.0	98.1	72.6
	163.1	118.7	188.2	116.1	109.5	95.4	120.8	110.4	100.4	83.9
	137.7	115.4	190.2	116.4	117.7	75.4	109.5	107.0	98.3	65.5
	150.0	129.7	205.7	106.3	126.5	83.3	135.0	104.8	94.5	80.2
CV	12.18%	4.03%	9.82%	6.68%	6.19%	12.38%	10.58%	2.70%	11.33%	9.38%

IPSILATERAL LIMB

Dog 1-11	1	2	3	4	5	6	7	8	9	10
	99.9	95.2	91.5	136.9	72.9	112.9	67.2	149.5	118.7	94.6
	89.4	86.1	75.6	101.6	66.8	174.7	72.1	163.6	118.1	97.4
	107.9	91.1	87.5	98.8	75.3	142.3	62.1	153.7	120.6	108.1
	101.8	82.1	73.7	105.2	82.7	111.7	69.1	143.1	112.7	96.7
	111.2	87.4	89.2	112.2	76.2	199.4	60.0	142.0	118.0	80.1
	96.2	79.2	83.8	113.6	78.2	109.1	66.5	138.0	118.1	85.0
**CV**	7.80%	6.71%	8.81%	12.37%	7.07%	26.80%	6.74%	6.31%	2.24%	10.59%

Dog 12-21	12	13	14	15	16	17	18	19	20	21
	82.5	97.9	99.0	89.0	89.3	90.4	139.8	137.5	119.7	125.3
	99.7	116.1	163.2	88.5	97.5	112.7	129.2	130.9	131.2	112.0
	92.4	108.8	102.0	84.9	90.1	118.8	133.6	131.9	143.9	117.3
	100.2	110.2	105.6	122.4	82.6	130.1	144.8	136.2	153.6	114.6
	88.6	105.5	109.4	87.1	92.8	125.3	155.4	136.8	134.9	111.6
	123.1	82.4	93.3	79.1	87.7	105.3	148.9	142.5	135.7	107.2

CV	14.45%	11.53%	22.88%	16.77%	5.55%	12.70%	6.86%	3.09%	8.42%	5.41%
Dog 1-11	1	2	3	4	5	6	7	8	9	10
**CONTRALATERAL LIMB**	119.2	95.2	125.8	163.1	122.6	158.5	118.8	91.8	60.6	128.2
	118.2	90.2	103.8	167.0	119.0	154.7	101.2	98.0	55.4	106.5
	140.9	88.9	129.6	171.0	115.8	149.1	92.9	82.1	69.9	137.3
	117.7	87.4	114.9	166.9	139.4	151.3	94.5	96.3	69.9	106.0
	112.0	85.3	123.1	159.1	124.1	138.8	107.6	77.4	68.9	103.0
	126.6	81.1	117.2	165.2	119.8	146.8	103.6	95.8	68.4	95.3
**CV**	8.31%	5.40%	7.76%	2.44%	6.75%	4.55%	9.19%	9.42%	9.28%	14.45%
Dog 12-21	12	13	14	15	16	17	18	19	20	21
	129.0	143.0	133.5	109.7	119.7	93.8	142.8	118.5	108.4	85.2
	136.8	150.3	135.1	123.3	121.2	77.1	118.0	112.9	91.9	76.8
	169.7	167.2	140.0	95.8	127.6	94.8	122.3	137.5	90.4	82.5
	135.4	150.7	121.6	98.0	137.9	80.6	105.8	120.0	118.7	81.3
	116.5	158.0	142.5	105.3	140.5	71.5	94.2	115.8	98.4	91.0
	140.4	142.0	132.1	96.4	136.9	74.5	104.4	114.3	105.3	94.8
CV	12.81%	6.26%	5.45%	10.14%	6.91%	12.13%	14.93%	7.55%	10.54%	7.76%
Dog 1-11	1	2	3	4	5	6	7	8	9	10
**DIAGONAL LIMB**	100.0	106.4	83.9	121.8	72.7	116.6	58.0	124.8	150.2	114.7
	85.6	92.3	79.4	137.2	68.8	176.8	65.5	133.1	134.5	121.5
	95.4	108.0	79.9	104.7	72.4	113.4	64.2	139.2	143.5	94.8
	81.6	81.7	86.8	117.9	67.4	110.5	64.5	141.5	135.8	98.8
	97.3	86.8	79.0	96.8	74.3	166.3	67.8	158.3	135.7	107.8
	97.6	84.1	82.6	113.2	73.3	102.7	63.8	132.2	135.5	104.3
CV	8.04%	12.23%	3.75%	12.19%	3.83%	24.33%	5.09%	8.30%	4.53%	9.29%
Dog 12-21	12	13	14	15	16	17	18	19	20	21

	79.2	110.3	105.2	81.3	93.6	112.4	157.3	138.5	116.4	112.5
	85.1	90.1	99.0	94.9	88.4	120.0	149.2	140.7	125.9	114.4
	80.5	96.0	91.5	74.8	89.2	99.9	161.8	140.4	110.3	107.7
	97.2	83.8	105.9	76.7	95.8	109.5	170.3	132.8	124.6	105.7
	87.1	107.7	110.9	92.6	87.4	139.7	159.2	159.4	123.5	100.7
	85.5	106.6	103.2	76.4	80.3	106.0	123.9	163.0	123.8	109.1
CV	7.45%	10.87%	6.51%	10.63%	6.06%	12.22%	10.47%	8.45%	5.06%	4.53%

ırthritis.

11

10.0
10.6
6.2
11.3
6.3
10.7

25.15%

11

20.0
19.9
21.1

21.1
19.3
17.9

6.06%

11
9.6
11.7
6.9
12.1
8.6
12.7

22.17%

11

16.8

17.0

19.6

17.8

20.0

18.7

7.30%

11

138.4

123.0

123.4

105.7

105.2

106.9

11.48%

11
136.8
157.1
159.0
163.2
164.9
172.5

7.61%

11

137.2
117.8
136.5
124.2
126.9
133.4

5.94%

11

157.4
147.4
159.2
154.9
149.3
160.0

3.40%
