# Supplementary material for: Kinetic gait analysis in healthy dogs and dogs with osteoarthritis: An evaluation of precision and overlap performance of a pressure-sensitive walkway and the use of symmetry indices
Source: PLoS One. 2020 Dec 15;15(12):e0243819. doi: 10.1371/journal.pone.0243819 (PMC7737891; doi:10.1371/journal.pone.0243819)
Supplement: S7 File — Inter- and intraanalytical coefficients of variation calculated for individual limbs measured in 10 clinically healthy dogs. (PDF) [file pone.0243819.s007.pdf]

S7 File. Precision of temporal characteristics and vertical gro
in clinically healthy dogs.

Inter- and intraanalytical coefficients of variation calculated for individual limbs measured in 10 cl

Abbreviations:

RF	Measured ground reaction forces in 6 recordings of
RH	Measured ground reaction forces in 6 recordings of
LF	Measured ground reaction forces in 6 recordings of
LH	Measured ground reaction forces in 6 recordings of
CV	Coefficient of variation

Stance time

	Dog	LF	RF	LH	RH
**Inter-analytical CV**	1	0.30%	1.27%	0.67%	1.35%
	2	4.60%	4.07%	4.44%	2.73%
	3	1.07%	1.53%	3.25%	2.76%
	4	1.03%	0.51%	1.68%	0.81%
	5	3.29%	4.24%	2.53%	4.38%
	6	2.79%	2.55%	3.87%	4.80%
	7	0.50%	1.50%	2.96%	1.07%
	8	2.71%	1.51%	4.02%	3.68%
	9	0.44%	2.24%	0.46%	0.23%
	10	0.63%	0.00%	3.13%	5.60%

	Dog	LF	RF	LH	RH
**Intra- analytical CV**	1	3.80%	2.82%	4.62%	4.34%
	2	5.49%	4.84%	6.59%	6.63%
	3	1.89%	4.43%	4.55%	3.07%
	4	4.11%	4.25%	6.56%	4.55%
	5	5.32%	4.84%	2.26%	3.08%
	6	2.18%	2.69%	2.67%	2.28%
	7	5.28%	4.66%	5.23%	7.35%
	8	3.70%	3.84%	5.07%	6.16%
	9	2.77%	2.91%	4.53%	2.90%
	10	5.05%	5.20%	8.59%	6.19%

Peak vertical force % BW

**Inter-analytical CV**	1	3.00%	1.32%	4.08%	1.42%
	2	6.72%	5.74%	7.17%	10.97%
	3	5.60%	5.28%	5.74%	10.26%
	4	2.22%	0.25%	0.97%	0.63%
	5	4.13%	0.97%	5.40%	1.46%
	6	5.73%	2.39%	11.00%	6.95%

	7	1.48%	1.18%	9.10%	1.54%
	8	1.85%	0.39%	7.73%	3.39%
	9	3.56%	3.25%	1.29%	1.80%
	10	4.15%	1.19%	2.20%	2.07%
**Intra-analytical CV**	1	7.43%	5.02%	15.08%	7.92%
	2	3.49%	4.06%	7.03%	4.97%
	3	6.77%	5.78%	13.84%	9.36%
	4	2.70%	3.93%	5.04%	6.14%
	5	3.71%	5.99%	7.60%	2.58%
	6	4.83%	2.29%	5.38%	6.54%
	7	4.09%	3.96%	6.32%	11.87%
	8	3.74%	3.70%	3.29%	2.14%
	9	3.29%	5.97%	3.73%	5.10%
	10	6.05%	3.80%	9.40%	10.02%

Vertical impulse N*sec

**Inter-analytical CV**	1	5.32%	3.02%	7.13%	1.18%
	2	0.46%	0.06%	1.13%	3.15%
	3	1.50%	2.96%	3.62%	0.39%
	4	2.83%	1.09%	4.61%	2.01%
	5	5.46%	2.35%	1.40%	5.95%
	6	7.94%	5.09%	14.32%	10.75%
	7	1.17%	4.09%	9.73%	3.06%
	8	3.68%	3.79%	10.30%	7.99%
	9	2.03%	2.10%	2.20%	2.02%
	10	4.72%	2.17%	10.35%	8.19%
**Intra-analytical CV**	1	7.94%	4.92%	12.22%	3.52%
	2	7.09%	6.77%	3.39%	11.16%
	3	8.37%	6.68%	5.76%	2.86%
	4	5.78%	7.10%	6.25%	6.16%
	5	8.39%	8.32%	4.11%	6.52%
	6	3.70%	3.75%	5.33%	3.33%
	7	9.02%	7.84%	6.71%	3.94%
	8	6.90%	2.73%	3.77%	6.38%
	9	1.89%	6.93%	2.98%	3.86%
	10	8.53%	6.07%	8.97%	6.07%

Peak vertical force N

**Inter-analytical CV**	1	2.98%	1.33%	4.11%	1.35%
	2	6.73%	5.75%	7.16%	10.94%
	3	5.59%	5.28%	5.68%	10.33%

	4	2.26%	0.24%	0.96%	0.63%
	5	4.14%	0.99%	5.36%	1.46%
	6	5.74%	2.37%	11.03%	7.02%
	7	1.37%	1.14%	8.60%	1.38%
	8	1.94%	0.50%	7.88%	3.53%
	9	3.35%	3.32%	1.55%	1.83%
	10	4.13%	1.20%	2.22%	2.02%
**Intra-analytical CV**	1	7.42%	5.01%	15.05%	7.95%
	2	3.50%	4.07%	6.99%	4.93%
	3	6.76%	5.77%	13.84%	9.43%
	4	2.72%	3.97%	5.00%	6.08%
	5	3.72%	5.97%	7.61%	2.59%
	6	4.81%	2.32%	5.39%	6.61%
	7	4.09%	3.96%	6.41%	11.93%
	8	3.75%	3.72%	3.28%	2.09%
	9	3.28%	5.98%	3.75%	5.12%
	10	6.10%	3.81%	9.45%	10.05%

Stride length

**Inter-analytical CV**	1	3.21%	1.91%	1.88%	2.51%
	2	2.51%	0.84%	2.00%	1.18%
	3	1.90%	1.95%	0.15%	0.95%
	4	1.16%	1.54%	1.84%	1.80%
	5	0.40%	0.00%	0.01%	0.46%
	6	0.26%	0.41%	0.69%	0.42%
	7	0.64%	0.16%	2.57%	0.32%
	8	1.03%	0.14%	2.39%	2.94%
	9	1.86%	1.55%	2.04%	1.98%
	10	3.42%	1.97%	2.60%	4.08%
**Intra-analytical CV**	1	3.58%	3.05%	3.65%	3.85%
	2	1.91%	2.41%	2.17%	2.70%
	3	3.03%	2.09%	4.26%	1.94%
	4	2.80%	1.73%	1.87%	2.29%
	5	0.88%	1.72%	1.91%	1.13%
	6	2.51%	2.99%	1.75%	1.90%
	7	4.23%	3.13%	5.79%	4.72%
	8	2.43%	1.75%	2.54%	2.01%
	9	2.24%	2.47%	3.31%	3.07%
	10	2.63%	2.86%	3.65%	3.28%

Stride velocity

**Inter-analytical CV**	1	2.97%	1.66%	1.53%	1.37%
	2	6.99%	5.78%	6.04%	5.42%
	3	0.89%	0.86%	2.19%	1.15%
	4	2.38%	2.45%	2.97%	1.82%
	5	2.98%	2.98%	1.12%	4.41%
	6	1.30%	2.70%	2.99%	1.43%
	7	0.50%	1.88%	0.30%	1.56%
	8	1.64%	1.65%	0.05%	1.16%
	9	0.71%	0.59%	1.26%	0.68%
	10	3.21%	1.10%	5.22%	3.54%
**Intra-analytical CV**	1	4.54%	4.28%	5.84%	5.68%
	2	3.58%	2.85%	5.10%	7.11%
	3	5.28%	2.89%	6.72%	2.88%
	4	5.99%	5.41%	5.81%	5.29%
	5	5.31%	4.96%	4.32%	3.87%
	6	4.38%	4.99%	3.39%	3.79%
	7	4.58%	3.86%	5.85%	5.64%
	8	7.57%	4.95%	5.38%	7.22%
	9	4.65%	3.47%	6.62%	5.70%
	10	6.05%	9.62%	5.71%	7.74%
**Vertical impulse % BW sec**					
**Inter-analytical CV**	1	5.27%	3.21%	6.97%	1.04%
	2	0.51%	0.09%	1.04%	3.06%
	3	1.54%	2.91%	3.56%	0.40%
	4	2.84%	1.03%	4.60%	2.10%
	5	5.49%	2.42%	1.15%	6.15%
	6	7.91%	5.09%	14.30%	10.64%
	7	0.54%	4.14%	8.50%	2.88%
	8	3.34%	3.72%	10.31%	7.78%
	9	2.10%	2.03%	1.16%	1.34%
	10	4.74%	2.12%	10.22%	8.15%
**Intra-analytical CV**	1	8.00%	4.92%	12.18%	3.43%
	2	7.30%	6.73%	3.26%	11.09%
	3	8.41%	6.65%	5.89%	2.89%
	4	5.75%	7.24%	6.49%	5.94%
	5	8.30%	8.34%	4.00%	6.51%
	6	3.70%	3.79%	5.28%	3.12%
	7	9.05%	7.78%	6.51%	3.97%
	8	6.92%	2.83%	3.64%	6.44%
	9	1.93%	6.95%	3.17%	3.80%
	10	8.56%	6.07%	9.17%	6.20%

Maximum peak pressure

**Inter-analytical CV**	1	2.03%	5.94%	2.55%	0.02%
	2	1.09%	1.57%	0.24%	2.74%
	3	3.43%	4.92%	4.78%	5.68%
	4	0.80%	4.80%	1.70%	0.79%
	5	4.17%	2.02%	0.25%	1.01%
	6	2.37%	1.58%	4.34%	5.44%
	7	1.46%	3.24%	7.45%	1.03%
	8	0.74%	0.48%	4.50%	4.52%
	9	1.15%	2.76%	0.83%	0.07%
	10	1.14%	0.53%	3.31%	7.73%
**Intra-analytical CV**	1	12.70%	8.53%	8.28%	13.19%
	2	10.18%	6.38%	5.51%	12.95%
	3	9.19%	3.98%	11.66%	3.22%
	4	5.36%	6.45%	4.91%	8.67%
	5	5.87%	5.57%	6.18%	4.88%
	6	5.11%	5.55%	7.79%	5.61%
	7	4.41%	3.87%	5.76%	9.10%
	8	6.57%	4.02%	6.82%	3.09%
	9	5.56%	7.32%	5.42%	6.56%
	10	7.83%	5.88%	9.80%	3.93%

Stride time

**Inter-analytcal CV**	1	0.37%	0.56%	0.56%	1.31%
	2	4.40%	4.90%	3.89%	4.02%
	3	1.06%	2.77%	2.09%	2.12%
	4	1.28%	0.95%	1.12%	0.00%
	5	3.36%	2.65%	1.17%	3.97%
	6	1.43%	2.07%	2.21%	0.94%
	7	0.64%	0.16%	2.57%	0.32%
	8	0.38%	1.51%	2.63%	2.07%
	9	2.66%	2.19%	0.71%	1.29%
	10	0.13%	0.80%	2.95%	0.53%
**Intra-analytical CV**	1	1.20%	2.01%	2.34%	2.21%
	2	4.33%	3.36%	5.27%	5.21%
	3	2.96%	2.31%	3.21%	1.67%
	4	4.92%	4.15%	5.14%	4.30%
	5	4.71%	3.83%	2.93%	4.02%
	6	2.39%	2.72%	1.81%	2.04%
	7	4.23%	3.13%	5.79%	4.72%

	8	5.49%	3.67%	3.89%	5.52%
	9	2.85%	2.21%	3.30%	2.87%
	10	3.65%	3.41%	6.97%	5.75%

Swing time

**Inter-analytical CV**	1	2.02%	0.00%	2.97%	1.68%
	2	3.69%	5.69%	1.15%	3.07%
	3	3.52%	3.97%	1.19%	1.85%
	4	1.24%	2.54%	0.37%	0.00%
	5	2.33%	0.39%	1.04%	1.38%
	6	0.00%	0.00%	1.09%	1.72%
	7	0.44%	2.15%	1.96%	1.19%
	8	2.53%	0.99%	0.41%	1.28%
	9	5.11%	2.04%	1.49%	4.04%
	10	2.54%	2.89%	3.29%	4.65%

**Intra-analytical CV**	1	2.75%	3.44%	2.77%	2.95%
	2	3.91%	5.29%	6.13%	4.71%
	3	2.38%	4.45%	3.16%	2.67%
	4	4.72%	4.43%	4.18%	5.59%
	5	1.37%	5.37%	2.43%	1.21%
	6	1.45%	1.92%	4.13%	3.46%
	7	3.62%	4.20%	6.96%	4.15%
	8	6.45%	4.56%	2.88%	2.70%
	9	5.65%	4.84%	2.61%	5.14%
	10	5.07%	3.63%	4.45%	8.57%

und reaction forces

linically healthy dogs.

[illegible] right thoracic limb
[illegible] right pelvic limb
[illegible] left thoracic limb
[illegible] left pelvic limb
