# Supplementary material for: Kinetic gait analysis in healthy dogs and dogs with osteoarthritis: An evaluation of precision and overlap performance of a pressure-sensitive walkway and the use of symmetry indices
Source: PLoS One. 2020 Dec 15;15(12):e0243819. doi: 10.1371/journal.pone.0243819 (PMC7737891; doi:10.1371/journal.pone.0243819)
Supplement: S8 File — Intraanalytical coefficients of variation calculated for individual limbs in 21 dogs with osteoarthritis. (PDF) [file pone.0243819.s008.pdf]

S8 File. Precision of temporal characteristics and vertical ground reaction forces in 21 dogs with osteoarthritis

Intraanalytical coefficients of variation calculated for individual limbs in 21 dogs with osteoarthritis.

**Abbreviations:**		
	RF	Measured ground reaction forces in 6 recordings of right thoracic limb
	RH	Measured ground reaction forces in 6 recordings of right pelvic limb
	LF	Measured ground reaction forces in 6 recordings of left thoracic limb
	LH	Measured ground reaction forces in 6 recordings of left pelvic limb
	CV	Coefficient of variation

Stance time (sec)

**Dog 1-12**		**1**	**2**	**3**	**4**	**5**	**6**	**7**	**8**	**9**	**10**	**11**	**12**
	LF	0,41	0,44	0,57	0,52	0,5	0,47	0,49	0,43	0,43	0,5	0,55	0,45
	LF	0,47	0,5	0,6	0,54	0,52	0,49	0,53	0,51	0,44	0,48	0,56	0,45
	LF	0,46	0,47	0,54	0,56	0,46	0,51	0,54	0,5	0,45	0,5	0,56	0,41
	LF	0,5	0,48	0,58	0,54	0,47	0,52	0,53	0,46	0,44	0,49	0,57	0,48
	LF	0,52	0,48	0,6	0,54	0,46	0,49	0,49	0,47	0,44	0,52	0,55	0,48
	LF	0,51	0,51	0,59	0,59	0,48	0,51	0,47	0,48	0,41	0,54	0,58	0,45
	**CV**	**8.51%**	**5.10%**	**3.93%**	**4.38%**	**4.99%**	**3.68%**	**5.62%**	**6.07%**	**3.17%**	**4.29%**	**2.08%**	**5.70%**
	RF	0,44	0,47	0,53	0,53	0,5	0,47	0,48	0,43	0,43	0,51	0,55	0,45
	RF	0,46	0,53	0,57	0,55	0,52	0,48	0,52	0,5	0,45	0,47	0,57	0,47
	RF	0,46	0,51	0,55	0,56	0,48	0,49	0,54	0,48	0,46	0,51	0,54	0,42
	RF	0,49	0,51	0,58	0,54	0,51	0,51	0,52	0,47	0,44	0,5	0,55	0,47
	RF	0,49	0,54	0,56	0,54	0,48	0,5	0,5	0,49	0,42	0,51	0,57	0,47
	RF	0,48	0,51	0,6	0,56	0,5	0,52	0,49	0,46	0,42	0,54	0,54	0,46
	**CV**	**4.26%**	**4.69%**	**4.30%**	**2.22%**	**3.21%**	**3.78%**	**4.38%**	**5.26%**	**3.74%**	**4.44%**	**2.47%**	**4.31%**
	LH	0,39	0,43	0,56	0,55	0,49	0,48	0,47	0,42	0,63	0,51	0,55	0,41
	LH	0,42	0,49	0,58	0,53	0,53	0,5	0,5	0,47	0,69	0,47	0,54	0,41
	LH	0,42	0,45	0,52	0,55	0,46	0,5	0,5	0,48	0,73	0,49	0,52	0,38

	LH	0,43	0,47	0,56	0,53	0,47	0,52	0,52	0,44	0,67	0,49	0,56	0,43
	LH	0,45	0,48	0,55	0,52	0,44	0,49	0,49	0,45	0,66	0,5	0,57	0,42
	LH	0,42	0,48	0,56	0,54	0,48	0,5	0,47	0,45	0,67	0,53	0,53	0,41
	**CV**	**4.60%**	**4.82%**	**3.56%**	**2.26%**	**6.40%**	**2.67%**	**3.95%**	**4.73%**	**4.94%**	**4.10%**	**3.43%**	**4.08%**
	RH	0,41	0,44	0,56	0,51	0,47	0,49	0,47	0,41	0,65	0,47	0,55	0,39
	RH	0,43	0,49	0,59	0,54	0,51	0,5	0,49	0,47	0,67	0,46	0,56	0,41
	RH	0,44	0,46	0,53	0,54	0,45	0,49	0,52	0,47	0,67	0,48	0,53	0,37
	RH	0,44	0,48	0,57	0,51	0,48	0,51	0,52	0,42	0,71	0,48	0,54	0,41
	RH	0,46	0,48	0,56	0,49	0,45	0,5	0,47	0,45	0,61	0,48	0,57	0,43
	RH	0,42	0,49	0,57	0,52	0,47	0,5	0,45	0,43	0,67	0,5	0,56	0,41
	**CV**	**4.04%**	**4.15%**	**3.49%**	**3.74%**	**4.72%**	**1.51%**	**5.91%**	**5.80%**	**4.92%**	**2.78%**	**2.67%**	**5.12%**
**Dog 13-21**		**13**	**14**	**15**	**16**	**17**	**18**	**19**	**20**	**21**			
	LF	0,45	0,54	0,42	0,54	0,57	0,54	0,49	0,58	0,55			
	LF	0,47	0,53	0,43	0,66	0,55	0,57	0,46	0,54	0,52			
	LF	0,49	0,43	0,5	0,65	0,59	0,58	0,49	0,59	0,55			
	LF	0,49	0,56	0,54	0,68	0,57	0,57	0,54	0,59	0,55			
	LF	0,47	0,55	0,52	0,71	0,59	0,55	0,51	0,58	0,57			
	LF	0,47	0,51	0,55	0,69	0,63	0,57	0,53	0,56	0,56			
	**CV**	**3.18%**	**9.10%**	**11.30%**	**9.20%**	**4.68%**	**2.67%**	**5.85%**	**3.43%**	**3.04%**			
	RF	0,44	0,54	0,45	0,6	0,57	0,55	0,49	0,58	0,56			
	RF	0,46	0,51	0,47	0,69	0,56	0,55	0,49	0,6	0,52			
	RF	0,48	0,45	0,51	0,68	0,58	0,6	0,5	0,62	0,56			
	RF	0,49	0,56	0,55	0,68	0,58	0,57	0,51	0,61	0,55			
	RF	0,47	0,57	0,59	0,7	0,58	0,56	0,48	0,65	0,55			
	RF	0,47	0,53	0,61	0,67	0,61	0,6	0,49	0,59	0,54			
	**CV**	**3.68%**	**8.20%**	**12.17%**	**5.34%**	**2.89%**	**4.05%**	**2.09%**	**4.08%**	**2.75%**			
	LH	0,34	0,5	0,44	0,55	0,53	0,55	0,59	0,58	0,54			
	LH	0,43	0,51	0,44	0,66	0,57	0,55	0,59	0,51	0,5			

LH	0,31	0,46	0,54	0,65	0,56	0,57	0,6	0,57	0,56
LH	0,46	0,54	0,53	0,64	0,55	0,52	0,56	0,55	0,53
LH	0,29	0,53	0,55	0,64	0,55	0,51	0,58	0,66	0,53
LH	0,43	0,49	0,56	0,63	0,6	0,52	0,58	0,57	0,52
**CV**	**19.12%**	**5.70%**	**10.81%**	**6.32%**	**4.23%**	**4.36%**	**2.34%**	**8.59%**	**3.77%**
RH	0,39	0,52	0,47	0,52	0,54	0,51	0,59	0,58	0,51
RH	0,46	0,51	0,45	0,66	0,57	0,57	0,57	0,6	0,48
RH	0,25	0,45	0,53	0,7	0,55	0,55	0,57	0,62	0,55
RH	0,47	0,52	0,55	0,7	0,55	0,52	0,57	0,52	0,48
RH	0,35	0,53	0,57	0,7	0,56	0,5	0,54	0,6	0,55
RH	0,45	0,47	0,59	0,66	0,59	0,55	0,55	0,52	0,52
**CV**	**21.47%**	**6.45%**	**10.58%**	**10.62%**	**3.19%**	**5.12%**	**3.12%**	**7.54%**	**6.11%**

Swing time (sec)

**Dog 1-12**	**1**	**2**	**3**	**4**	**5**	**6**	**7**	**8**	**9**	**10**	**11**	**12**
LF	0,29	0,27	0,3	0,28	0,3	0,22	0,26	0,26	0,25	0,28	0,26	0,23
LF	0,28	0,28	0,28	0,3	0,28	0,24	0,28	0,26	0,26	0,28	0,28	0,24
LF	0,29	0,28	0,32	0,32	0,28	0,25	0,28	0,26	0,22	0,26	0,27	0,24
LF	0,26	0,28	0,32	0,32	0,3	0,24	0,28	0,28	0,23	0,27	0,28	0,24
LF	0,26	0,32	0,3	0,3	0,28	0,26	0,28	0,28	0,24	0,28	0,28	0,25
LF	0,28	0,26	0,3	0,28	0,28	0,24	0,28	0,27	0,24	0,28	0,28	0,26
**CV**	**4.94%**	**7.25%**	**4.96%**	**5.96%**	**3.60%**	**5.50%**	**2.95%**	**3.66%**	**5.89%**	**3.04%**	**3.04%**	**4.24%**
RF	0,28	0,22	0,3	0,34	0,28	0,24	0,28	0,27	0,25	0,28	0,3	0,23
RF	0,29	0,25	0,3	0,28	0,27	0,24	0,3	0,28	0,25	0,28	0,26	0,25
RF	0,28	0,24	0,31	0,32	0,24	0,26	0,29	0,26	0,25	0,27	0,32	0,22
RF	0,28	0,26	0,32	0,3	0,26	0,25	0,3	0,29	0,24	0,26	0,31	0,25
RF	0,29	0,24	0,3	0,3	0,26	0,24	0,28	0,28	0,22	0,3	0,28	0,26
RF	0,28	0,25	0,32	0,32	0,25	0,24	0,27	0,28	0,25	0,29	0,34	0,24
**CV**	**1.82%**	**5.61%**	**3.19%**	**6.77%**	**5.44%**	**3.41%**	**4.22%**	**3.73%**	**4.98%**	**5.05%**	**9.47%**	**6.09%**

LH	0,33	0,29	0,34	0,3	0,31	0,24	0,3	0,3	0,37	0,28	0,28	0,28
LH	0,34	0,3	0,32	0,34	0,3	0,24	0,3	0,3	0,34	0,3	0,3	0,3
LH	0,34	0,3	0,32	0,34	0,3	0,26	0,34	0,3	0,36	0,3	0,33	0,26
LH	0,33	0,29	0,34	0,32	0,32	0,24	0,31	0,32	0,43	0,29	0,3	0,28
LH	0,34	0,31	0,35	0,32	0,32	0,25	0,3	0,32	0,36	0,32	0,27	0,3
LH	0,32	0,3	0,36	0,34	0,3	0,26	0,3	0,3	0,42	0,3	0,32	0,3
**CV**	**2.45%**	**2.52%**	**4.74%**	**5.00%**	**3.19%**	**3.96%**	**5.20%**	**3.37%**	**9.56%**	**4.46%**	**7.60%**	**5.70%**
RH	0,32	0,28	0,34	0,34	0,32	0,24	0,28	0,3	0,32	0,32	0,3	0,29
RH	0,34	0,31	0,31	0,32	0,33	0,24	0,32	0,3	0,38	0,3	0,28	0,29
RH	0,32	0,28	0,32	0,34	0,32	0,26	0,32	0,3	0,4	0,3	0,32	0,29
RH	0,32	0,28	0,32	0,34	0,32	0,26	0,32	0,32	0,4	0,32	0,32	0,31
RH	0,33	0,31	0,36	0,35	0,3	0,25	0,31	0,34	0,36	0,32	0,28	0,3
RH	0,32	0,29	0,32	0,36	0,3	0,26	0,3	0,32	0,37	0,32	0,32	0,3
**CV**	**2.57%**	**5.05%**	**5.59%**	**3.89%**	**3.89%**	**3.91%**	**5.20%**	**5.21%**	**8.06%**	**3.30%**	**6.48%**	**2.75%**

**Dog 13-21**		**13**	**14**	**15**	**16**	**17**	**18**	**19**	**20**	**21**
	LF	0,27	0,34	0,2		0,3	0,3	0,3	0,36	0,29
	LF	0,29	0,32	0,22	0,34	0,32	0,3	0,28	0,34	0,29
	LF	0,28	0,33	0,21	0,34	0,3	0,32	0,31	0,32	0,3
	LF	0,29	0,3	0,2	0,34	0,32	0,32	0,29	0,32	0,26
	LF	0,3	0,32	0,23	0,34	0,3	0,3	0,3	0,32	0,28
	LF	0,3	0,32	0,24	0,32	0,32	0,34	0,28	0,36	0,3
	**CV**	**4.05%**	**4.13%**	**7.54%**	**2.66%**	**3.53%**	**5.21%**	**4.13%**	**5.84%**	**5.25%**
	RF	0,26	0,32	0,18	0,32	0,34	0,32	0,31	0,3	0,26
	RF	0,28	0,32	0,18	0,34	0,32	0,3	0,27	0,3	0,3
	RF	0,28	0,32	0,19	0,36	0,34	0,3	0,32	0,26	0,29
	RF	0,3	0,3	0,19	0,34	0,32	0,31	0,32	0,3	0,28
	RF	0,3	0,3	0,19	0,32	0,32	0,3	0,31	0,29	0,3
	RF	0,34	0,32	0,19	0,32	0,32	0,3	0,3	0,31	0,3
	**CV**	**9.32%**	**3.30%**	**2.77%**	**4.90%**	**3.16%**	**2.74%**	**6.13%**	**5.97%**	**5.56%**

LH	0,34	0,34	0,18	0,34	0,36	0,32	0,26	0,38	0,3
LH	0,34	0,32	0,19	0,38	0,32	0,35	0,22	0,4	0,32
LH	0,2	0,3	0,19	0,4	0,3	0,36	0,28	0,42	0,32
LH	0,34	0,34	0,19	0,38	0,36	0,34	0,27	0,32	0,31
LH	0,26	0,34	0,18	0,4	0,36	0,34	0,26	0,36	0,32
LH	0,34	0,34	0,2	0,38	0,36	0,36	0,24	0,32	0,35
**CV**	**19.74%**	**5.07%**	**4.00%**	**5.77%**	**7.74%**	**4.40%**	**8.50%**	**11.27%**	**5.23%**
RH	0,24	0,34	0,16	0,3	0,32	0,36	0,27	0,34	0,34
RH	0,32	0,34	0,19	0,36	0,34	0,34	0,27	0,32	0,34
RH	0,21	0,32	0,16	0,38	0,34	0,36	0,3	0,34	0,33
RH	0,32	0,34	0,18	0,34	0,34	0,36	0,24	0,34	0,34
RH	0,21	0,36	0,18	0,34	0,34	0,34	0,29	0,4	0,34
RH	0,32	0,34	0,18	0,34	0,34	0,34	0,27	0,36	0,36
**CV**	**20.69%**	**3.72%**	**7.00%**	**7.74%**	**2.43%**	**3.13%**	**7.56%**	**7.88%**	**2.88%**

Stride Time (sec)

**Dog 1-12**		**1**	**2**	**3**	**4**	**5**	**6**	**7**	**8**	**9**	**10**	**11**	**12**
	LF	0,68	0,74	0,68	0,68	0,78	0,8	0,68	0,71	0,9	0,62		0,69
	LF	0,73	0,81	0,78	0,66	0,74	0,84	0,7	0,75	0,86	0,66	0,98	0,72
	LF	0,75	0,82	0,76	0,67	0,74	0,82	0,66	0,76	0,76	0,71	0,96	0,73
	LF	0,74	0,8	0,74	0,68	0,75	0,84	0,72	0,78	0,88	0,74	1,02	0,76
	LF	0,74	0,76	0,74	0,62	0,79	0,84	0,73	0,78	0,88	0,74	1,08	0,8
	LF	0,74	0,74	0,75	0,67	0,82	0,86	0,7	0,77	0,84	0,81	1,04	0,82
	**CV**	**3.47%**	**4.63%**	**4.55%**	**3.39%**	**4.19%**	**2.48%**	**3.67%**	**3.48%**	**5.87%**	**9.37%**	**4.70%**	**6.59%**
	RF	0,7	0,76	0,69	0,64	0,78	0,85	0,68	0,72	0,88	0,62	1	0,7
	RF	0,72	0,82	0,78	0,69	0,74	0,82	0,71	0,74	0,84	0,65	1,02	0,73
	RF	0,75	0,83	0,74	0,68	0,77	0,86	0,64	0,76	0,78	0,69	1,02	0,72
	RF	0,75	0,8	0,75	0,63	0,74	0,87	0,72	0,76	0,86	0,75	1,04	0,8
	RF	0,73	0,78	0,76	0,61	0,8	0,86	0,72	0,76	0,86	0,75	1,04	0,78

RF	0,76	0,75	0,74	0,71	0,82	0,88	0,7	0,8	0,86	0,81	0,98	0,78
**CV**	**3.07%**	**4.08%**	**4.05%**	**5.91%**	**4.14%**	**2.41%**	**4.43%**	**3.51%**	**4.14%**	**9.99%**	**2.30%**	**5.35%**
LH	0,7	0,77	0,72	0,98	0,76	0,82	0,7	0,76	0,86	0,61	0,96	0,72
LH	0,73	0,8	0,76	0,92	0,75	0,82	0,71	0,78	0,86	0,64	1,04	0,75
LH	0,76	0,84	0,75	1,1	0,78	0,85	0,64	0,47	0,76	0,73	1,02	0,76
LH	0,76	0,84	0,74	1,04	0,78	0,86	0,7	0,8	0,86	0,75	1,02	0,77
LH	0,74	0,78	0,76	0,96	0,8	0,84	0,72	0,47	0,88	0,75	1,06	0,8
LH	0,76	0,76	0,74	1,06	0,81	0,86	0,71	0,77	0,84	0,79	1,02	0,76
**CV**	**3.24%**	**4.37%**	**2.04%**	**6.72%**	**2.92%**	**2.18%**	**4.13%**	**23.61%**	**5.07%**	**9.91%**	**3.28%**	**3.43%**
RH	0,71	0,74	0,71	0,92	0,79	0,84	0,68	0,67	0,88	0,62	0,82	0,72
RH	0,72	0,81	0,77	1,03	0,76	0,84	0,71	0,78	0,86	0,65	1,02	0,76
RH	0,76	0,86	0,74	1,02	0,78	0,84	0,66	0,39	0,78	0,71	1,06	0,75
RH	0,76	0,84	0,72	1,02	0,8	0,86	0,71	0,79	0,86	0,75	1,06	0,76
RH	0,75	0,78	0,77	0,96	0,8	0,84	0,72	0,51	0,88	0,77	1,04	0,78
RH	0,76	0,74	0,76	1,03	0,82	0,88	0,7	0,76	0,84	0,79	0,98	0,76
**CV**	**3.03%**	**6.35%**	**3.47%**	**4.61%**	**2.58%**	**1.97%**	**3.23%**	**25.39%**	**4.40%**	**9.52%**	**9.19%**	**2.62%**

**Dog 13-21**		**13**	**14**	**15**	**16**	**17**	**18**	**19**	**20**	**21**
	LF	0,7	0,84	0,74	0,78	0,9	0,82	0,76	0,9	0,82
	LF	0,78	0,82	0,84	0,78	0,86	0,84	0,7	0,87	0,8
	LF	0,76	0,84	0,9	0,73	0,88	0,9	0,77	0,9	0,84
	LF	0,75	0,9	0,86	0,75	0,9	0,9	0,83	0,96	0,82
	LF	0,8	0,88	0,82	0,72	0,88	0,86	0,8	0,86	0,83
	LF	0,76	0,9	0,88	0,74	0,96	0,92	0,8	0,94	0,84
	**CV**	**4.45%**	**3.99%**	**6.73%**	**3.37%**	**3.84%**	**4.50%**	**5.80%**	**4.29%**	**1.84%**
	RF	0,68	0,8	0,86	0,76	0,92	0,84	0,77	0,82	0,8
	RF	0,77	0,85	0,84	0,79	0,9	0,82	0,71	0,88	0,8
	RF	0,74	0,86	0,9	0,72	0,92	0,9	0,78	0,84	0,84
	RF	0,78	0,9	0,86	0,76	0,88	0,87	0,82	0,93	0,82

RF	0,78	0,84	0,84	0,74	0,9	0,86	0,79	0,88	0,86
RF	0,74	0,92	0,9	0,75	0,92	0,9	0,77	0,9	0,8
**CV**	**5.10%**	**5.00%**	**3.15%**	**3.10%**	**1.80%**	**3.71%**	**4.67%**	**4.56%**	**3.09%**
LH	0,72	0,88	0,86	0,79	0,88	0,84	0,8	0,94	0,82
LH	0,78	0,92	0,86	0,82	0,88	0,87	0,72	0,96	0,82
LH	0,75	0,84	0,86	0,75	0,88	0,92	0,84	0,98	0,84
LH	0,74	0,9	0,86	0,78	0,9	0,86	0,82	0,88	0,83
LH	0,79	0,91	0,84	0,76	0,92	0,84	0,84	1	0,84
LH	0,78	0,92	0,9	0,76	0,94	0,88	0,8	0,9	0,85
**CV**	**3.63%**	**3.44%**	**2.28%**	**3.32%**	**2.81%**	**3.45%**	**5.55%**	**4.91%**	**1.45%**
RH	0,72	0,88	0,84	0,8	0,86	0,86	0,82	0,9	0,82
RH	0,79	0,9	0,84	0,83	0,88	0,86	0,81	0,92	0,81
RH	0,74	0,84	0,86	0,76	0,88	0,88	0,84	0,9	0,85
RH	0,74	0,9	0,84	0,8	0,9	0,88	0,8	0,86	0,82
RH	0,79	0,92	0,85	0,74	0,88	0,84	0,83	0,94	0,84
RH	0,78	0,86	0,88	0,76	0,92	0,9	0,8	0,88	0,88
**CV**	**3.99%**	**3.33%**	**1.88%**	**4.31%**	**2.33%**	**2.41%**	**2.00%**	**3.14%**	**3.09%**

Stride length (cm)

Dog 1-12		1	2	3	4	5	6	7	8	9	10	11	12
	LF	74,2	72,1	87,4	81,3	75,2	71,1	77,2	75,2	62	76,2	81,3	69,6
	LF	75,2	69,1	75,2	83,3	76,2	71,1	76,2	69,1	55,4	80,3	80,3	71,1
	LF	75,2	72,1	84,3	84,3	75,7	70,1	73,2	73,2	52,3	76,2	82,8	72,1
	LF	69,1	70,1	87,4	82,3	73,7	67,1	75,7	80,3	55,5	76,2	82,3	71,1
	LF	73,2	72,1	88,4	84,3	77,7	71,1	81,3	77,2	51,3	76,2	84,3	72,1
	LF	75,2	69,1	87,4	82,3	76,2	68,6	80,3	79,2	56,2	74,2	82,3	72,1
	**CV**	**3.23%**	**2.13%**	**5.89%**	**1.46%**	**1.74%**	**2.38%**	**3.91%**	**5.47%**	**6.78%**	**2.62%**	**1.65%**	**1.38%**
	RF	76,2	72,1	86,4	83,3	75,2	71,1	76,7	75,2	59,4	75,2	82,3	70,1
	RF	76,7	70,1	79,2	84,3	75,7	74,2	76,2	72,1	56,9	82,3	83,3	71,6

	RF	75,2	71,1	85,9	83,3	76,2	71,1	77,7	75,2	54,5	78,2	83,3	71,1
	RF	71,1	71,1	87,4	84,3	75,2	69,1	77,2	80,3	56,4	77,2	83,3	70,1
	RF	73,7	70,6	87,4	84,3	78,2	71,1	81,3	80,3	47,8	77,2	81,3	73,2
	RF	74,2	68,6	84,3	82,3	75,7	69,1	80,8	78,2	63,5	75,7	83,3	71,1
	**CV**	**2.72%**	**1.68%**	**3.65%**	**0.98%**	**1.48%**	**2.63%**	**2.79%**	**4.26%**	**9.29%**	**3.27%**	**1.01%**	**1.61%**
	LH	72,6	70,6	79,2	75,2	75,2	68,1	76,7	75,2	81,8	75,2	83,3	70,1
	LH	74,2	69,1	72,1	83,3	71,1	70,6	76,2	71,1	79,2	77,2	83,3	71,6
	LH	74,2	70,6	80,3	84,3	75,2	69,1	80,3	72,1	78,2	76,2	83,3	71,1
	LH	73,7	70,1	86,4	83,3	73,2	67,1	78,2	80,3	80,3	74,7	83,3	70,1
	LH	73,2	71,1	87,4	84,3	78,2	70,1	78,2	77,2	81,3	73,2	81,8	71,1
	LH	76,2	68,1	86,4	83,3	73,2	68,1	82,3	80,3	83,3	72,6	81,3	71,6
	**CV**	**1.67%**	**1.61%**	**7.25%**	**4.26%**	**3.27%**	**1.94%**	**2.91%**	**5.20%**	**2.28%**	**2.34%**	**1.11%**	**0.96%**
	RH	74,2	70,1	88,4	80,8	77,2	67,6	76,2	75,2	75,2	74,7	83,8	70,1
	RH	76,2	69,6	77,2	84,3	75,2	70,1	75,2	71,1	78,2	78,2	82,3	70,6
	RH	75,2	69,1	85,3	86,4	75,2	70,1	78,2	72,1	78,2	77,2	84,3	72,6
	RH	74,2	70,1	87,4	82,3	73,2	68,1	79,2	80,3	85,3	74,2	81,3	69,1
	RH	73,7	70,1	89,4	83,8	76,2	69,6	79,2	77,7	83,3	75,7	83,3	72,1
	RH	74,2	69,1	88,4	83,3	74,7	69,1	80,3	80,3	82,3	75,2	82,3	72,6
	**CV**	**1.23%**	**0.71%**	**5.28%**	**2.27%**	**1.80%**	**1.52%**	**2.52%**	**5.24%**	**4.74%**	**2.03%**	**1.34%**	**2.06%**
**Dog 13-21**		**13**	**14**	**15**	**16**	**17**	**18**	**19**	**20**	**21**			
	LF	69,1	82,3	52,2		85,3	87,4	77,2	86,4	74,2			
	LF	74,2	84,3	54,4	93,5	85,3	82,3	75,2	86,9	76,2			
	LF	75,2	81,8	45,7	96,5	81,3	79,2	79,2	86,4	77,2			
	LF	71,6	79,2	44,2	96,5	91,4	86,4	74,7	84,3	75,2			
	LF	76,2	81,3	46,7	98,6	92,5	87,4	72,1	87,4	74,7			
	LF	73,2	81,8	47,8	100,6	87,4	84,3	73,2	83,3	77,2			
	**CV**	**3.53%**	**2.01%**	**8.17%**	**2.73%**	**4.81%**	**3.87%**	**3.46%**	**1.88%**	**1.69%**			
	RF	67,1	82,3	53,3	103,6	87,4	87,4	76,7	89,4	73,2			

RF	74,2	82,8	52,5	97,5	84,3	86,4	74,7	89,4	76,2
RF	74,2	79,2	47,1	99,6	86,4	81,3	79,2	89,4	76,2
RF	71,1	79,2	47,1	95,5	90,4	83,8	76,2	85,9	75,7
RF	75,7	77,7	47,1	94,5	88,9	86,4	72,1	83,3	75,2
RF	74,2	83,3	47,4	99,6	87,4	85,3	74,7	86,9	77,2
**CV**	**4.33%**	**2.89%**	**6.05%**	**3.35%**	**2.39%**	**2.62%**	**3.15%**	**2.86%**	**1.79%**
LH	75,7	82,3	52,2	112,8	87,4	82,3	71,1	84,3	72,1
LH	72,6	82,3	53,5	97,5	86,4	80,3	64	87,4	74,2
LH	48,4	80,3	47,8	95,5	85,3	79,2	68,1	82,3	73,2
LH	71,6	79,2	47,4	96,5	89,4	84,3	72,6	90,4	75,2
LH	54,9	78,2	47,8	97,5	88,4	86,4	75,2	77,2	75,2
LH	73,7	82,3	48,1	99,6	84,3	84,3	72,1	86,4	75,2
**CV**	**17.38%**	**2.24%**	**5.38%**	**6.47%**	**2.20%**	**3.28%**	**5.58%**	**5.41%**	**1.75%**
RH	59,6	81,3	52,3	112,8	86,4	84,3	72,6	84,3	73,2
RH	72,1	81,3	54,4	92,5	82,3	81,3	70,1	86,4	75,7
RH	37,3	81,3	46,5	96,5	84,3	80,3	72,1	84,3	73,2
RH	70,6	78,2	47,1	94,5	89,4	84,3	72,1	90,4	76,2
RH	49,8	79,2	48,4	94,5	88,4	87,4	73,2	83,3	73,2
RH	71,1	85,3	47,4	99,6	85,3	84,3	72,1	85,3	74,2
**CV**	**23.55%**	**3.01%**	**6.54%**	**7.57%**	**3.06%**	**3.03%**	**1.45%**	**2.97%**	**1.83%**

Stride Velocity (cm/sec)

**Dog 1-12**		**1**	**2**	**3**	**4**	**5**	**6**	**7**	**8**	**9**	**10**	**11**	**12**
	LF	107,5	103,1	104	109,8	96,4	104,6	104,3	110,6	91,1	97,7	101,6	102,3
	LF	104,4	88,6	91,7	99,2	97,7	97,4	94,1	88,6	83,9	108,5	95,6	101,6
	LF	103	94,9	100,4	93,7	103,7	93,5	89,2	96,3	78,1	103	101	109,3
	LF	90,9	93,5	97,1	95,7	98,2	90,6	94,6	108,5	81,7	101,6	98	98,8
	LF	91,4	90,2	100,4	102,8	108	96,1	106,9	104,3	82,8	96,5	100,4	98,8
	LF	91,7	90,9	97,1	93,5	103	92,7	108,5	105,7	83,5	90,4	95,7	103,1
	**CV**	**7.76%**	**5.57%**	**4.25%**	**6.39%**	**4.42%**	**5.16%**	**8.01%**	**8.14%**	**5.10%**	**6.23%**	**2.71%**	**3.78%**

RF	108,9	106,1	108	96,9	98,9	101,6	100,9	109	92,9	96,4	96,8	103,1
RF	105,1	91	93,2	100,4	95,8	103	92,9	92,5	82,9	111,2	101,6	100,9
RF	104,4	96,1	99,8	92,6	105,8	94,8	93,6	101,6	80,2	101,6	96,9	111,1
RF	88,9	91,2	97,1	98,1	98,9	92,1	96,5	107	89,5	104,3	95,8	97,4
RF	94,4	90,5	104	100,4	105,7	97,4	104,2	105,6	78,3	96,5	94,5	101,6
RF	95,1	92,7	91,7	91,4	100,9	90,9	107,7	105,7	89,4	92,3	94,7	101,6
**CV**	**7.81%**	**6.33%**	**6.35%**	**3.99%**	**3.99%**	**5.12%**	**6.02%**	**5.73%**	**6.87%**	**6.76%**	**2.68%**	**4.45%**
LH	100,9	98,1	90,1	87,4	95,2	97,2	99,6	104,4	83,5	98,9	101,6	100,1
LH	98,9	88,6	78,4	96,9	86,7	96,7	95,3	93,6	86,1	103	101,6	100,9
LH	97,6	94,1	95,6	98,1	100,2	90,9	95,6	96,2	71,1	97,7	98	111,1
LH	95,7	94,7	96	96,9	93,8	88,2	93,1	108,5	77,2	95,7	96,9	100,1
LH	91,4	90	96	100,4	102,9	94,7	100,3	101,6	84,7	91,4	97,4	98,8
LH	100,3	87,3	93,9	92,6	96,3	89,6	108,3	108,5	78,6	89,7	94,5	100,9
**CV**	**3.61%**	**4.51%**	**7.50%**	**4.89%**	**5.85%**	**4.12%**	**5.51%**	**6.11%**	**7.06%**	**5.13%**	**2.84%**	**4.44%**
RH	103	97,4	100,4	96,2	96,5	95,2	103	105,9	81,7	94,5	99,8	103,1
RH	100,3	88,1	85,8	100,4	90,6	97,4	92,8	92,4	76	102,9	98	99,5
RH	100,2	93,4	101,6	100,4	98,9	92,2	91	97,5	76,7	99	100,4	110,1
RH	97,6	94,7	97,1	98	91,4	89,6	94,3	111,5	83,7	92,7	94,5	97,3
RH	94,4	88,7	97,2	98,6	103	92,8	101,6	100,9	86,8	94,6	99,2	100,2
RH	97,6	88,6	102,8	94,7	98,3	90,9	108,5	105,6	79,9	91,7	93,5	103,8
**CV**	**3.00%**	**4.24%**	**6.33%**	**2.33%**	**4.91%**	**3.07%**	**6.98%**	**6.65%**	**5.13%**	**4.43%**	**2.96%**	**4.39%**

**Dog 13-21**		**13**	**14**	**15**	**16**	**17**	**18**	**19**	**20**	**21**
	LF	97,3	91,4	84,1		94,8	106,6	101,6	96	90,4
	LF	98,9	98,1	82,4	95,4	99,2	98	107,4	99,8	95,3
	LF	98,9	107,6	64,7	100,5	92,4	88,1	102,9	96	91,9
	LF	91,8	90,1	59,7	94,6	101,6	96	90	87,8	91,7
	LF	97,7	92,4	63,2	91,3	105,1	101,6	90,2	101,6	90
	LF	95	97,4	59,2	96,7	91	91,7	91,4	88,6	91,9

**CV**	**2.85%**	**6.73%**	**16.45%**	**3.49%**	**5.68%**	**6.88%**	**7.84%**	**5.98%**	**2.03%**
RF	93,1	93,5	86	103,6	95	104	99,6	109	91,4
RF	100,2	98,6	81,2	95,6	93,7	105,3	105,2	101,6	95,3
RF	97,6	101,6	67,9	97,6	93,9	90,3	101,6	106,4	90,7
RF	93,6	92,1	63	91,8	102,8	96,3	92,9	92,3	92,3
RF	99,6	90,4	63	90,9	98,8	100,4	91,3	94,7	87,4
RF	92,7	96,9	58,3	101,6	95	94,8	97	96,5	96,5
**CV**	**3.55%**	**4.45%**	**15.94%**	**5.29%**	**3.71%**	**5.85%**	**5.39%**	**6.69%**	**3.56%**
LH	99,6	95,7	85	117,5	99,3	98	88,9	89,7	88
LH	93,1	95,7	83,6	93,8	98,1	92,3	88,9	91	90,4
LH	103,8	105,6	65,1	93,6	97	86,1	81	84	87,1
LH	89,5	92,1	63,5	94,6	99,3	98,1	88,6	102,8	90,6
LH	116,7	88,9	63,7	92	96,1	102,8	89,5	77,2	89,5
LH	95,7	98	61,1	97,6	89,7	95,8	90,2	96	88,5
**CV**	**9.72%**	**5.93%**	**15.50%**	**9.82%**	**3.73%**	**6.01%**	**3.87%**	**9.93%**	**1.56%**
RH	88,5	92,4	84,4	137,5	100,4	98,1	88,6	93,7	89,2
RH	92,5	94,5	83,6	90,6	93,5	94,5	86,5	93,9	93,4
RH	96,3	104,2	65	91,1	95,8	91,2	85,9	93,7	86,1
RH	89,4	91	62,5	89,1	99,3	95,8	90,2	105,1	92,9
RH	97	90,1	63,2	90,9	100,4	104	88,1	88,6	87,1
RH	93,6	101,6	59,8	101,6	92,8	93,7	90,2	97	84,3
**CV**	**3.75%**	**6.15%**	**16.01%**	**18.83%**	**3.56%**	**4.62%**	**2.05%**	**5.76%**	**4.17%**

Peak vertical force (%BW)

	**1**	**2**	**3**	**4**	**5**	**6**	**7**	**8**	**9**	**10**	**11**	**12**
LF	52,94	48,842	86,747	74,459	53,436	78,281	62,59	56,325	71,008	60,005	81,684	51,515
LF	54,609	56,602	100,352	69,272	60,349	75,033	70,298	53,435	74,767	58	90,405	45,582
LF	60,472	54,98	96,885	74,155	55,709	87,07	71	62,185	79,633	69,535	75,735	40,115
LF	66,477	50,695	83,511	75,873	52,721	77,409	62,669	62,605	64,864	52,769	78,152	44,248

	LF	61,439	50,257	80,789	72,275	53,423	73,571	60,054	55,42	69,876	57,647	73,69	47,331
	LF	68,238	53,799	102,743	82,863	60,652	73,708	58,507	57,313	63,632	66,039	72,088	46,738
	**CV**	**10.11%**	**5.79%**	**10.15%**	**6.08%**	**6.41%**	**6.53%**	**8.18%**	**6.44%**	**8.52%**	**10.07%**	**8.51%**	**8.18%**
	RF	63,41	65,021	84,135	75,747	63,154	69,371	49,563	52,43	56,2	53,52	46,497	43,398
	RF	68,459	78,188	87,422	89,887	67,242	77,344	45,24	57,748	62,875	48,664	76,393	43,845
	RF	84,304	72,839	97,32	79,666	64,241	75,781	50,88	53,395	62,042	54,199	49,218	42,688
	RF	67,022	75,546	94,002	71,206	68,866	77,391	44,756	55,919	71,044	59,592	47,895	41,834
	RF	64,264	77,766	89,403	79,061	65,133	74,853	44,579	57,206	54,528	63,65	83,272	52,205
	RF	70,189	77,853	91,061	69,274	66,103	79,834	48,931	58,327	66,443	60,729	56,437	43,824
	**CV**	**10.97%**	**6.82%**	**5.19%**	**9.50%**	**3.15%**	**4.70%**	**5.88%**	**4.34%**	**9.95%**	**9.78%**	**26.56%**	**8.49%**
	LH	25,848	33,078	45,921	42,539	34,084	55,125	34,87	31,681	69,3	38,54	53,317	21,32
	LH	30,69	41,285	61,983	42,837	44,964	50,682	39,091	33,691	81,333	34,696	77,099	23,759
	LH	29,373	36,248	46,98	39,735	32,62	52,433	34,576	32,561	71,432	37,019	45,858	20,596
	LH	32,425	39,227	50,095	54,219	32,352	47,606	37,795	33,637	67,532	33,914	51,046	21,833
	LH	29,193	39,111	56,06	44,534	35,327	50,2	31,67	35,43	58,512	39,51	76,535	22,819
	LH	38,005	36,126	46,221	39,791	32,878	53,244	28,985	33,233	64,224	39,857	39,421	22,312
	**CV**	**13.22%**	**7.81%**	**12.70%**	**12.22%**	**13.65%**	**5.10%**	**10.88%**	**3.78%**	**11.13%**	**6.71%**	**27.83%**	**5.06%**
	RH	26,443	38,141	48,099	38,434	32,917	50,342	37,32	31,417	73,049	32,954	48,081	24,114
	RH	29,79	38,474	53,937	51,452	32,847	49,974	31,675	32,89	67,542	31,95	73,244	25,615
	RH	29,91	39,018	45,607	40,686	31,909	59,551	38,308	34,541	61,415	36,31	59,251	21,484
	RH	35,88	36,754	54,059	40,199	37,596	54,692	35,417	29,027	75,696	34,34	47,541	23,541
	RH	30,108	42,048	53,434	45,994	32,221	52,961	30,737	34,776	62,885	38,941	81,743	21,826
	RH	29,73	43,3	49,33	36,729	29,01	51,148	31,592	32,599	75,138	40,242	49,44	23,04
	**CV**	**10.09%**	**6.34%**	**7.04%**	**12.98%**	**8.47%**	**6.81%**	**9.55%**	**6.55%**	**9.02%**	**9.26%**	**24.28%**	**6.55%**
**Dog 13-21**		**13**	**14**	**15**	**16**	**17**	**18**	**19**	**20**	**21**			
	LF	53,462	77,929	34,5	95,218	74,814	76,54	85,151	86,549	81,255			
	LF	53,037	78,491	37,519	140,987	72,343	76,654	87,307	91,542	74,927			
	LF	56,242	60,338	49,878	126,613	76,963	83,107	86,556	80,737	76,769			

LF	56,179	68,393	47,253	133,064	67,062	81,593	87,37	91,702	79,623
LF	51,455	69,172	37,202	143,42	86,067	83,98	91,643	92,397	82,053
LF	47,839	68,98	42,886	140,825	73,945	69,136	95,411	90,874	78,297
**CV**	**5.95%**	**9.63%**	**14.78%**	**13.97%**	**8.35%**	**7.11%**	**4.34%**	**5.10%**	**3.44%**
RF	44,777	76,303	44,097	107,809	65,92	75,199	86,742	84,024	83,537
RF	45,306	67,418	46,658	134,631	73,711	72,968	77,761	105,817	79,241
RF	52,357	61,885	40,862	142,631	70,999	77,727	89,789	97,259	84,626
RF	47,545	68,639	49,578	126,704	78,047	80,873	80,85	105,893	79,351
RF	53,449	74,225	55,924	137,242	79,829	80,787	80,299	97,599	79,142
RF	49,982	70,883	59,112	123,644	74,475	82,584	82,917	97,211	72,725
**CV**	**7.40%**	**7.37%**	**14.19%**	**9.63%**	**6.77%**	**4.76%**	**5.37%**	**8.17%**	**5.27%**
LH	26,59	35,257	28,46	72,731	42,8	53,954	56,471	44,125	46,38
LH	28,383	33,446	29,289	70,591	41,455	50,461	64,834	34,434	41,766
LH	16,454	30,706	33,989	73,483	43,944	49,741	67,381	37,517	43,719
LH	30,183	35,847	32,928	61,657	39,577	37,74	56,378	46,283	40,128
LH	16,92	39,238	38,017	61,351	39,043	36,147	58,104	49,049	41,786
LH	28,609	31,154	32,022	61,058	41,043	40,335	57,65	44,624	44,053
**CV**	**25.19%**	**9.34%**	**10.65%**	**9.06%**	**4.51%**	**16.88%**	**7.89%**	**13.01%**	**5.13%**
RH	25,517	31,755	50,691	61,484	47,897	45,497	48,088	43,241	37,426
RH	31,335	31,215	43,973	68,787	40,054	46,92	45,159	55,425	35,64
RH	18,468	31,263	47,924	85,306	45,203	42,047	54,493	47,97	38,219
RH	32,354	37,134	48,968	74,235	45,419	45,886	53,747	33,58	37,907
RH	22,935	38,548	48,959	79,432	36,529	44,969	48,98	42,819	38,571
RH	33,746	33,609	55,807	74,063	42,174	55,016	44,526	35,158	38,513
**CV**	**22.12%**	**9.41%**	**7.83%**	**11.18%**	**9.66%**	**9.38%**	**8.54%**	**18.86%**	**2.92%**

Peak vertical force (N)

	**1**	**2**	**3**	**4**	**5**	**6**	**7**	**8**	**9**	**10**	**11**	**12**
LF	185,532	160,76	207,113	205,034	155,741	227,55	171,269	177,897	279,448	159,068	201,71	165,27

LF	169,154	160,067	221,388	185,906	178,917	211,421	174,291	142,762	274,013	159,118	214,626	142,453
LF	187,707	167,828	242,385	188,086	183,542	237,244	175,758	165,275	269,053	176,211	180,098	149,066
LF	198,968	145,31	196,496	199,137	165,259	206,412	156,383	191,521	245,406	139,336	181,441	130,365
LF	176,05	143,819	185,495	196,375	173,033	206,499	158,352	156,119	263,131	141,567	175,225	137,414
LF	189,255	149,068	235,425	195,555	198,407	201,146	168,564	154,931	255,231	161,924	170,701	140,868
**CV**	**5.69%**	**6.32%**	**10.38%**	**3.63%**	**8.44%**	**6.59%**	**4.90%**	**10.67%**	**4.75%**	**8.82%**	**9.13%**	**8.32%**
RF	192,781	178,888	204,636	199,942	170,353	205,847	148,933	167,169	223,917	141,364	107,062	137,605
RF	202,022	201,768	217,577	249,98	169,648	224,137	125,551	154,709	238,585	132,448	201,187	135,9
RF	244,796	188,238	233,848	214,649	181,867	213,137	138,632	147,406	224,968	139,935	115,519	157,83
RF	189,167	199,609	214,91	194,278	180,857	211,793	126,008	165,036	272,759	163,85	105,776	125,59
RF	176,313	197,076	222,254	216,624	180,131	208,076	132,978	159,468	233,555	168,522	207,114	158,625
RF	207,498	194,512	208,197	188,005	181,779	221,015	150,67	181,051	263,102	151,733	135,481	140,683
**CV**	**11.65%**	**4.39%**	**4.82%**	**10.61%**	**3.27%**	**3.36%**	**7.99%**	**7.13%**	**8.41%**	**9.55%**	**32.20%**	**9.14%**
LH	107,075	98,646	110,645	102,102	97,818	150,08	90,281	99,474	157,156	106,229	125,864	86,302
LH	114,72	108,248	146,302	105,294	112,741	136,244	94,431	87,926	155,144	106,574	209,799	90,864
LH	106,956	104,818	114,787	90,165	104,743	136,31	85,897	87,519	135,58	102,125	111,668	93,378
LH	110,886	110,439	117,798	135,705	97,438	117,611	90,814	100,169	144,629	102,592	113,784	80,728
LH	98,866	108,297	128,802	115,996	125,428	132,366	82,147	103,183	124,554	107,755	198,316	88,097
LH	133,846	99,03	109,922	95,942	97,033	137,832	79,504	98,234	144,478	102,721	92,717	90,259
**CV**	**10.62%**	**4.80%**	**11.53%**	**15.20%**	**10.73%**	**7.75%**	**6.52%**	**6.95%**	**8.51%**	**2.35%**	**34.74%**	**5.00%**
RH	100,631	117,536	129,952	99,232	92,701	135,111	104,512	96,945	155,792	96,008	112,186	99,634
RH	111,172	103,143	123,138	121,576	80,48	127,774	79,999	89,793	146,262	103,326	188,055	97,721
RH	105,434	116,332	129,39	92,844	101,381	153,723	99,403	98,466	134,136	105,291	140,802	102,101
RH	117,966	107,148	129,786	101,977	102,027	138,642	88,829	89,179	147,966	96,646	109,013	83,413
RH	96,305	114,342	129,397	118,777	102,192	136,648	90,631	100,465	149,561	110,884	194,869	80,207
RH	105,126	115,789	116,868	86,043	87,765	128,592	93,09	97,713	141,88	112,532	111,614	88,891
**CV**	**7.22%**	**5.19%**	**4.24%**	**13.67%**	**9.57%**	**6.87%**	**9.21%**	**4.98%**	**5.04%**	**6.66%**	**27.69%**	**9.91%**
	**13**	**14**	**15**	**16**	**17**	**18**	**19**	**20**	**21**			

LF	175,425	196,3	121,435	267,637	171,707	191,243	265,892	218,978	205,245
LF	157,137	211,491	128,982	285,202	177,581	176,29	279,158	251,982	200,256
LF	162,719	199,101	143,184	269,601	165,977	197,377	260,189	209,639	189,194
LF	153,966	174,133	120,753	285,306	158,946	185,827	234,33	228,611	199,23
LF	147,932	182,485	103,189	282,487	198,856	216,733	271,295	241,93	192,153
LF	136,262	193,495	109,097	289,455	156,949	161,49	276,763	241,948	192,536
**CV**	**8.53%**	**6.79%**	**11.76%**	**3.24%**	**8.97%**	**10.00%**	**6.19%**	**6.89%**	**3.10%**
RF	150,402	195,268	154,75	245,196	151,229	176,283	254,702	214,737	212,594
RF	134,506	178,009	168,165	273,188	177,07	175,171	222,912	248,273	203,971
RF	157,997	195,282	123,133	297,065	168,774	172,122	247,569	233,257	205,424
RF	131,866	171,544	145,31	268,486	181,674	192,001	240,122	246,749	195,767
RF	155,9	182,479	151,002	284,107	185,726	186,606	243,61	208,748	194,029
RF	147,683	189,023	142,673	270,072	167,115	181,547	248,625	236,589	177,773
**CV**	**7.45%**	**5.20%**	**10.12%**	**6.35%**	**7.23%**	**4.19%**	**4.52%**	**7.08%**	**6.11%**
LH	116,399	95,014	86,685	175,563	105,241	135,814	135,584	100,368	128,413
LH	105,301	89,637	86,678	134,609	97,419	130,288	146,046	90,357	116,468
LH	71,156	92,714	90,311	142,746	105,188	111,904	152,129	91,2	108,244
LH	109,48	90,486	85,469	119,908	93,743	99,814	135,183	113,141	109,964
LH	83,759	96,857	92,424	118,679	94,487	105,099	140,698	100,346	112,948
LH	114,888	83,309	79,579	125,6	88,089	104,352	132,961	104,277	121,417
**CV**	**18.43%**	**5.23%**	**5.09%**	**15.68%**	**6.98%**	**13.05%**	**5.28%**	**8.51%**	**6.54%**
RH	103,905	83,02	160,706	168,635	118,438	130,692	121,511	107,02	102,181
RH	112,408	87,368	134,153	129,701	91,96	117,371	118,442	122,378	100,737
RH	106,505	107,991	123,112	157,484	108,476	104,321	134,828	105,233	94,562
RH	103,098	93,169	124,665	136,279	108,037	124,868	141,651	87,299	107,614
RH	96,081	99,107	126,95	139,187	83,295	132,331	130,346	99,017	93,787
RH	118,526	103,805	133,261	143,805	92,996	139,539	123,16	90,169	101,053
**CV**	**7.33%**	**10.07%**	**10.40%**	**9.96%**	**13.10%**	**10.02%**	**6.92%**	**12.54%**	**5.15%**

Vertical Impulse (%BW*sec)

**Dog 1-12**		**1**	**2**	**3**	**4**	**5**	**6**	**7**	**8**	**9**	**10**	**11**	**12**
	LF	17,1	14,2	20,6	21,1	17,6	17	208,6	17,5	18,1	20,4	20,8	19,5
	LF	17,7	16,5	23,8	19,6	19,9	16,3	234,3	16,6	19,1	19,7	23	17,2
	LF	19,6	16	23	21	18,3	18,9	236,7	19,3	20,4	23,6	19,3	15,2
	LF	21,5	14,8	19,8	21,5	17,3	16,8	208,9	19,5	16,6	17,9	19,9	16,7
	LF	19,9	14,6	19,2	20,5	17,6	16	200,2	17,2	17,9	19,6	18,7	17,9
	LF	22,1	15,7	24,4	23,5	20	16	195	17,8	16,3	22,4	18,3	17,7
	**CV**	**10.13%**	**5.88%**	**10.13%**	**6.14%**	**6.55%**	**6.49%**	**8.19%**	**6.50%**	**8.51%**	**10.04%**	**8.58%**	**8.19%**
	RF	20,5	18,9	20	21,5	20,8	15,1	165,2	16,3	14,4	18,2	11,8	16,4
	RF	22,2	22,8	20,7	25,5	22,1	16,8	150,8	18	16,1	16,5	19,4	16,6
	RF	27,3	21,2	23,1	22,6	21,1	16,4	169,6	16,6	15,9	18,4	12,5	16,1
	RF	21,7	22	22,3	20,2	22,7	16,8	149,2	17,4	18,2	20,3	12,2	15,8
	RF	20,8	22,7	21,2	22,4	21,4	16,2	148,6	17,8	13,9	21,6	21,2	19,7
	RF	22,7	22,7	21,6	19,6	21,7	17,3	163,1	18,1	17	20,6	14,4	16,6
	**CV**	**11.00%**	**6.95%**	**5.18%**	**9.55%**	**3.20%**	**4.60%**	**5.88%**	**4.35%**	**10.05%**	**9.80%**	**26.57%**	**8.43%**
	LH	8,4	9,6	10,9	12	11,2	12	116,2	9,8	17,7	13,1	13,6	8,1
	LH	9,9	12	14,7	12,1	14,8	11	130,3	10,5	20,8	11,8	19,6	9
	LH	9,5	10,6	11,1	11,3	10,7	11,4	115,3	10,1	18,3	12,6	11,7	7,8
	LH	10,5	11,4	11,9	15,4	10,6	10,3	126	10,5	17,3	11,5	13	8,2
	LH	9,5	11,4	13,3	12,6	11,6	10,9	105,6	11	15	13,4	19,5	8,6
	LH	12,3	10,5	11	11,3	10,8	11,6	96,6	10,3	16,4	13,5	10	8,4
	**CV**	**13.10%**	**7.82%**	**12.68%**	**12.28%**	**13.80%**	**5.33%**	**10.89%**	**3.94%**	**11.09%**	**6.65%**	**27.82%**	**5.01%**
	RH	8,6	11,1	11,4	10,9	10,8	10,9	124,4	9,8	18,7	11,2	12,2	9,1
	RH	9,6	11,2	12,8	14,6	10,8	10,8	105,6	10,2	17,3	10,9	18,6	9,7
	RH	9,7	11,4	10,8	11,5	10,5	12,9	127,7	10,7	15,7	12,3	15,1	8,1
	RH	11,6	10,7	12,8	11,4	12,4	11,9	118,1	9	19,3	11,7	12,1	8,9
	RH	9,7	12,3	12,7	13	10,6	11,5	102,5	10,8	16,1	13,2	20,8	8,2
	RH	9,6	12,6	11,7	10,4	9,5	11,1	105,3	10,1	19,2	13,7	12,6	8,7

	**CV**	**9.98%**	**6.40%**	**7.10%**	**13.02%**	**8.69%**	**6.87%**	**9.54%**	**6.51%**	**8.93%**	**9.15%**	**24.26%**	**6.77%**

**Dog 13-21**		**13**	**14**	**15**	**16**	**17**	**18**	**19**	**20**	**21**
	LF	20	23,4	10,3	20	21,2	22,4	19,7	22,1	21,2
	LF	19,9	23,5	11,3	29,6	20,5	22,5	20,2	23,3	19,6
	LF	21,1	18,1	15	26,6	21,8	24,4	20,1	20,6	20,1
	LF	21,1	20,5	14,2	28	19	23,9	20,2	23,4	20,8
	LF	19,3	20,7	11,2	30,2	24,4	24,6	21,2	23,6	21,5
	LF	17,9	20,7	12,9	29,6	20,9	20,3	22,1	23,2	20,5
	**CV**	**6.06%**	**9.62%**	**14.89%**	**14.00%**	**8.39%**	**7.07%**	**4.34%**	**5.09%**	**3.41%**
	RF	16,8	22,9	13,2	22,7	18,7	22	20,1	21,4	21,8
	RF	17	20,2	14	28,3	20,9	21,4	18	27	20,7
	RF	19,6	18,6	12,3	30	20,1	22,8	20,8	24,8	22,1
	RF	17,8	20,6	14,9	26,6	22,1	23,7	18,7	27	20,7
	RF	20	22,3	16,8	28,9	22,6	23,7	18,6	24,9	20,7
	RF	18,7	21,3	17,7	26	11,6	11,8	13,4	11,4	11,5
	**CV**	**7.30%**	**7.37%**	**14.11%**	**9.62%**	**20.89%**	**21.78%**	**14.24%**	**26.05%**	**20.47%**
	LH	10	10,6	8,5	15,3	12,1	15,8	13,1	11,2	12,1
	LH	10,6	10	8,8	14,8	11,7	14,8	15	8,8	10,9
	LH	6,2	9,2	10,2	15,4	12,4	14,6	15,6	9,6	11,4
	LH	11,3	10,8	9,9	13	11,2	11,1	13,1	11,8	10,5
	LH	6,3	11,8	11,4	12,9	11,1	10,6	13,5	12,5	10,9
	LH	10,7	9,3	9,6	12,8	11,6	11,8	13,4	11,4	11,5
	**CV**	**25.15%**	**9.62%**	**10.71%**	**8.98%**	**4.31%**	**16.83%**	**7.70%**	**12.87%**	**5.06%**
	RH	9,6	9,5	15,2	12,9	13,6	13,3	11,1	11	9,8
	RH	11,7	9,4	13,2	14,5	11,3	13,7	10,5	14,1	9,3
	RH	6,9	9,4	14,4	17,9	12,8	12,3	12,6	12,2	10
	RH	12,1	11,1	14,7	15,6	12,9	13,4	12,5	8,6	9,9
	RH	8,6	11,6	14,7	16,7	10,3	13,2	11,4	10,9	10,1

	RH	12,7	10,1	16,7	15,6	11,9	16,1	10,3	9	10,1		
	**CV**	**22.17%**	**9.37%**	**7.70%**	**11.14%**	**9.95%**	**9.38%**	**8.56%**	**18.60%**	**3.05%**		

Vertical Impulse (N*sec)

**Dog 1-12**		**1**	**2**	**3**	**4**	**5**	**6**	**7**	**8**	**9**	**10**	**11**	**12**
	LF	52,94	48,842	86,747	74,459	53,436	78,281	62,59	56,325	71,008	60,005	81,684	51,515
	LF	54,609	56,602	100,352	69,272	60,349	75,033	70,298	53,435	74,767	58	90,405	45,582
	LF	60,472	54,98	96,885	74,155	55,709	87,07	71	62,185	79,633	69,535	75,735	40,115
	LF	66,477	50,695	83,511	75,873	52,721	77,409	62,669	62,605	64,864	52,769	78,152	44,248
	LF	61,439	50,257	80,789	72,275	53,423	73,571	60,054	55,42	69,876	57,647	73,69	47,331
	LF	68,238	53,799	102,743	82,863	60,652	73,708	58,507	57,313	63,632	66,039	72,088	46,738
	**CV**	**10.11%**	**5.79%**	**10.15%**	**6.08%**	**6.41%**	**6.53%**	**8.18%**	**6.44%**	**8.52%**	**10.07%**	**8.51%**	**8.18%**
	RF	63,41	65,021	84,135	75,747	63,154	69,371	49,563	52,43	56,2	53,52	46,497	43,398
	RF	68,459	78,188	87,422	89,887	67,242	77,344	45,24	57,748	62,875	48,664	76,393	43,845
	RF	84,304	72,839	97,32	79,666	64,241	75,781	50,88	53,395	62,042	54,199	49,218	42,688
	RF	67,022	75,546	94,002	71,206	68,866	77,391	44,756	55,919	71,044	59,592	47,895	41,834
	RF	64,264	77,766	89,403	79,061	65,133	74,853	44,579	57,206	54,528	63,65	83,272	52,205
	RF	70,189	77,853	91,061	69,274	66,103	79,834	48,931	58,327	66,443	60,729	56,437	43,824
	**CV**	**10.97%**	**6.82%**	**5.19%**	**9.50%**	**3.15%**	**4.70%**	**5.88%**	**4.34%**	**9.95%**	**9.78%**	**26.56%**	**8.49%**
	LH	25,848	33,078	45,921	42,539	34,084	55,125	34,87	31,681	69,3	38,54	53,317	21,32
	LH	30,69	41,285	61,983	42,837	44,964	50,682	39,091	33,691	81,333	34,696	77,099	23,759
	LH	29,373	36,248	46,98	39,735	32,62	52,433	34,576	32,561	71,432	37,019	45,858	20,596
	LH	32,425	39,227	50,095	54,219	32,352	47,606	37,795	33,637	67,532	33,914	51,046	21,833
	LH	29,193	39,111	56,06	44,534	35,327	50,2	31,67	35,43	58,512	39,51	76,535	22,819
	LH	38,005	36,126	46,221	39,791	32,878	53,244	28,985	33,233	64,224	39,857	39,421	22,312
	**CV**	**13.22%**	**7.81%**	**12.70%**	**12.22%**	**13.65%**	**5.10%**	**10.88%**	**3.78%**	**11.13%**	**6.71%**	**27.83%**	**5.06%**
	RH	26,443	38,141	48,099	38,434	32,917	50,342	37,32	31,417	73,049	32,954	48,081	24,114
	RH	29,79	38,474	53,937	51,452	32,847	49,974	31,675	32,89	67,542	31,95	73,244	25,615
	RH	29,91	39,018	45,607	40,686	31,909	59,551	38,308	34,541	61,415	36,31	59,251	21,484

	RH	35,88	36,754	54,059	40,199	37,596	54,692	35,417	29,027	75,696	34,34	47,541	23,541
	RH	30,108	42,048	53,434	45,994	32,221	52,961	30,737	34,776	62,885	38,941	81,743	21,826
	RH	29,73	43,3	49,33	36,729	29,01	51,148	31,592	32,599	75,138	40,242	49,44	23,04
	**CV**	**10.09%**	**6.34%**	**7.04%**	**12.98%**	**8.47%**	**6.81%**	**9.55%**	**6.55%**	**9.02%**	**9.26%**	**24.28%**	**6.55%**
**Dog 13-21**		**13**	**14**	**15**	**16**	**17**	**18**	**19**	**20**	**21**			
	LF	53,462	77,929	34,5	95,218	74,814	76,54	85,151	86,549	81,255			
	LF	53,037	78,491	37,519	140,987	72,343	76,654	87,307	91,542	74,927			
	LF	56,242	60,338	49,878	126,613	76,963	83,107	86,556	80,737	76,769			
	LF	56,179	68,393	47,253	133,064	67,062	81,593	87,37	91,702	79,623			
	LF	51,455	69,172	37,202	143,42	86,067	83,98	91,643	92,397	82,053			
	LF	47,839	68,98	42,886	140,825	73,945	69,136	95,411	90,874	78,297			
	**CV**	**5.95%**	**9.63%**	**14.78%**	**13.97%**	**8.35%**	**7.11%**	**4.34%**	**5.10%**	**3.44%**			
	RF	44,777	76,303	44,097	107,809	65,92	75,199	86,742	84,024	83,537			
	RF	45,306	67,418	46,658	134,631	73,711	72,968	77,761	105,817	79,241			
	RF	52,357	61,885	40,862	142,631	70,999	77,727	89,789	97,259	84,626			
	RF	47,545	68,639	49,578	126,704	78,047	80,873	80,85	105,893	79,351			
	RF	53,449	74,225	55,924	137,242	79,829	80,787	80,299	97,599	79,142			
	RF	49,982	70,883	59,112	123,644	74,475	82,584	82,917	97,211	72,725			
	**CV**	**7.40%**	**7.37%**	**14.19%**	**9.63%**	**6.77%**	**4.76%**	**5.37%**	**8.17%**	**5.27%**			
	LH	26,59	35,257	28,46	72,731	42,8	53,954	56,471	44,125	46,38			
	LH	28,383	33,446	29,289	70,591	41,455	50,461	64,834	34,434	41,766			
	LH	16,454	30,706	33,989	73,483	43,944	49,741	67,381	37,517	43,719			
	LH	30,183	35,847	32,928	61,657	39,577	37,74	56,378	46,283	40,128			
	LH	16,92	39,238	38,017	61,351	39,043	36,147	58,104	49,049	41,786			
	LH	28,609	31,154	32,022	61,058	41,043	40,335	57,65	44,624	44,053			
	**CV**	**25.19%**	**9.34%**	**10.65%**	**9.06%**	**4.51%**	**16.88%**	**7.89%**	**13.01%**	**5.13%**			
	RH	25,517	31,755	50,691	61,484	47,897	45,497	48,088	43,241	37,426			
	RH	31,335	31,215	43,973	68,787	40,054	46,92	45,159	55,425	35,64			

	RH	18,468	31,263	47,924	85,306	45,203	42,047	54,493	47,97	38,219		
	RH	32,354	37,134	48,968	74,235	45,419	45,886	53,747	33,58	37,907		
	RH	22,935	38,548	48,959	79,432	36,529	44,969	48,98	42,819	38,571		
	RH	33,746	33,609	55,807	74,063	42,174	55,016	44,526	35,158	38,513		
	**CV**	**22.12%**	**9.41%**	**7.83%**	**11.18%**	**9.66%**	**9.38%**	**8.54%**	**18.86%**	**2.92%**		

Maximum Peak pressure

**Dog 1-12**		**1**	**2**	**3**	**4**	**5**	**6**	**7**	**8**	**9**	**10**	**11**	**12**
	LF	126,1	119,3	227,4	107,9	114,5	119,2	95,2	125,8	163,1	122,6	158,5	118,8
	LF	117,5	119,1	192,1	99,2	129,1	118,2	90,2	103,8	167	119	154,7	101,2
	LF	152	120	170,4	101,4	122,1	140,9	88,9	129,6	171	115,8	149,1	92,9
	LF	163,1	118,7	188,2	116,1	109,5	117,7	87,4	114,9	166,9	139,4	151,3	94,5
	LF	137,7	115,4	190,2	116,4	117,7	112	85,3	123,1	159,1	124,1	138,8	107,6
	LF	150	129,7	205,7	106,3	126,5	126,6	81,1	117,2	165,2	119,8	146,8	103,6
	**CV**	**12.18%**	**4.03%**	**9.82%**	**6.68%**	**6.19%**	**8.31%**	**5.40%**	**7.76%**	**2.44%**	**6.75%**	**4.55%**	**9.19%**
	RF	129	143	133,5	109,7	119,7	99,4	75,4	124,5	148,3	90,9	114,6	104,2
	RF	136,8	150,3	135,1	123,3	121,2	113,6	66,9	118,9	151,1	87,7	159	97,4
	RF	169,7	167,2	140	95,8	127,6	116,8	77,1	111,5	152	93,3	128,4	95,9
	RF	135,4	150,7	121,6	98	137,9	126,7	67,9	119,3	168	104,7	103,1	96,5
	RF	116,5	158	142,5	105,3	140,5	107,3	64,6	102,8	151,7	98	206,9	107
	RF	140,4	142	132,1	96,4	136,9	114,3	73,8	108,5	163,8	94,3	144,8	100,2
	**CV**	**12.81%**	**6.26%**	**5.45%**	**10.14%**	**6.91%**	**8.13%**	**7.24%**	**7.04%**	**5.15%**	**6.26%**	**26.12%**	**4.51%**
	LH	82,5	97,9	99	89	89,3	100	106,4	83,9	121,8	72,7	116,6	58
	LH	99,7	116,1	163,2	88,5	97,5	85,6	92,3	79,4	137,2	68,8	176,8	65,5
	LH	92,4	108,8	102	84,9	90,1	95,4	108	79,9	104,7	72,4	113,4	64,2
	LH	100,2	110,2	105,6	122,4	82,6	81,6	81,7	86,8	117,9	67,4	110,5	64,5
	LH	88,6	105,5	109,4	87,1	92,8	97,3	86,8	79	96,8	74,3	166,3	67,8
	LH	123,1	82,4	93,3	79,1	87,7	97,6	84,1	82,6	113,2	73,3	102,7	63,8
	**CV**	**14.45%**	**11.53%**	**22.88%**	**16.77%**	**5.55%**	**8.04%**	**12.23%**	**3.75%**	**12.19%**	**3.83%**	**24.33%**	**5.09%**

	RH	79,2	110,3	105,2	81,3	93,6	99,9	95,2	91,5	136,9	72,9	112,9	67,2
	RH	85,1	90,1	99	94,9	88,4	89,4	86,1	75,6	101,6	66,8	174,7	72,1
	RH	80,5	96	91,5	74,8	89,2	107,9	91,1	87,5	98,8	75,3	142,3	62,1
	RH	97,2	83,8	105,9	76,7	95,8	101,8	82,1	73,7	105,2	82,7	111,7	69,1
	RH	87,1	107,7	110,9	92,6	87,4	111,2	87,4	89,2	112,2	76,2	199,4	60
	RH	85,5	106,6	103,2	76,4	80,3	96,2	79,2	83,8	113,6	78,2	109,1	66,5
	**CV**	**7.45%**	**10.87%**	**6.51%**	**10.63%**	**6.06%**	**7.80%**	**6.71%**	**8.81%**	**12.37%**	**7.07%**	**26.80%**	**6.74%**
**Dog 13-21**		**13**	**14**	**15**	**16**	**17**	**18**	**19**	**20**	**21**			
	LF	149,5	118,7	94,6	136,8	112,4	157,3	138,5	116,4	112,5			
	LF	163,6	118,1	97,4	157,1	120	149,2	140,7	125,9	114,4			
	LF	153,7	120,6	108,1	159	99,9	161,8	140,4	110,3	107,7			
	LF	143,1	112,7	96,7	163,2	109,5	170,3	132,8	124,6	105,7			
	LF	142	118	80,1	164,9	139,7	159,2	159,4	123,5	100,7			
	LF	138	118,1	85	172,5	106	123,9	163	123,8	109,1			
	**CV**	**6.31%**	**2.24%**	**10.59%**	**7.61%**	**12.22%**	**10.47%**	**8.45%**	**5.06%**	**4.53%**			
	RF	124,8	150,2	114,7	157,4	90,4	139,8	137,5	119,7	125,3			
	RF	133,1	134,5	121,5	147,4	112,7	129,2	130,9	131,2	112			
	RF	139,2	143,5	94,8	159,2	118,8	133,6	131,9	143,9	117,3			
	RF	141,5	135,8	98,8	154,9	130,1	144,8	136,2	153,6	114,6			
	RF	158,3	135,7	107,8	149,3	125,3	155,4	136,8	134,9	111,6			
	RF	132,2	135,5	104,3	160	105,3	148,9	142,5	135,7	107,2			
	**CV**	**8.30%**	**4.53%**	**9.29%**	**3.40%**	**12.70%**	**6.86%**	**3.09%**	**8.42%**	**5.41%**			
	LH	128,9	64,7	68,7	138,4	93,8	142,8	118,5	108,4	85,2			
	LH	92,4	78,5	73,4	123	77,1	118	112,9	91,9	76,8			
	LH	78,8	59,8	67	123,4	94,8	122,3	137,5	90,4	82,5			
	LH	90,8	69	69,2	105,7	80,6	105,8	120	118,7	81,3			
	LH	81,8	69	76,9	105,2	71,5	94,2	115,8	98,4	91			
	LH	100,7	61,4	72,1	106,9	74,5	104,4	114,3	105,3	94,8			
	**CV**	**18.96%**	**10.08%**	**5.10%**	**11.48%**	**12.13%**	**14.93%**	**7.55%**	**10.54%**	**7.76%**			

RH	91,8	60,6	128,2	137,2	104,6	129,8	107,5	82,9	84,3
RH	98	55,4	106,5	117,8	80,4	117,5	103,2	117,5	78,9
RH	82,1	69,9	137,3	136,5	83,8	101	103	98,1	72,6
RH	96,3	69,9	106	124,2	95,4	120,8	110,4	100,4	83,9
RH	77,4	68,9	103	126,9	75,4	109,5	107	98,3	65,5
RH	95,8	68,4	95,3	133,4	83,3	135	104,8	94,5	80,2
**CV**	**9.42%**	**9.28%**	**14.45%**	**5.94%**	**12.38%**	**10.58%**	**2.70%**	**11.33%**	**9.38%**
